# Synthesis of Oligoribonucleotides Containing a 2′-Amino-5′-*S*-phosphorothiolate Linkage

**DOI:** 10.1021/acs.joc.1c01059

**Published:** 2021-09-17

**Authors:** Nan-Sheng Li, Selene C. Koo, Joseph A. Piccirilli

**Affiliations:** † Department of Biochemistry & Molecular Biology, University of Chicago, 929 East 57th Street, Chicago, Illinois 60637, Unites States; ‡ Department of Chemistry, University of Chicago, 929 East 57th Street, Chicago, Illinois 60637, Unites States

## Abstract

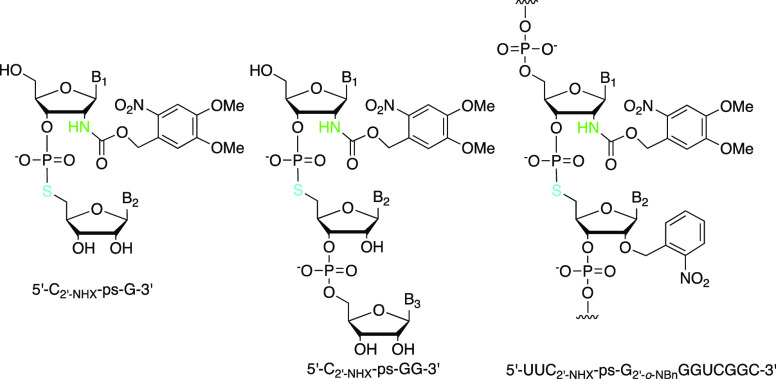

Oligoribonucleotides containing a photocaged 2′-amino-5′-*S*-phophorothiolate linkage have potential applications as therapeutic agents and biological probes to investigate the RNA structure and function. We envisioned that oligoribonucleotides containing a 2′-amino-5′-*S*-phosphorothiolate linkage could provide an approach to identify the general base within catalytic RNAs by chemogenetic suppression. To enable preliminary tests of this idea, we developed synthetic approaches to a dinucleotide, trinucleotide, and oligoribonucleotide containing a photocaged 2′-amino-5′-*S*-phosphorothiolate linkage. We incorporated the photocaged 2′-amino-5′-*S*-phosphorothiolate linkage into an oligoribonucleotide substrate for the hepatitis delta virus (HDV) ribozyme and investigated the pH dependence of its cleavage following UV irradiation both in the presence and absence of the ribozyme. The substrate exhibited a pH-rate profile characteristic of the modified linkage but reacted slower when bound to the ribozyme. Cleavage inhibition by the HDV ribozyme could reflect a non-productive ground-state interaction with the modified substrate’s nucleophilic 2′-NH_2_ or a poor fit of the modified transition state at the ribozyme’s active site.

## Introduction

**Figure 1:**
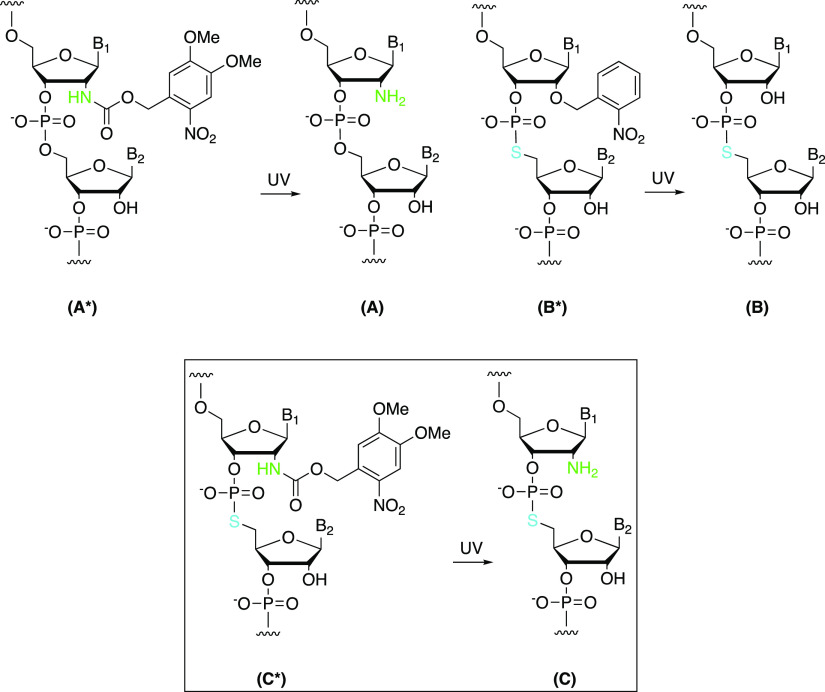
Oligoribonucleotides containing 2′-aminonucleotide (A), 5′-*S*-phosphorothiolate linkage (B), and 2′-amino-5′-*S*-phosphorothiolate linkage (C).

The synthesis of modified nucleosides, nucleotides, and oligonucleotides has been extensively investigated and motivated, in part, by creation of potential therapeutic agents (antisense, antiviral, and anticancer agents)^1−6^ and biological probes for the investigation of the relationship between the RNA structure and function.^7^ Chemical synthesis provides access to both naturally occurring and designed modified nucleotides and oligonucleotides, endowing biochemists and chemical biologists with tools to probe RNA chemistry and biology deeply and comprehensively.^8,9^ For example, the replacement of RNA’s 2′-OH with a 2′-NH_2_ (Figure 1A) maintains the hydrogen bonding capacity of 2′-OH but alters nucleophilicity, p*K*_a_, and metal-ion coordination properties.^10^ These defined changes in chemical properties form the basis of biochemical strategies to define the functional roles of RNA’s 2′-hydroxyl groups at specific locations. Owing to the weak nucleophilicity of the amino group toward the adjacent phosphodiester bond, 2′-amino substitution renders the ribose phosphate backbone inert to cleavage via internal transphosphorylation.^11^ Analogously, substitution of the 5′-bridging oxygen atom of the phosphodiester linkage with a sulfur atom (Figure 1B) alters hydrogen bonding, metal-ion coordination properties, and leaving group ability. However, in contrast to the 2′-amino group, a 5′-sulfur renders the phosphodiester backbone much more susceptible to transphosphorylation, owing to the greater leaving ability of sulfur relative to oxygen. This hyperactivation of the leaving group underpins a chemogenetic strategy to identify groups that activate the 5′-oxygen leaving group within the active site of a biological catalyst.^12−14^

These 2′-NH_2_ and 5′-*S*-RNA modifications have been used independently in studies of RNA, including as mechanistic probes for ribozyme-catalyzed reactions.^12−32^ Many reports have described the synthesis and incorporation of 2′-amino-modified nucleosides or nucleotides into RNA, including as a photocaged precursor (Figure 1A,A*).^15−31^ Nevertheless, 2′-NH_2_ substitution of the nucleophilic 2′-OH at the cleavage site of an endonucleolytic ribozyme has limited use as a mechanism probe on its own because the modification essentially abolishes cleavage.^27,32^ In contrast, the inherent instability of RNA containing a 5′-*S*-phosphorothiolate linkage makes working with this modification more challenging (Figure 1B). Protection of 2′-hydroxyl with a photolabile group such as an *o*-nitrobenzyl group, which could be removed by UV irradiation, has facilitated the use of this modification (Figure 1B*).^12−14^ We previously developed a strategy to identify the general acid in an enzymatic reaction using sulfur substitution of the leaving group.^12,13^ The better leaving ability of the sulfur obviates the need for general acid catalysis. As a consequence, mutations to the general acid that adversely affect catalysis in the context of the natural oxygen leaving group become suppressed in the context of the sulfur leaving group, whereas mutations elsewhere remain deleterious.

We have been interested in an analogous strategy to identify a potential general base in catalysis. However, there appear to be no simple chemical modifications of the 2′-hydroxyl group that would suppress the need for a general base. A nucleotide analogue whose nucleophilic hydroxyl group ionizes fully within the pH range of the ribozyme reaction could provide a suitable probe, but this is not obviously accessed within the nucleotide framework. An amino group represents another possibility, but as noted above, oligonucleotides bearing 2′-amino groups do not undergo backbone cleavage via attack of the nitrogen at the adjacent phosphorus center.

**Figure 2:**
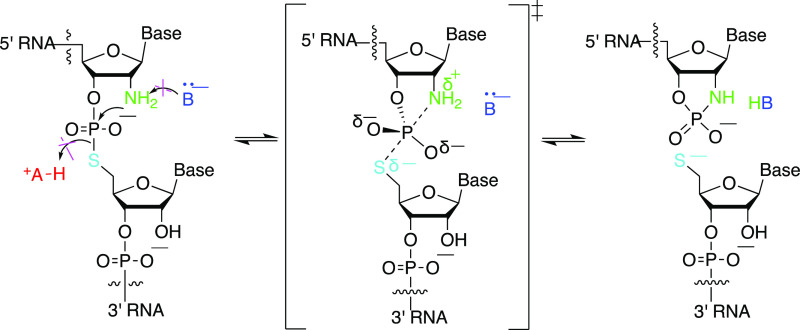
Possible mechanism of ribozyme-catalyzed cleavage of the RNA substrate containing a 2′-NH_2_/5′-*S* linkage at the cleavage site at pH > 7.

Alternatively, Eckstein and co-workers have shown that cleavage of a dinucleotide with a 2′-amino group can occur readily when the adjacent phosphorus bears a 5′-sulfur leaving group (*k* ∼ 10^–4^ s^–1^ with half-life time ∼2 h).^33^ Moreover, the cleavage reaction occurs independently of pH at pH values >7, indicating no susceptibility of the linkage to base catalysis. Accordingly, mutations that disable the ability of a ribozyme to deprotonate the nucleophile would be expected to affect cleavage of a substrate containing a 2′-amino group nucleophile and a 5′-*S* leaving group less adversely than a substrate containing only the 5′-*S* leaving group (Figure 2). Testing this approach in a ribozyme reaction requires installation of 2′-NH_2_, 5′-*S* modifications beyond dinucleotides and into oligoribonucleotides. Here, we report the synthesis of oligoribonucleotides containing a photocaged 2′-amino-5′-*S*-phosphorothiolate linkage (Figure 1C*) and determine its cleavage rate versus pH in the presence and absence of the hepatitis delta virus (HDV) ribozyme.

## Results and Discussion

We have previously reported approaches to synthesize RNA containing 2′-*O*-photocaged 5′-*S*-phosphorothiolate linkages using either 5′-*S*-phosphoramidite chemistry^34^ or ligations to a synthetic dinucleotide containing the modified linkage: (5′-C_2′-*O*-*o*-NBn_-ps-G-3′).^35^ We adapted these two strategies to enable the synthesis of RNAs containing 2′-amino-5′-*S*-phosphorothiolate linkages. We prepared the 2′-photocaged 2′-amino-5′-*S*-dinucleotide (5′-C_2′-NHX_-ps-G-3′), the trinucleotide derivative (5′-C_2′-NHX_-ps-GG-3′), and 2′-photocaged 2′-amino-3′-phosphoramidites.

### Synthesis of 2′-Photocaged 2′-Aminocytidine Phosphoramidites and a Dinucleotide Containing a Photocaged 2′-Amino-5′-*S*-phosphorothiolate Linkage (5′-C_2′-NHX_-ps-G-3′)

**Scheme 1:**
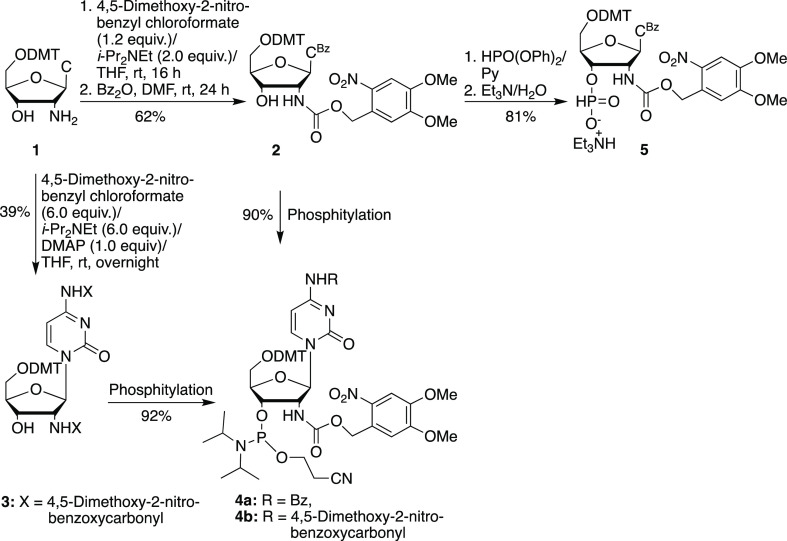


Photocaged 2′-aminocytidine 3′-phosphoramidites (**4a**, **4b**) and the corresponding 3′-H phosphonate (**5**) were synthesized as shown in Scheme 1. 5′-*O*-DMTr-2′-aminocytidine **1** was prepared according to a literature procedure.^16^ The 2′-amino group of **1** could be selectively protected using a large photocaging group (4,5-dimethoxy-2-nitrobenzyloxycarbonyl), followed by benzoyl protection of the amino group on the cytosine ring to afford compound **2**. The nucleoside derivative **2** was further converted to phosphoramidite **4a** or the 3′-*H*-phosphonate **5** in good yield. If excess 4,5-dimethoxy-2-nitrobenzyl chloroformate (6 equiv) was used in the reaction of **1**, the exocyclic amine of the cytosine ring also became photocaged, giving derivative **3**, which could be converted to the corresponding double-photocaged phosphoramidite **4b** in 36% overall yield.

**Scheme 2:**
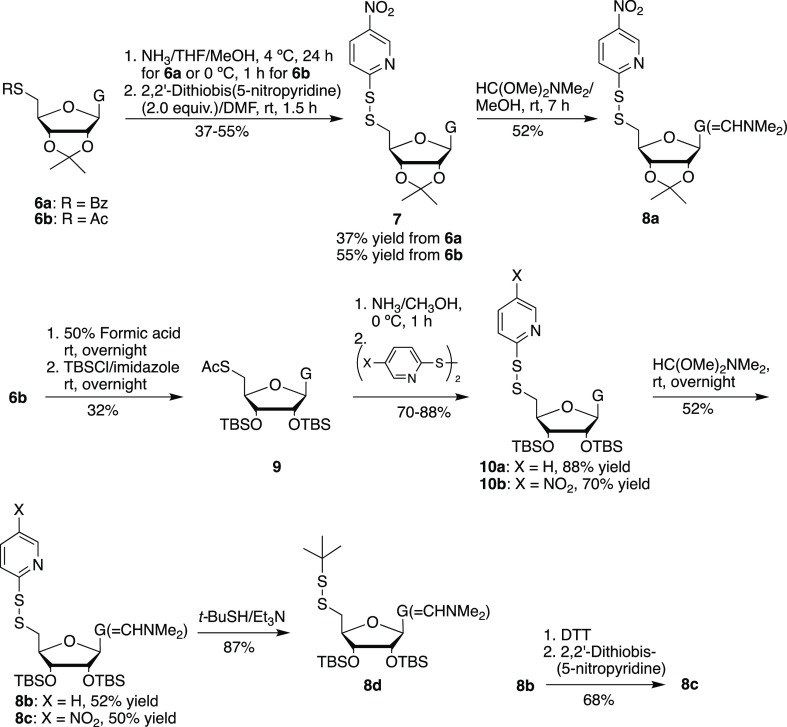


**Scheme 3:**
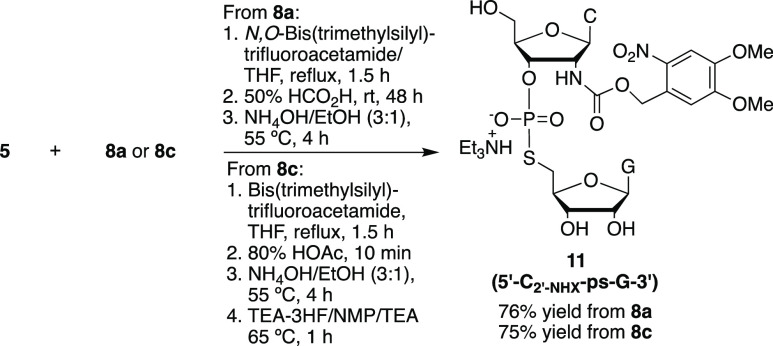


To prepare a 2′-photocaged 5′-*S* dinucleotide, various protected 5′-disulfanylguanosine derivatives (**8a**, **8b**, **8c**, and **8d**) were synthesized from compounds **6a**/**6b**^35^ as shown in Scheme 2 and reacted with 3′-*H*-phosphonate **5** (Scheme 3). However, only **8a** and **8c** containing the facile 5-nitro-2-pyridinyl leaving group reacted efficiently with 3′-*H*-phosphonate **5** to afford the 2′-photocaged 5′-*S*-dinucleotide (5′-C_2′-NHX_-ps-G-3′) (Scheme 3).

**Figure 3:**
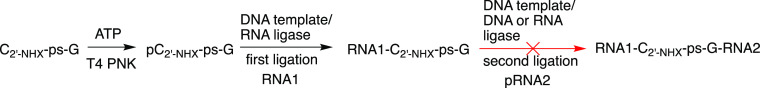
Construction of RNA-containing 5′-C_2′-NHX_-ps-G-3′ by a consecutive ligation approach.

Attempts to prepare an oligonucleotide containing a photocaged 2′-amino-5′-*S*-phosphorothiolate linkage from the dinucleotide 5′-C_2′-NHX_-ps-G-3′ through enzymatic ligation were not successful (Figure 3). We were able to install the 5′-phosphate enzymatically onto the dinucleotide to obtain 5′-pC_2′-NHX_-ps-G-3′ and successfully ligate it to RNA. However, the second ligation step failed to afford the full-length RNA, possibly because the large photocaged protecting group hinders the capacity of the oligonucleotide to serve as an acceptor substrate in the enzymatic ligation reaction.^36,37^ We hypothesized that an oligonucleotide bearing the large photocaged group more distal to the acceptor site might serve as a better acceptor substrate for ligation. To test this idea, we set out to prepare the trinucleotide, that is, 5′-C_2′-NHX_-ps-GG-3′, for incorporation into RNA via the two-step ligation approach.^35^

### Synthesis of a Trinucleotide Containing a Photocaged 2′-Amino-5′-*S*-phosphorothiolate Linkage (**13**: 5′-C_2′-NHX_-ps-GG-3′)

**Scheme 4:**
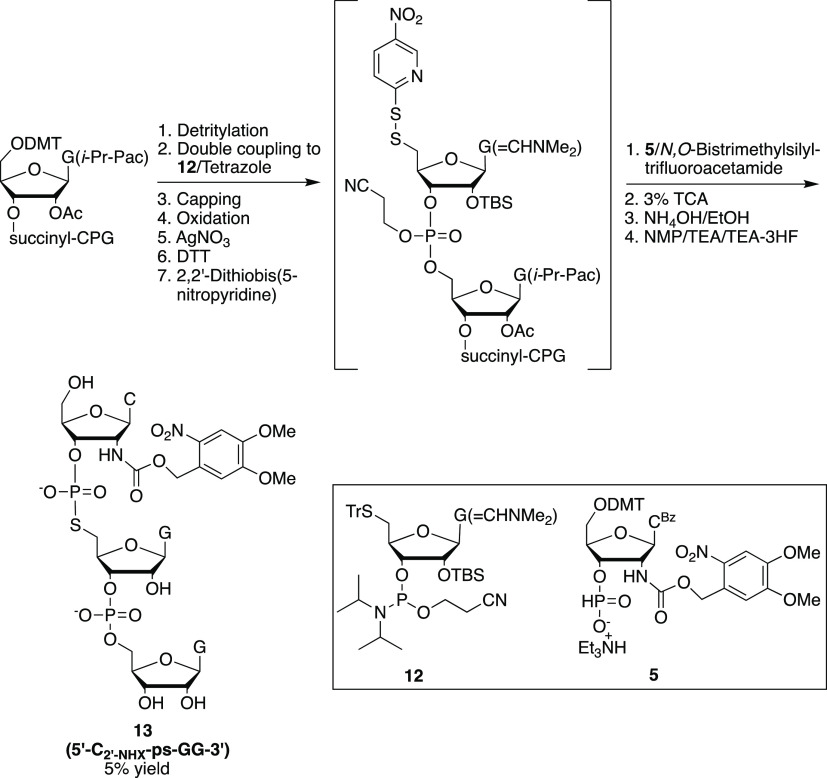


**Scheme 5:**
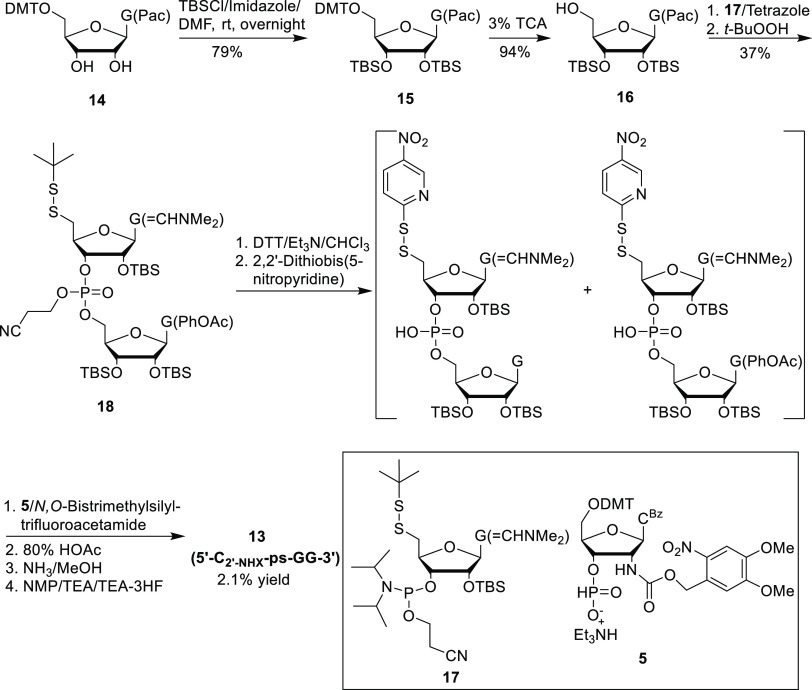


We developed two synthetic methods to prepare this trinucleotide using solid-phase and solution-phase approaches as shown in Schemes 4 and 5, respectively. For the solid-phase approach (Scheme 4), after detritylation with trichloroacetic acid solution, the commercially available rG-CPG solid support was coupled to 5′-*S*-guanosine phosphoramidite **12**(34) and deprotected manually by treatment of AgNO_3_ and 2,2′-dithiobis(5-nitropyridine) to afford an active disulfide. The disulfide intermediate was then coupled to 3′-*H*-phosphonate **5**. Subsequent deprotection and removal from the solid support afforded 5′-C_2′-NHX_-ps-GG-3′ (**13**) in 5% overall yield. In Scheme 5, the 2′,3′-*O*-TBS-guanosine derivative (**16**), prepared from 5′-*O*-DMTr-guanosine derivative **14**(38) in two steps (74% yield), was coupled to 5′-*tert*-butyl disulfide phosphoramidite **17**(34) to afford the dinucleotide derivative **18** in 37% yield. The 5′-*tert*-butyl disulfide dinucleotide **18** was then converted to an active 5-nitro-2-pyridinyl disulfide intermediate, which was then coupled to 3′-*H*-phosphonate **5** to afford 5′-C_2′-NHX_-ps-GG-3′ (**13**) in 2.1% overall yield.

**Scheme 6:**
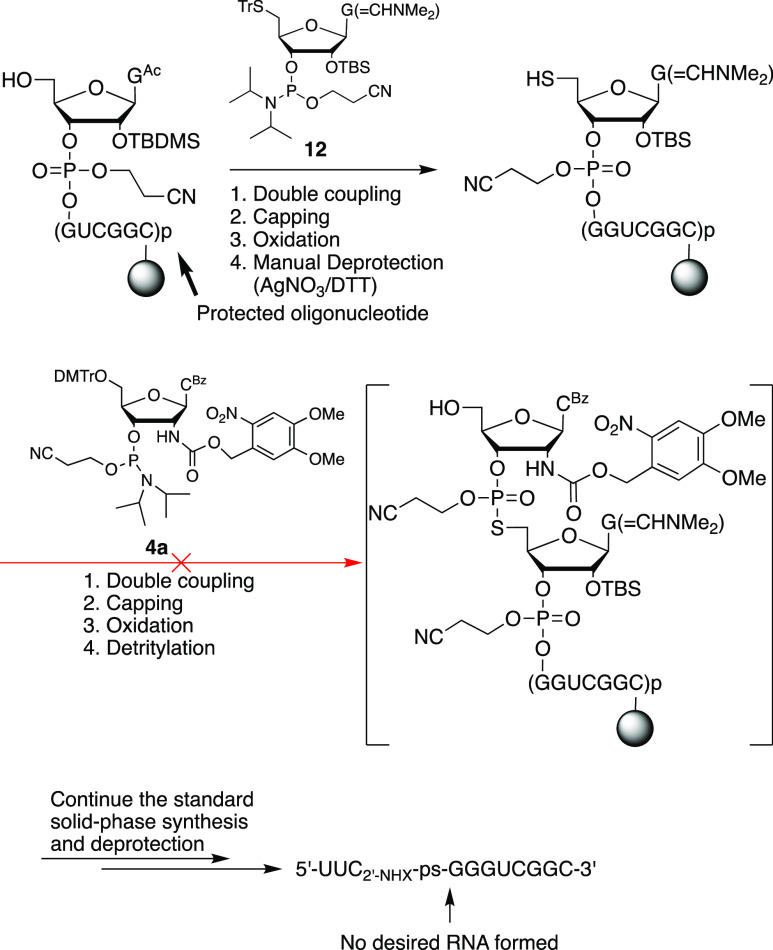


**Scheme 7:**
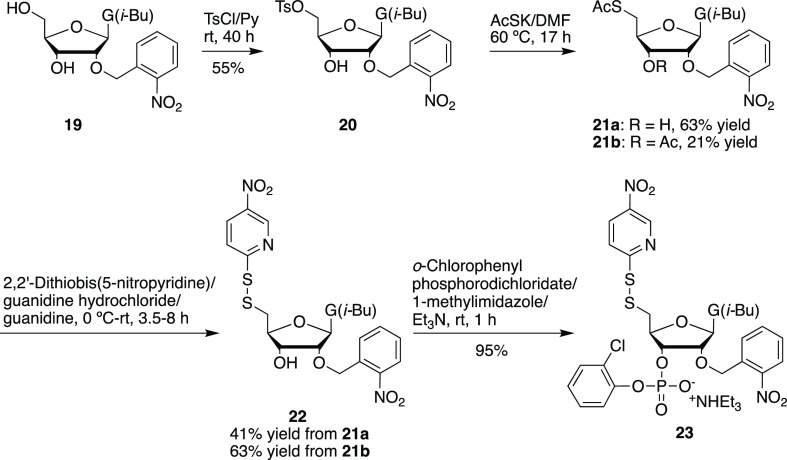


**Scheme 8:**
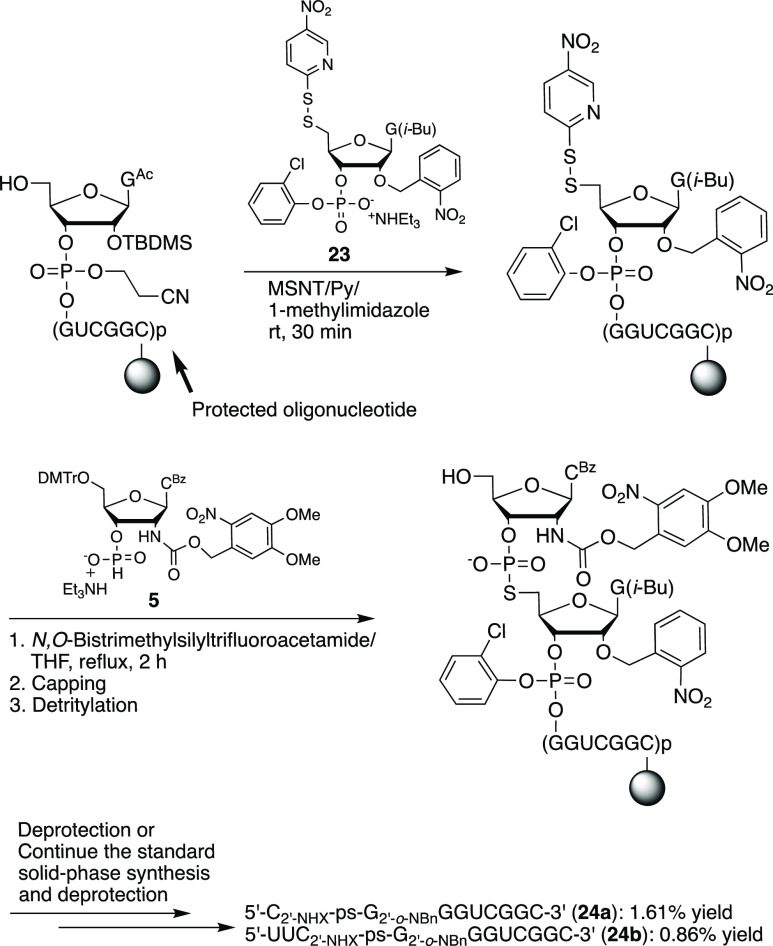


**Scheme 9:**
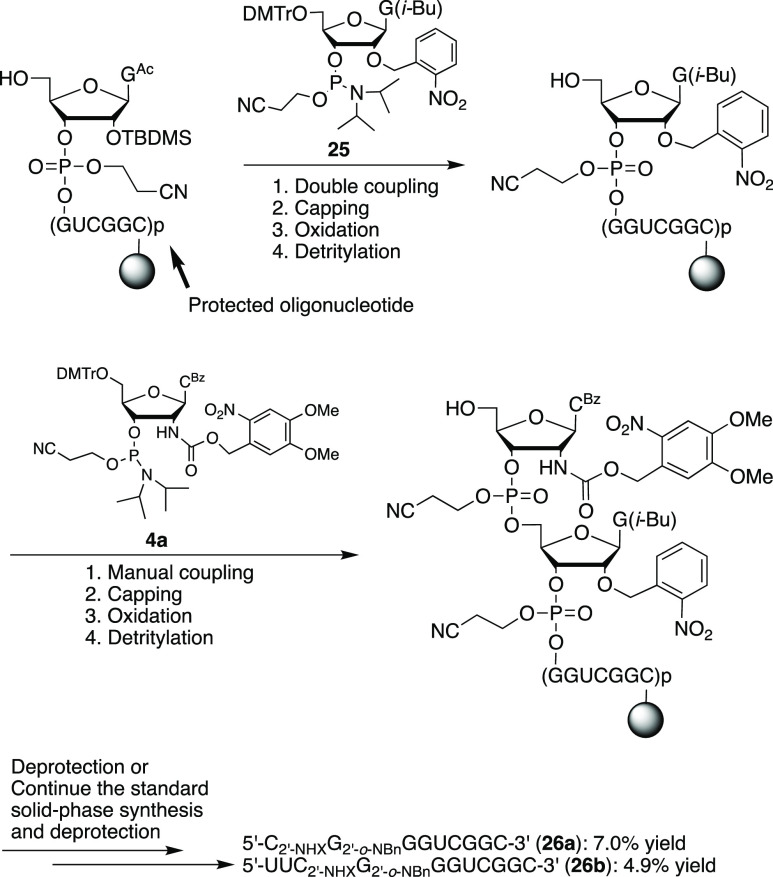


Unfortunately, the trinucleotide **13** synthesized either by solid-phase synthesis or by solution methods still failed to afford the full-length RNA due to the failure of the second ligation step of our two-step ligation approach. We then investigated a possible solid-phase synthetic approach.

### Solid-Phase Synthesis of Oligonucleotides Containing a Photocaged 2′-Amino-5′-*S*-phosphorothiolate Linkage (Schemes 6–9)

Following our reported solid-phase synthesis protocols for oligonucleotides containing a photocaged 2′-*O*-*o*-nitrobenzyl-5′-*S*-phosphorothiolate linkage,^34^ the 5′-*O*-detritylated undeprotected oligonucleotide (5′-GGUCGGC-CPG) on solid support was first coupled to 5′-*S*-guanosine phosphoramidite (**12**) and then coupled to 2′-photocaged aminocytidine phosphoramidite (**4a**) as shown in Scheme 6. The solid-phase synthesis was continued for two additional cycles. However, after the standard workup procedures, we could not detect the desired 11-mer oligonucleotide, most likely due to inefficient coupling of the 5′-SH group to the 2′-photocaged aminocytidine phosphoramidite **4a** (Scheme 6). We have tested the reaction of the support-bound free 5′-SH with 2,2′-dithiobis(5-nitropyridine) to form the disulfide in situ, followed by coupling with 3′-*H*-phosphonate **5**. However, this approach failed to produce the desired full-length RNA, possibly due to inefficient disulfide formation. To circumvent these problems, we resorted to phosphonate coupling inspired by the synthesis of 2′-azide RNA.^39^ First, we prepared 2′-*O*-*o*-nitrobenzyl-5′-disulfanyl-3′-phosphonate **23** in four steps from 2′-*O*-*o*-nitrobenzyl-*N*^2^-isobutyryl guanosine (**19**)^35^ (Scheme 7). Following solid-phase synthesis of 3′-CPG-rCGGCUGG and 5′-detritylation, we coupled phosphonate **23**, followed by 3′-*H*-phosphonate **5**. Standard deprotection or continuation of solid-phase synthesis followed by standard deprotection yielded the RNAs containing a photocaged 2′-amino-5′-*S*-phosphorothiolate linkage (**24a** and **24b**) (Scheme 8).

The corresponding RNAs containing a photocaged 2′-amino-5′-*O*-phosphonate linkage (**26a** and **26b**) were prepared by the solid-phase synthesis with the first coupling to phosphoramidite **25**(40) and then coupling to **4a** (Scheme 9).

### Characterization and pH-Dependent Cleavage of a Ribozyme Substrate Containing a 2′-Amino-5′-*S*-phosphorothiolate Linkage

**Figure 4:**
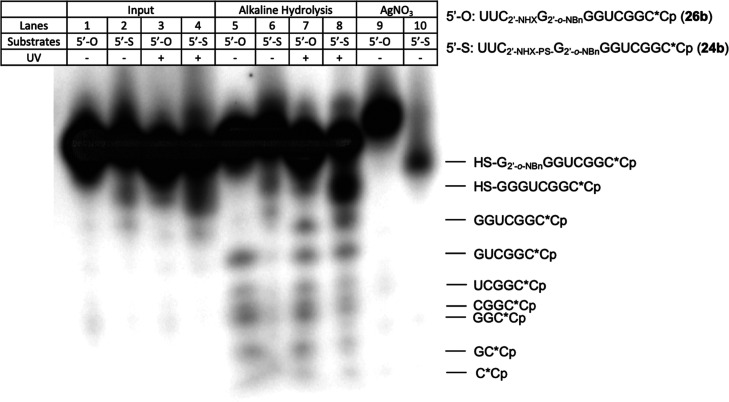
Characterization of 3′-radiolabeled RNAs (11 mer) containing 2′-amino-5′-*O*- (**26b**) or 2′-amino-5′-*S*-linkage (**24b**). The figure was depicted from the right side of a large gel, so the oligo on lane 9 moved a little bit slower than the same oligo on lanes 1 and 5.

All photocaged RNA oligonucleotides **24a**, **24b**, **26a**, and **26b** were analyzed by MALDI-TOF mass spectrometry, confirming their molecular weights. HPLC confirmed that under neutral conditions, **26a** and **26b** were photodeprotected to the corresponding 5′-C_2′-NH2_-GGGUCGGC-3′ (∼25% conversion) and 5′-UUC_2′-NH_2__-GGGUCGGC-3′ (∼30% conversion) after UV irradiation (365 nm, 15–30 min). The UV deprotection rates were *k*_(**26a**)_ = 0.27 min^–1^ and *k*_(**26b**)_ = 0.065 min^–1^, respectively. The photodeprotection of the shorter oligonucleotide (**26a**, 9 mer) occurred about 4 times faster than the longer oligonucleotide (**26b**, 11 mer). After 3′-radiolabeling, the RNA oligonucleotides **24b** and **26b** were treated with Ag^+^ solution. As expected, **24b** cleaves in the presence of Ag^+^ ion, confirming the presence of the phosphorothiolate linkage (Figure 4, lane 10), but **26b**, which contains no phosphorothiolate linkage, was unaffected in the presence of Ag^+^ ion (Figure 4, lane 9). Additionally, comparison of the alkaline hydrolysis of **24b** and **26b** before and after UV irradiation also confirmed the 2′-NH_2_-mediated cleavage of the 5′-phosphorothiolate linkage in **24b** (Figure 4, lanes 7 and 8).

**Figure 5:**
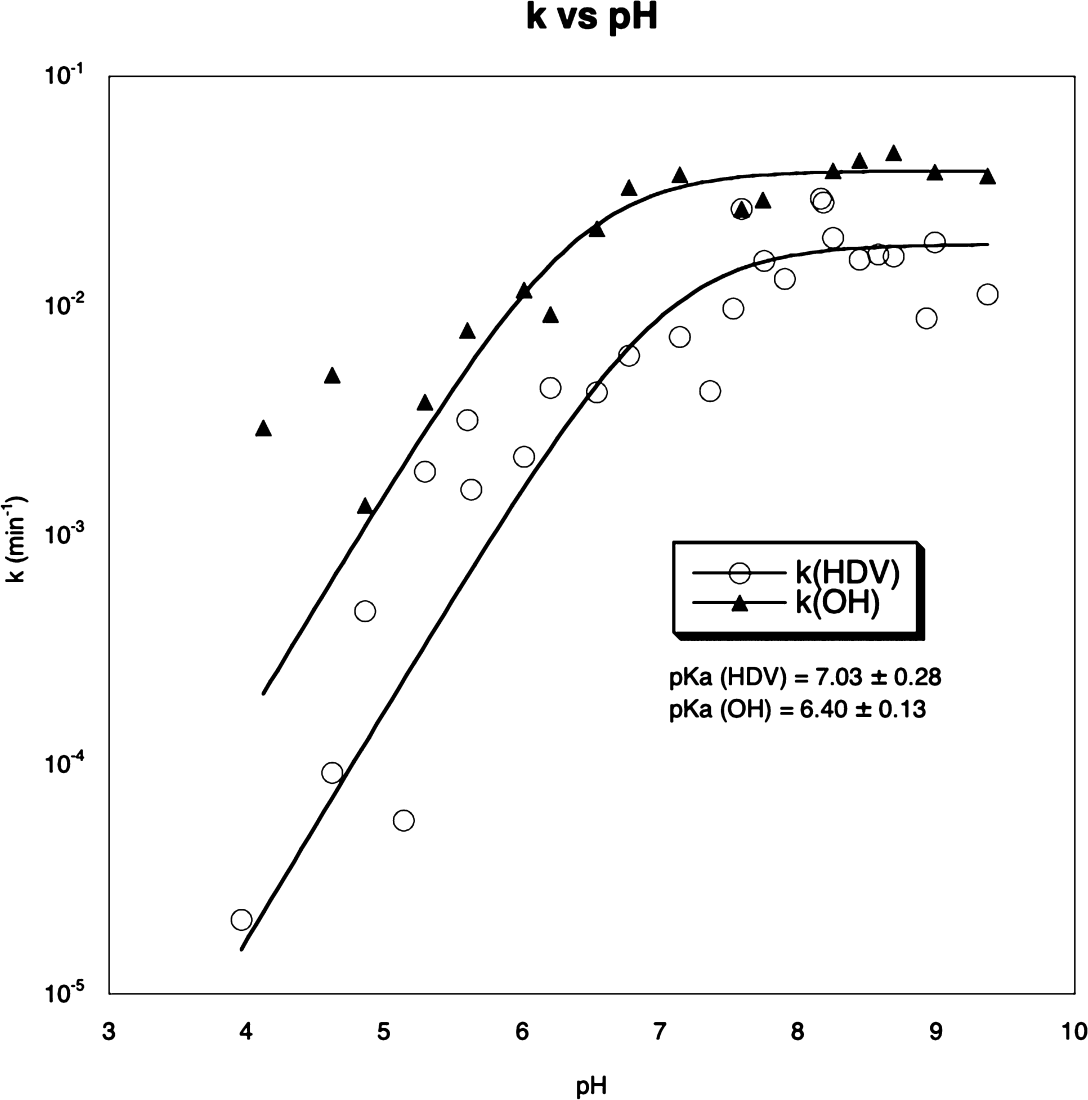
pH rate profiles of cleavages of 5′-radiolabeled **24b** in the presence/absence of HDV ribozyme and 10 mM MgCl_2_. *k*(HDV) (○): cleavage rate in the presence of HDV ribozyme. *k*(OH) (▲): cleavage rate in the absence of HDV ribozyme.

We then studied the pH-dependent cleavage reaction of 5′-radiolabeled **24b** in the presence and absence of the anti-genomic HDV ribozyme^12,41^ (1 μM) and 10 mM MgCl_2_ (Figure 5). As expected, the cleavage rate of **24b** increases in a log-linear fashion at pH values below the p*K*_a_ of the 2′-amino group and becomes independent of pH at pH values above the p*K*_a_ (6.2).^42^ This pH rate profile resembles that for the cleavage of the corresponding U_2′-NH_2__-ps-U dinucleotide.^33^ We found that in the presence of the HDV ribozyme, **24b** underwent cleavage 3–9-fold slower than in the absence of the HDV ribozyme throughout the tested pH range. This result indicates that ribozyme binding to the substrate inhibits cleavage of the 2′-amino-5′-*S*-phosphorothiolate linkage. The inhibition may reflect a non-productive ground-state interaction involving 2′-OH in the natural reaction.^43−46^ The possible non-productive ground-state interactions in the enzyme substrate complex most likely involve hydrogen bonding or metal coordination to the nucleophilic amino group. These interactions could diminish nucleophilicity through interaction with the amino group’s lone pair of electrons or disfavor acquisition of the in-line conformation required for reaction.^46^ Alternatively, the HDV ribozyme may not be able to accommodate the transition state for 2′-*N*-transphosphorylation of the 2′-amino-5′-*S*-phosphorothiolate linkage. We have shown previously using model systems that amine nucleophiles react at phosphodiesters bearing sulfur-leaving groups via expanded transition states, with less bonding to both the nucleophile and the leaving group, relative to analogous reactions of phosphodiesters bearing oxygen leaving groups.^47^ Possibly, the expanded transition state does not fit well at the HDV-active site, resulting in slower cleavage relative to the corresponding reaction in the absence of ribozyme.

## Conclusions

We have prepared RNAs containing a C_2′-NH_2__-psG linkage by solid-phase synthesis using 2′-photocaged 5′-disulfanyl guanosine derivative **23** and 2′-aminophotocaged cytidine 3-*H* phosphonate **5**. The structures of these modified RNAs were confirmed by MS and Ag^+^ treatment. In the context of a trans-acting HDV ribozyme substrate, the modified linkage exhibited the expected pH-dependent cleavage in the absence of ribozyme, verifying its integrity. Unexpectedly, instead of facilitating substrate cleavage, the HDV ribozyme inhibited cleavage of the modified substrate, possibly reflecting non-productive ground-state interactions or poor accommodation of the transition state within the RNA active site.

## Experimental Section

### 2′-Amino-*N*^4^-benzoyl-2′-*N*-(4,5-dimethoxy-2-nitrobenzoxycarbonyl)-5′-*O*-DMTr-cytidine (**2**)

Under argon to a solution of 2′-amino-5′-*O*-DMTr-cytidine (**1**)^16^ (150 mg, 0.27 mmol) in tetrahydrofuran (THF) (3 mL), diisopropylethylamine (94 μL, 0.54 mmol) and 4,5-dimethoxy-2-nitrobenzyl chloroformate (91 mg, 0.33 mmol) were added. The reaction mixture was stirred at rt for 16 h. The solvent was removed, and the residue was isolated by silica gel chromatography, eluting with 5% methanol in chloroform to afford intermediate 2′-amino-2′-*N*-(4,5-dimethoxy-2-nitrobenzoxycarbonyl)-5′-*O*-DMTr-cytidine: 185 mg as a light yellow foam. HRMS (ESI/APCI) *m*/*z*: [M + Na]^+^ calcd for C_40_H_41_N_5_O_12_Na, 806.2649; found, 806.2639). To a solution of 2′-amino-2′-*N*-(4,5-dimethoxy-2-nitrobenzoxycarbonyl)-5′-*O*-DMTr-cytidine (185 mg, 0.236 mmol) in dimethylformamide (DMF) (5.0 mL), benzoyl anhydride (90 mg, 0.35 mmol) was added and the mixture was stirred at rt for 24 h. The reaction was quenched with methanol (1.0 mL). After 10 min, the mixture was evaporated under reduced pressure. The residue was dissolved in ethyl acetate and the organic solution was washed with 5% NaHCO_3_, brine, and dried over magnesium sulfate. The solvent was removed, and the residue was isolated by silica gel chromatography, eluting with 4% methanol in dichloromethane to afford **2** as a light yellow foam: 0.148 g (62% yield). ^1^H NMR (500 MHz, CDCl_3_/TMS): δ 9.21 (br s, 1H), 8.10 (d, 1H, *J* = 5.6 Hz), 7.89 (d, 2H, *J* = 6.0 Hz), 7.60–7.15 (m, 14H), 6.98 (s, 1H), 6.85–6.70 (m, 5H), 6.49 (d, 1H, *J* = 6.0 Hz), 5.50–5.30 (m, 2H), 4.59 (m, 2H), 4.35 (m 1H), 3.87 (s, 3H), 3.83 (s, 3H), 3.76 (s, 6H), 3.46 (m, 2H); ^13^C{^1^H} NMR (126 MHz, CDCl_3_): δ 162.7, 158.7, 156.1, 153.7, 147.9, 144.1, 139.2, 135.4, 135.1, 133.1, 130.11, 130.07, 128.9, 128.2, 128.1, 127.8, 127.1, 113.4, 109.7, 108.0, 87.2, 87.1, 86.1, 71.8, 63.9, 63.7, 60.6, 56.6, 56.2, 55.2; HRMS (ESI/APCI) *m*/*z*: [M + Na]^+^ calcd for C_47_H_45_N_5_O_13_Na, 910.2912; found, 910.2908.

### 2′-Amino-2′-*N*,*N*^4^-di(4,5-dimethoxy-2-nitrobenzoxycarbonyl)-5′-*O*-DMTr-cytidine (**3**)

Under argon to a solution of 2′-amino-5′-*O*-DMTr-cytidine (**1**)^16^ (162 mg, 0.30 mmol) in THF (10 mL), diisopropylethylamine (314 μL, 1.80 mmol), DMAP (37 mg, 0.30 mmol), and 4,5-dimethoxy-2-nitrobenzyl chloroformate (492 mg, 1.80 mmol) were added. The reaction mixture was stirred at rt overnight. Thin-layer chromatography (TLC) showed that the reaction was complete, and the reaction was quenched with methanol (1.0 mL). The solvent was removed, and the residue was isolated by silica gel chromatography, eluting with 3% methanol in chloroform to afford **3** as a yellow foam: 119 mg (39% yield). ^1^H NMR (400 MHz, CDCl_3_/TMS): δ 8.01 (br s, 1H), 7.71 (s, 1H), 7.59 (s, 1H), 7.45–6.90 (m, 11H), 6.82 (d, 4H, *J* = 8.4 Hz), 6.38 (d, 1H, *J* = 7.8 Hz), 5.58 (s, 2H), 5.49 (d, 1H, *J* = 15.0 Hz), 5.36 (d, 1H, *J* = 15.0 Hz), 4.57 (m, 1H), 4.47 (br s, 2H), 4.29 (m, 1H), 4.00–3.70 (m, 18H), 3.43 (m, 2H); ^13^C{^1^H} NMR (101 MHz, CDCl_3_): δ 158.6, 156.1, 153.7, 153.6, 148.3, 147.8, 143.9, 139.5, 135.2, 134.9, 130.0, 128.0, 127.9, 127.0, 113.2, 110.2, 108.5, 107.8, 87.0, 85.9, 71.7, 64.6, 63.8, 63.5, 60.6, 56.5, 56.4, 56.3, 56.1, 55.1; HRMS (ESI/APCI) *m*/*z*: [M + Na]^+^ calcd for C_50_H_50_N_6_O_18_Na, 1045.3079; found, 1045.3076.

### 2′-Amino-*N*^4^-benzoyl-2′-*N*-(4,5-dimethoxy-2-nitrobenzoxycarbonyl)-5′-*O*-DMTr-cytidine 3′-*N*,*N*-Diisopropyl(cyanoethyl)phosphoramidite (**4a**)

To a solution of 2′-amino-*N*^4^-benzoyl-2′-*N*-(4,5-dimethoxy-2-nitrobenzoxycarbonyl)-5′-*O*-DMTr-cytidine (**2**) (144 mg, 0.162 mmol) and *i*-Pr_2_NEt (140 μL, 0.81 mmol) in anhydrous dichloromethane (5 mL) at 0 °C, ClP(NPr-*i*_2_)OCH_2_CH_2_CN (72 μL, 0.32 mmol) was added, followed by the addition of 1-methylimidazole (6.4 μL, 0.08 mmol). After stirring the reaction mixture at room temperature (rt) for 1 h, the reaction was quenched with methanol (1.0 mL). The solvent was removed, the residue was purified by silica gel chromatography, eluting with 5% CH_3_COCH_3_ in CH_2_Cl_2_ containing 0.5% Et_3_N to afford **4a** as a yellow foam: 158 mg (90% yield, > 95% purity). ^31^P{^1^H} NMR (162 MHz, CD_3_CN): δ 153.4, 152.6; HRMS (ESI/APCI) *m*/*z*: [M + Na]^+^ calcd for C_56_H_62_N_7_O_14_PNa, 1110.3990; found, 1110.3996.

### 2′-Amino-*N*^4^,2′-*N*-di(4,5-dimethoxy-2-nitrobenzoxycarbonyl)-5′-*O*-DMTr-cytidine 3′-*N*,*N*-Diisopropyl(cyanoethyl)phosphoramidite (**4b**)

To a solution of 2′-amino-2′-*N*,*N*^4^-di(4,5-dimethoxy-2-nitrobenzoxycarbonyl)-5′-*O*-DMTr-cytidine (**3**) (103 mg, 0.10 mmol) and *i*-Pr_2_NEt (87 μL, 0.50 mmol) in anhydrous dichloromethane (5 mL) at 0 °C, ClP(NPr-*i*_2_)OCH_2_CH_2_CN (45 μL, 0.20 mmol) was added, followed by the addition of 1-methylimidazole (4.0 μL, 0.05 mmol). After stirring the reaction mixture at rt for 1 h, the reaction was quenched with methanol (1.0 mL). The solvent was removed, and the residue was purified by silica gel chromatography, eluting with 2% CH_3_COCH_3_ in CH_2_Cl_2_ containing 0.5% Et_3_N to afford **4b** as a yellow foam: 113 mg (92% yield, >95% purity). ^31^P{^1^H} NMR (162 MHz, CD_3_CN): δ 151.1, 150.3; HRMS (ESI/APCI) *m*/*z*: [M + Na]^+^ calcd for C_59_H_67_N_8_O_19_PNa, 1245.4158; found, 1245.4153.

### 2′-Amino-*N*^4^-benzoyl-2′-*N*-(4,5-dimethoxy-2-nitrobenzoxycarbonyl)-5′-*O*-dimethoxytrityl-2′-deoxycytidine-3′-*H*-phosphonate (**5**)

To the solution of 2′-amino-*N*^4^-benzoyl-2′-*N*-(4,5-dimethoxy-2-nitrobenzoxycarbonyl)-5′-*O*-dimethoxytrityl-2′-deoxycytidine (**2**) (72 mg, 0.081 mmol) in pyridine (5 mL), diphenyl phosphite (77 μL, 0.41 mmol) was added. After 15 min, the reaction was quenched by addition of a mixture of water/triethylamine (1:1 v/v, 2 mL), and the resulting mixture was stirred for 15 min. The solvent was evaporated, and the residue was partitioned between dichloromethane (25 mL) and saturated aqueous NaHCO_3_ (10 mL). The organic layer was washed for additional two times with aqueous NaHCO_3_ (10 mL) and subsequently dried over MgSO_4_. Following the removal of the solvent by evaporation under vacuum, the resulting residue was purified by silica gel chromatography, eluting with 3% methanol in dichloromethane containing 3% of triethylamine to afford compound **5** (71 mg, 81% yield) as a light yellow solid. ^1^H NMR (400 MHz, CDCl_3s_/TMS): δ 8.05 (d, 1H, *J* = 7.2 Hz), 7.88 (d, 2H, *J* = 7.6 Hz), 7.73 (s, 1H), 7.68 (s, 1H), 7.59 (t, 1H, *J* = 7.6 Hz), 7.49 (t, 2H, *J* = 7.6 Hz), 7.41 (d, 1H, *J* = 7.6 Hz), 7.35–7.27 (m, 6H), 7.23 (t, 1H, *J* = 7.6 Hz), 7.09 (m, 2H), 6.85 (d, 4H, *J* = 8.8 Hz), 6.44 (d, 1H, *J* = 8.4 Hz), 5.60–5.40 (m, 2H), 4.92 (m, 1H), 4.67 (m, 1H), 4.48 (br s, 1H), 4.04 (s, 3H), 3.92 (s, 3H), 3.80 (s, 6H), 3.60–3.45 (m, 2H); ^13^C{^1^H} NMR (101 MHz, CDCl_3_): δ 162.1, 158.7, 156.0, 154.0, 147.9, 144.7, 144.1, 139.2, 135.2, 135.1, 133.1, 130.1, 129.3, 128.9, 128.1, 127.7, 127.2, 113.4, 109.8, 107.9, 87.3, 86.8, 84.8, 74.3, 63.65, 63.60, 58.6, 56.9, 56.3, 55.3; ^31^P{1H} NMR (162 MHz, CDCl_3_): δ 7.67; HRMS (ESI/APCI) *m*/*z*: [M + Na]^+^ calcd for C_47_H_45_N_5_O_15_P [M^–^] 950.2650, found 950.2653.

### 5′-Deoxy-2′,3′-*O*-isopropylidene-5′-(5-nitropyridinyl-2-disulfanyl)guanosine (**7**)

#### From 5′-Benzoylthio-5′-deoxy-2′,3′-O-isopropylideneguanosine (**6a**)^35^

A solution of **6a** (371 mg, 0.837 mmol) in THF (15 mL) and CH_3_OH (15 mL) was saturated with ammonia at 0 °C for 30 min, and the mixture was kept at 4 °C for 24 h. After removing the solvent, the residue was dried under vacuum for 30 min. The residue was then dissolved into DMF (15 mL). To the resulting solution, 2,2′-dithiobis(5-nitropyridine) (521 mg, 1.68 mmol) was added. The reaction mixture was stirred at rt for 1.5 h. The solvent was removed, and the residue was isolated by silica gel chromatography, eluting with 5% methanol in chloroform to afford **7** as a light yellow foam (155 mg, 37% yield).

#### From 5′-Acetylthio-5′-deoxy-2′,3′-*O*-isopropylideneguanosine (**6b**)^35^

Disulfide **7** was also prepared from **6b** according to the procedure from **6a** with a slight modification. A solution of **6b** (100 mg, 0.26 mmol) in THF (5 mL) and CH_3_OH (5 mL) was saturated with ammonia at 0 °C for 30 min, and the mixture was kept at 0 °C for additional 30 min (instead of at 4 °C for 24 h for **6a**). The solvent was removed, and the residue was dissolved into DMF (5 mL) and then reacted with 2,2′-dithiobis(5-nitropyridine) (161 mg, 0.52 mmol) to afford **7**(35) as a light yellow foam (71 mg, 55% yield). ^1^H NMR (400 MHz, DMSO-*d*_6_): δ 10.95 (br s, 1H), 9.15 (d, 1H, *J* = 2.8 Hz), 8.48 (dd, 1H, *J* = 2.8, 9.2 Hz), 8.23 (s, 1H), 7.88 (s, 1H), 6.77 (br s, 2H), 6.05 (s, 1H), 5.33 (d, 1H, *J* = 6.0 Hz), 5.15 (m, 1H), 4.32 (m, 1H), 3.32 (dd, 1H, *J* = 6.0, 14.0 Hz), 3.22 (dd, 1H, *J* = 8.4, 14.0 Hz), 1.46 (s, 3H), 1.29 (s, 3H); ^13^C{^1^H} NMR (101 MHz, DMSO-*d*_6_): δ 167.0, 155.7, 154.1, 149.7, 144.8, 142.2, 136.8, 132.4, 119.5, 114.6, 113.1, 89.8, 86.3, 83.8, 83.4, 40.5, 26.8, 25.2; HRMS (ESI/APCI) *m*/*z*: [M + H]^+^ calcd for C_18_H_20_N_7_O_6_S_2_, 494.0917; found, 494.0919.

### *N*^2^-[(Dimethylamino)methylene]-5′-deoxy-2′,3′-*O*-isopropylidene-5′-(5-nitropyridinyl-2-disulfanyl)guanosine (**8a**)

Under argon to a solution of **7** (62 mg, 0.126 mmol) in methanol (10 mL), *N*,*N*-dimethylformamide dimethyl acetal (0.167 mL, 1.26 mmol) was added. The mixture was stirred at rt for 7 h. The solvent was removed, and the residue was isolated by silica gel chromatography, eluting with 4% methanol in chloroform to afford **8a** as a light yellow foam: 36 mg (52% yield). ^1^H NMR (400 MHz, CDCl_3_/TMS): δ 9.91 (br s, 1H), 9.21 (s, 1H), 8.54 (s, 1H), 8.37 (d, 1H, *J* = 8.5 Hz), 7.81 (m, 1H, *J* = 8.5 Hz), 7.73 (s, 3H), 6.02 (d, 1H, *J* = 1.5 Hz), 5.42 (dd, 1H, *J* = 1.5, 6.2 Hz), 5.04 (dd, 1H, *J* = 3.0, 6.2 Hz), 4.46 (m, 1H), 3.30–3.05 (m, 2H), 3.23 (s, 3H) 3.14 (s, 3H), 1.64 (s, 3H), 1.40 (s, 3H); ^13^C{^1^H} NMR (101 MHz, CDCl_3_): δ 167.7, 158.1, 157.8, 157.0, 149.7, 145.1, 137.3, 131.8, 121.1, 119.7, 114.7, 90.0, 85.5, 84.4, 83.5, 41.8, 41.4, 35.4, 27.1, 25.4; HRMS (ESI/APCI) *m*/*z*: [M + H]^+^ calcd for C_21_H_25_N_8_O_6_S_2_, 549.1339; found, 549.1339.

### 5′-Acetylthio-5′-deoxy-2′,3′-*O*-di-(*tert*-butyldimethylsilyl)guanosine (**9**)

5′-aceylthio-5′-deoxy-2′,3′-*O*-isopropylideneguanosine (**6b**)^35^ (0.900 g, 2.36 mmol) in 50% formic acid (20 mL) was stirred at rt overnight. The solvent was removed, and the residue was co-evaporated with toluene and dried under vacuum. This residue was dissolved into DMF (40 mL). To the resulting solution, imidazole (3.68 g, 54.1 mmol) and TBSCl (1.63 g, 10.8 mmol) were added. The mixture was stirred at rt overnight. The solvent was removed, and the residue was dissolved into dichloromethane and washed with saturated NaHCO_3_ and brine. The solvent was removed, and the residue was purified by silica gel chromatography, eluting with 5% methanol in chloroform to afford **9** as a white foam: 0.412 g (33% yield). ^1^H NMR (400 MHz, CDCl_3_/TMS): δ 7.66 (s, 1H), 6.49 (br s, 2H), 5.71 (d, 1H, *J* = 6.0 Hz), 5.08 (dd, 1H, *J* = 4.4, 5.6 Hz), 4.20–4.05 (m, 2H), 3.64 (dd, 1H, *J* = 6.8, 14.0 Hz), 3.23 (dd, 1H, *J* = 6.8, 14.0 Hz), 2.39 (s, 3H), 0.95 (s, 9H), 0.82 (s, 9H), 0.13 (s, 3H), 0.12 (s, 3H), −0.03 (s, 3H), −0.19 (s, 3H); ^13^C{^1^H} NMR (101 MHz, CDCl_3_): δ 195.4, 159.3, 153.6, 151.5, 137.6, 118.3, 89.6, 84.0, 74.7, 73.5, 31.4, 30.7, 25.93, 25.85, 18.2, 18.0, −4.3, −4.5, −4.6, −5.0; HRMS (ESI/APCI) *m*/*z*: [M + H]^+^ calcd for C_25_H_44_N_5_O_4_SiS_2_, 570.2599; found, 570.2605.

### 5′-Deoxy-2′,3′-*O*-di-(*tert*-butyldimethylsilyl)-5′-(2-pyridinyldisulfanyl)guanosine (1**0a**)

A solution of **9** (162 mg, 0.293 mmol) in CH_3_OH (15 mL) was saturated with ammonia at 0 °C for 30 min and then kept at 0 °C for 30 min. After removing the solvent, the residue was dried under vacuum for 30 min. The residue was then dissolved into DMF (25 mL). To the resulting solution, 2,2′-dipyridyl disulfide (259 mg, 1.18 mmol) was added. The reaction mixture was stirred at 60 °C in an oil bath for 16 h. The solvent was removed, and the residue was isolated by silica gel chromatography, eluting with 5–10% methanol in dichloromethane to afford **10a** as a light yellow foam (163 mg, 88% yield). ^1^H NMR (400 MHz, CDCl_3_/TMS): δ 8.47 (m, 1H), 7.71–7.60 (m, 3H), 7.10 (m, 1H), 6.29 (br s, 2H), 5.73 (d, 1H, *J* = 6.0 Hz), 5.02 (m, 1H), 4.35–4.20 (m, 2H), 3.34 (m, 2H), 0.93 (s, 9H), 0.81 (s, 9H), 0.13 (s, 6H), −0.04 (s, 3H), −0.20 (s, 3H); ^13^C{1H} NMR (101 MHz, CDCl_3_): δ 159.5, 159.2, 153.5, 151.3, 149.8, 137.6, 137.2, 121.1, 120.2, 118.5, 89.6, 83.9, 74.9, 73.6, 42.1, 25.92, 25.83, 18.2, 18.0, −4.3, −4.4, −4.5, −4.9; HRMS (ESI/APCI) *m*/*z*: [M + H]^+^ calcd for C_27_H_45_N_6_O_4_Si_2_S_2_, 637.2477; found, 637.2489.

### 5′-Deoxy-2′,3′-*O*-di-(*tert*-butyldimethylsilyl)-5′-(5-nitropyridinyl-2-disulfanyl)guanosine (**10b**)

According to the procedure for the preparation of **10a**, disulfide **10b** (0.222 g, 70% yield) was prepared from **9** (258 mg, 0.466 mmol) and 2,2′-dithiobis(5-nitropyridine) (289 mg, 0.93 mmol) as a light yellow foam. ^1^H NMR (400 MHz, DMSO-*d*_3_/TMS): δ 10.6 (br s, 1H), 9.19 (d, 1H, *J* = 2.4 Hz), 8.49 (dd, 1H, *J* = 2.4, 8.8 Hz), 7.99 (*d*, 1H, *J* = 9.2 Hz), 7.94 (s, 1H), 6.44 (br s, 2H), 5.70 (d, 1H, *J* = 7.2 Hz), 4.99 (dd, 1H, *J* = 2.0, 7.2 Hz), 4.30–4.15 (m, 2H), 3.50–3.30 (m, 2H), 0.84 (s, 9H), 0.69 (s, 9H), 0.07 (s, 6H), −0.12 (s, 3H), −0.18 (s, 3H); ^13^C{^1^H} NMR (101 MHz, DMSO-*d*_3_): δ 167.5, 157.0, 153.8, 151.6, 145.0, 142.5, 137.1, 132.6, 119.9, 117.4, 86.9, 84.2, 74.5, 73.4, 41.2, 25.9, 25.7, 18.0, 17.8, −4.4, −4.5, −4.6, −5.3; HRMS (ESI/APCI) *m*/*z*: [M + H]^+^ calcd for C_27_H_44_N_7_O_6_Si_2_S_2_, 682.2328; found, 682.2320.

### 5′-Deoxy-2′,3′-*O*-di-(*tert*-butyldimethylsilyl)-*N*^2^-[(dimethylamino)methylene]-5′-(2-pyridinyldisulfanyl)guanosine (**8b**)

Under argon to a solution of **10a** (108 mg, 0.170 mmol) in methanol (10 mL), *N*,*N*-dimethylformamide dimethyl acetal (0.226 mL, 1.70 mmol) was added. The mixture was stirred at rt for overnight. The solvent was removed, and the residue was isolated by silica gel chromatography, eluting with 5% methanol in dichloromethane to afford **8b** as a light yellow foam: 61 mg (52% yield), eluting with 10% methanol in dichloromethane to recover starting material **10a** (40 mg, 37%). ^1^H NMR (CDCl_3_/TMS): δ 10.0 (br s, 1H), 8.52 (s, 1H), 8.46 (m, 1H), 7.71 (s, 1H), 7.62 (m, 1H), 7.59 (m, 1H), 7.10 (m, 1H), 5.83 (d, 1H, *J* = 5.6 Hz), 4.75 (dd, 1H, *J* = 4.4, 5.6 Hz), 4.32 (m, 1H), 4.20 (m, 1H), 3.31–3.27 (m, 2H), 3.18 (s, 3H), 3.13 (s, 3H), 0.91 (s, 9H), 0.80 (s, 9H), 0.11 (s, 3H), 0.10 (s, 3H), −0.06 (s, 3H), −0.22 (s, 3H); ^13^C NMR (CDCl_3_): δ 159.2, 158.4, 157.7, 156.8, 150.1, 149.8, 137.5, 137.1, 121.6, 121.1, 120.1, 88.8, 83.1, 74.7, 74.5, 42.4, 41.4, 35.5, 25.83, 25.75, 18.1, 18.0, −4.3, −4.5, −4.6, −5.0; HRMS (ESI/APCI) *m*/*z*: [M + H]^+^ calcd for C_30_H_50_N_7_O_4_Si_2_S_2_, 692.2899; found, 692.2907.

### 5′-Deoxy-2′,3′-*O*-di-(*tert*-butyldimethylsilyl)-*N*^2^-[(dimethylamino)methylene]-5′-(5-nitropyridinyl-2-disulfanyl)guanosine (**8c**)

From **10b**: Under argon to a solution of **10b** (150 mg, 0.220 mmol) in methanol (17 mL), *N*,*N*-dimethylformamide dimethyl acetal (0.294 mL, 2.20 mmol) was added. The mixture was stirred at rt overnight. The solvent was removed, and the residue was isolated by silica gel chromatography, eluting with 5% methanol in chloroform to afford **8c** as a light yellow foam: 81 mg (50% yield).

From **8b**: To a solution of **8b** (30 mg, 0.043 mmol) in CHCl_3_ (5 mL), DTT (17 mg, 0.11 mmol) was added, and the mixture was stirred at rt for 4 h. The solvent was removed, and the residue was isolated by silica gel chromatography, eluting with 5% methanol in dichloromethane. To the fraction containing 5′-deoxy-2′,3′-*O*-di-(*tert*-butyldimethylsilyl)-*N*^2^-[(dimethylamino)methylene]-5′-thioguanosine, 2,2′-dithiobis(5-nitropyridine) (37 mg, 0.12 mmol) (259 mg, 1.18 mmol) was added. The reaction mixture was stirred at rt for 48 h. The solvent was removed, and the residue was isolated by silica gel chromatography, eluting with 5% methanol in chloroform to afford **8c** as a light yellow foam (20 mg, 68% yield). ^1^H NMR (400 MHz, CDCl_3_/TMS): δ 9.53 (br s, 1H), 9.25 (d, 1H, *J* = 2.8 Hz), 8.53 (s, 1H), 8.36 (dd, 1H, *J* = 2.8, 8.8 Hz), 7.90 (dd, 1H, *J* = 0.4, 8.8 Hz), 7.71 (s, 1H), 5.83 (d, 1H, *J* = 4.0 Hz), 4.57 (m, 1H), 4.25 (m, 1H), 4.11 (t, 1H, *J* = 4.4 Hz), 3.35–3.18 (m, 2H), 3.20 (s, 3H), 3.15 (s, 3H), 0.89 (s, 9H), 0.81 (s, 9H), 0.092 (s, 3H), 0.086 (s, 3H), −0.02 (s, 3H), −0.12 (s, 3H); ^13^C{1H} NMR (101 MHz, CDCl_3_): δ 168.1, 158.2, 157.8, 156.9, 150.1, 145.2, 142.3, 136.9, 131.7, 121.2, 119.8, 88.9, 81.9, 75.3, 74.7, 42.3, 41.5, 35.4, 25.9, 25.8, 18.2, 18.0, −4.1, −4.5, −4.6; HRMS (ESI/APCI) *m*/*z*: [M + H]^+^ calcd for C_30_H_49_N_8_O_6_Si_2_S_2_, 737.2750; found, 737.2748.

### 5′-Deoxy-2′,3′-*O*-di-(*tert*-butyldimethylsilyl)-*N*^2^-[(dimethylamino)methylene]-5′-(*tert*-butyldisulfanyl)guanosine (**8d**)

To a solution of **8b** (20 mg, 0.029 mmol) in CHCl_3_ (2 mL), 2-methyl-2-propanethiol (32 μL, 0.29 mmol) and triethylamine (68 μL, 0.49 mmol) were added, and the mixture was stirred at rt for 2.5 h. The solvent was removed, and the residue was isolated by silica gel chromatography, eluting with 4% methanol in dichloromethane to afford **8d** as a colorless foam (17 mg, 87% yield). ^1^H NMR (400 MHz, CDCl_3_/TMS): δ 9.42 (br s, 1H), 8.57 (s, 1H), 7.74 (s, 1H), 5.87 (d, 1H, *J* = 5.6 Hz), 4.65 (dd, 1H, *J* = 4.4, 5.6 Hz), 4.31 (m, 1H), 4.25 (m, 1H), 3.19 (s, 3H), 3.14 (s, 3H), 3.18–3.03 (m, 2H), 1.35 (s, 9H), 0.95 (s, 9H), 0.82 (s, 9H), 0.15 (s, 3H), 0.13 (s, 3H), −0.05 (s, 3H), −0.22 (s, 3H); ^13^C{^1^H} NMR (101 MHz, CDCl_3_): δ 158.1, 157.9, 156.8, 150.3, 137.3, 121.5, 88.3, 83.9, 75.0, 74.1, 48.4, 43.9, 41.5, 35.3, 30.0, 26.0, 25.9, 18.2, 18.1, −4.27, −4.30, −4.5, −4.9; HRMS (ESI/APCI) *m*/*z*: [M + H]^+^ calcd for C_29_H_55_N_6_O_4_Si_2_S_2_, 671.3259; found, 671.3259.

### Dinucleotide 5′-C_2′-NHX_-ps-G-3′ (**11**)

#### Synthesis by the Coupling of **5** to **8a**

Under argon, *N*,*O*-bis(trimethylsilyl)trifluoroacetamide (0.223 mL, 0.84 mmol) was added to a solution of **5** (30 mg, 0.028 mmol) and **8a** (17 mg, 0.031 mmol) in anhydrous THF (5.0 mL). The mixture was stirred under reflux for 1.5 h. The solvent was removed, and the residue was treated with 50% formic acid (4.0 mL) at rt for 48 h. After evaporation of the mixture under vacuum, the remaining residue was treated with a mixture of NH_4_OH/MeOH (3:1, v/v) (8.0 mL) at 55 °C in a sealed vial in an oven for 4 h. After cooling the mixture, the solvent was removed, and the residue was dissolved in water (10 mL) and washed with chloroform (3 × 5 mL). The aqueous phase was evaporated, and the residue was purified by silica gel chromatography, eluting with acetonitrile/water/triethylamine (90:10:1 v/v/v) to afford the desired dinucleotide **11** as a colorless foam (20 mg, 76% yield).

#### Synthesis by the Coupling of **5** to **8c**

Under argon, *N*,*O*-bis(trimethylsilyl)trifluoroacetamide (0.135 mL, 0.51 mmol) was added to a solution of **5** (18 mg, 0.017 mmol) and **8c** (19 mg, 0.026 mmol) in anhydrous THF (5.0 mL). The mixture was stirred under reflux for 1.5 h. The solvent was removed, and the residue was treated with 80% acetic acid (5.0 mL) at rt for 10 min. After evaporation of the mixture under vacuum, the remaining residue was treated with a mixture of NH_4_OH/EtOH (3:1, v/v) (8.0 mL) at 55 °C in a sealed vial in an oven for 4 h. After cooling the mixture, the solvent was removed, and the residue was treated with 0.6 mL of NMP/Et_3_N/Et_3_N–3HF solution (by mixing 275 μL of NMP and 140 μL of triethylamine with 180 μL Et_3_N–3HF) at 65 °C in a water bath for 1 h. The reaction mixture was diluted with water (1 mL) and washed with chloroform (3 × 0.2 mL). The aqueous phase was evaporated, and the residue was purified by silica gel chromatography, eluting with acetonitrile/water/triethylamine (90:10:1 v/v/v) to afford the desired dinucleotide **11** as a colorless film (12 mg, 75% yield). The purity of **11** is estimated to be ∼90% by reverse-phase HPLC using a C18 column (HPLC conditions: Thermo Scientific Acclaim C18, 5 μm 120 Å 4.6 × 250 mm column; flow rate: 1.0 mL/min; buffer A, 0.1 M TEAA, pH 7; B, acetonitrile; 0–5 min, 100% A, 0% B; 5–35 min, 70% A, 30% B; 35–37 min, 0% A, 100% B; 37–41 min 0% A, 100% B; 41–43 min, 100% A, 0% B) with retention time 25.6 min. ^1^H NMR (400 MHz, D_2_O): δ 7.93 (d, 1H, *J* = 7.4 Hz), 7.80 (s, 1H), 7.66 (s, 1H), 6.32 (s, 1H), 6.12 (d, 1H, *J* = 7.5 Hz), 6.09 (d, 1H, *J* = 9.3 Hz), 5.69 (s, 1H, *J* = 5.6 Hz); ^31^P NMR (162 MHz, D_2_O): δ 22.4; HRMS (ESI/APCI) *m*/*z*: [M + H]^+^ calcd for C_29_H_34_N_10_O_16_PS, 841.1613; found, 841.1606.

### Trinucleotide 5′-C_2′-NHX_-ps-GG-3′ (**13**)

#### Synthesis via Solid-Phase Synthesis

The synthesis was started by using an Expedite 8909 synthesizer via a modified 1 μmol RNA protocol (trityl on). After standard detritylation, the 1 μmol *i*-Pr-Pac-G-RNA-CPG column was double coupling to 5′-tritylthioguanosine phosphoramidite (**12**)^34^ (68 mg, 0.075 mmol) in dry acetonitrile (0.75 mL) and followed by standard capping and oxidation. The CPG column was then removed from the synthesizer and treated with the solution of AgNO_3_ (26 mg) in water (3 mL) at rt for 1 h. The CPG column was washed with water (10 mL) and further treated with the solution of DTT (23 mg) in water (3 mL) at rt for 30 min. The CPG column was subsequently rinsed with water (5 mL), acetonitrile (5 mL), and CH_2_Cl_2_ (5 mL). The CPG column was then treated with the solution of 2,2′-dithiobis(5-nitropyridine) (46.5 mg, 0.150 mmol) in dry DMF (3 mL) at rt overnight. The CPG column was rinsed with acetonitrile (5 mL) and CH_2_Cl_2_ (5 mL) and dried under vacuum for 30 min. Under argon, *N*,*O*-bis(trimethylsilyl)trifluoroacetamide (80 μL, 0.30 mmol) was added to a dried 10 mL flask containing the dried CPG and the 2′-photocaged amino 3′-*H*-phosphonate (**5**) (10 mg, 10 μmol) in anhydrous THF (3 mL). The mixture was stirred under argon at reflux for 1 h. The solvent was removed, and the solid supports were treated with 3% trichloroacetic acid (2 mL) in CH_2_Cl_2_ at rt for 5 min. After rinsing with CH_2_Cl_2_ (5 mL), the supports were treated with a mixture of concentrated ammonium hydroxide/ethanol (3:1, v/v) (2 mL) at rt overnight and then at 55 °C for 1 h in a sealed tube in an oven. After cooling down in ice, the supernatant solution was removed, and the support was rinsed with an ethanol/acetonitrile/water (3:1:1) mixture. The solutions were combined and evaporated to dryness. The residue was desilylated with a mixture of NMP/Et_3_N/Et_3_N–3HF (300 μL) (6:3:4, v/v/v) at 65 °C in a water bath for 25 min. The solvent was removed at rt under vacuum. The residue was extracted into water (1.0 mL) and washed with chloroform (3 × 0.3 mL). The aqueous phase was desalted by a C18 Sep-Pak column. The product was then purified by a reverse-phase HPLC column to afford the desired trinucleotide **13** as a colorless foam (50 nmol, 5% yield). MALDI-TOF mass *m*/*z*: [M + H]^+^ calcd for C_39_H_48_N_15_O_23_P_2_S, 1188.22; found, 1188.21.

### 5′-*O*-Dimethoxytrityl-2′,3′-*O*-di-(*tert*-butyldimethylsilyl)-*N*^2^-phenoxyacetylguanosine (**15**)

5′-*O*-Dimethoxytrityl-*N*^2^-phenoxyacetylguanosine (**14**) (0.500 g, 0.695 mmol) was co-evaporated with toluene (2 × 10 mL), dried under vacuum, and then dissolved into DMF (10 mL). To the resulting solution, imidazole (1.62 g, 23.9 mmol) was added, followed by TBSCl (746 mg, 5.00 mmol). The mixture was stirred under argon at rt overnight. The solvent was removed, and the residue was dissolved into dichloromethane (30 mL). The dichloromethane solution was washed with saturated aqueous NaHCO_3_ and brine. The solvent was removed, and the residue was purified by silica gel chromatography, eluting with 2% methanol in chloroform to afford **15** as a white foam: 0.519 g (79% yield). ^1^H NMR (400 MHz, CDCl_3_/TMS): δ 11.78 (br s, 1H), 8.89 (br s, 1H), 8.00 (s, 1H), 7.52–7.46 (m, 2H), 7.41–7.20 (m, 9H), 7.11 (t, 1H, *J* = 7.2 Hz), 6.91–6.82 (m, 6H), 5.92 (d, 1H, *J* = 4.8 Hz), 4.63 (s, 2H), 4.50 (t, 1H, *J* = 4.6 Hz), 4.22 (dd, 1H, *J* = 4.0, 6.8 Hz), 4.14 (t, 1H, *J* = 4.4 Hz), 3.79 (s, 6H), 3.50 (dd, 1H, *J* = 2.8, 10.8 Hz), 3.30 (dd, 1H, *J* = 4.4, 10.8 Hz), 0.85 (s, 9H), 0.84 (s, 9H), 0.03 (s, 3H), 0.02 (s, 3H), −0.09 (s, 3H), −1.05 (s, 3H); ^13^C{^1^H} NMR (101 MHz, CDCl_3_): δ 169.5, 158.8, 156.4, 155.5, 147.8, 146.1, 144.5, 137.6, 135.62, 135.59, 130.18, 130.16, 130.1, 128.2, 128.1, 127.2, 88.3, 86.9, 84.5, 72.3, 67.0, 63.4, 55.4, 25.9, 25.8, 18.1, 18.0, −4.2, −4.4, −4.7, −4.8; HRMS (ESI/APCI) *m*/*z*: [M + H]^+^ calcd for C_51_H_66_N_5_O_9_Si_2_, 948.4394; found, 948.4407.

### 2′,3′-*O*-Di-(*tert*-butyldimethylsilyl)-*N*^2^-phenoxyacetylguanosine (**16**)

Compound 15 (0.438 g, 0.462 mmol) was treated with 3% trichloroacetic acid in dichloromethane (10 mL) at rt for 5 min. The mixture was diluted with dichloromethane (20 mL). The solution was washed with saturated aqueous NaHCO_3_ and brine. The solvent was removed, and the residue was purified by silica gel chromatography, eluting with 5% methanol in chloroform to afford 16 as a white foam: 0.280 g, (94% yield). ^1^H NMR (500 MHz, CDCl_3_/TMS): δ 11.76 (br s, 1H), 9.27 (br s, 1H), 7.80 (s, 1H), 7.38 (t, 2H, *J* = 7.5 Hz), 7.10 (t, 1H, *J* = 7.5 Hz), 7.03 (d, 2H, *J* = 7.5 Hz), 5.74 (d, 1H, *J* = 7.5 Hz), 5.42 (d, *J* = 10.5 Hz), 4.74–4.65 (m, 3H), 4.31 (d, 1H, *J* = 4.5 Hz), 4.17 (s, 1H), 3.97 (dd, 1H, *J* = 1.2, 10.0 Hz), 3.77 (t, 1H, *J* = 11.0 Hz), 0.95 (s, 9H), 0.79 (s, 9H), 0.13 (s, 3H), 0.12 (s, 3H), −0.07 (s, 3H), −0.44 (s, 3H); ^13^C{^1^H} NMR (126 MHz, CDCl_3_): δ 169.5, 156.2, 154.9, 146.4, 146.1, 139.6, 130.0, 123.4, 123.0, 114.8, 90.7, 88.7, 74.7, 73.5, 66.5, 62.7, 25.8, 25.6, 18.0, 17.8, −4.54, −4.56, −4.64, −5.7; HRMS (ESI/APCI) *m*/*z*: [M + H]^+^ calcd for C_30_H_48_N_5_O_7_Si_2_, 646.3087; found, 646.3094.

### Trinucleotide 5′-C_2′-NHX_-ps-GG-3′ (**13**)

#### Synthesis via Solution Method

Under argon to a solution of guanosine derivative **16** (71 mg, 0.11 mmol) and 5′-disulfide phosphoramidite **17**(34) (91 mg, 0.11 mmol) in CH_3_CN (1.0 mL), the standard activator solution (0.45 tetrazole in acetonitrile, 1.0 mL) was added. After stirring the mixture at rt for 30 min, it was oxidized with 10% *tert*-butyl hydroperoxide (1.0 mL) at rt for 10 min. The solvent was removed, and the residue was isolated by silica gel chromatography, eluting with 5% methanol in chloroform to afford the protected dinucleotide derivative **18**: 54 mg (37% yield). ^31^P{1H} NMR (162 MHz, CD_3_CN): δ −1.33, −1.57; MALDI-TOF mass *m*/*z*: [M + Na]^+^ calcd for C_56_H_89_N_12_NaO_13_PS_2_Si_3_, 1339.51; found, 1339.55. Under argon, to a solution of **18** (16 mg, 0.012 mmol) in chloroform (2.0 mL), DTT (23 mg, 0.15 mmol) and Et_3_N (50 μL, 0.36 mmol) were added, and the mixture was stirred at rt for 24 h. The solvent was removed, and the residue was dissolved into CH_2_Cl_2_. The solution was subsequently washed with saturated NaHCO_3_, water, and brine and dried over anhydrous MgSO_4_. The solvent was removed, and the residue was treated with the solution of 2,2′-dithiobis(5-nitropyridine) (11 mg, 36 μmol) in dry DMF (2 mL) at rt overnight. The solvent was removed, and the residue was purified by silica gel chromatography, eluting with 5% MeOH in CH_2_Cl_2_ containing 5% Et_3_N to afford a mixture of 5′-disulfide derivatives (confirmed by MS). Under argon, to the mixture of the above-prepared 5′-disulfide derivatives in THF (10 mL), *N*,*O*-bis(trimethylsilyl)trifluoroacetamide (319 μL, 1.20 mmol) and the 2′-photocaged amino 3′-*H*-phosphonate (**5**) (13 mg, 12 μmol) were added. The mixture was stirred under argon at reflux for 1 h. The solvent was removed, and the residue was treated with 80% AcOH (3 mL) in CH_2_Cl_2_ at rt for 30 min. The solvent was removed, and the residue was dried under vacuum and then treated with saturated ammonia in methanol in a 4 °C refrigerator overnight. The solvent was removed, and the residue was desilylated with a mixture of NMP (450 μL), Et_3_N (225 μL), and Et_3_N–3HF (300 μL) at 65 °C in a water bath for 25 min. The solvent was removed at rt under vacuum. The residue was dissolved into water (1.5 mL) and washed with chloroform (4 × 1.0 mL). The aqueous phase was desalted via a C18 Sep-Pak column. The product was then purified by reverse-phase HPLC column to afford the desired trinucleotide **13** as a colorless film (252 nmol, 2.1% yield). MALDI-TOF mass *m*/*z*: [M + H]^+^ calcd for C_39_H_48_N_15_O_23_P_2_S, 1188.22; found, 1188.37.

### *N*^2^-Isobutyryl-2′-*O*-(*o*-nitrobenzyl)-5′-*O*-(*p*-toluenesulfonyl)guanosine (**20**)

Compound **19**(35) (723 mg, 1.48 mmol) was dried by co-evaporation with dry pyridine (2 × 5 mL) under vacuum. Under argon, to the solution of dried **19** in dry pyridine (10 mL), TsCl (423 mg, 2.22 mmol) was added, and the mixture was stirred at rt for 40 h. The reaction was quenched by the addition of methanol (1.0 mL). After 10 min, the solvent was removed, and the residue was isolated by silica gel chromatography, eluting with 1.5–3% methanol in dichloromethane to afford **20** as a white foam: 526 mg (55% yield). ^1^H NMR (400 MHz, CDCl_3_/TMS): δ 12.24 (s, 1H), 10.08 (s, 1H), 7.87–7.81 (m, 2H), 7.73 (d, 2H, *J* = 8.4 Hz), 7.56 (d, 1H, *J* = 8.0 Hz), 7.44 (t, 1H, *J* = 7.6 Hz), 7.35–7.25 (m, 3H), 5.99 (d, 1H, *J* = 5.2 Hz), 5.09 (d, 1H, *J* = 14.4 Hz), 5.05 (br s, 1H), 4.99 (d, 1H, *J* = 14.4 Hz), 4.80–4.65 (m, 2H), 4.40–4.20 (m, 3H), 2.86 (m, 1H), 2.41 (s, 3H), 1.27 (d, 3H, *J* = 6.8 Hz), 1.26 (d, 3H, *J* = 6.8 Hz); ^13^C{^1^H} NMR (101 MHz, CDCl_3_): δ 180.1, 155.7, 148.2, 147.9, 147.4, 145.6, 139.2, 133.6, 133.5, 131.9, 130.1, 129.3, 128.5, 127.9, 124.4, 121.7, 87.9, 82.5, 80.7, 69.6, 69.3, 69.2, 36.2, 21.7, 19.02, 18.99; HRMS (ESI/APCI) *m*/*z*: [M + H]^+^ calcd for C_28_H_31_N_6_O_10_S, 643.1817; found, 643.1822.

### 5′-Acetylthio-5′-deoxy-*N*^2^-isobutyryl-2′-*O*-(*o*-nitrobenzyl)-guanosine (**21a**) and 3′-*O*, 5′-*S*-Diacetyl-5′-deoxy-*N*^2^-isobutyryl-2′-*O*-(*o*-nitrobenzyl)-5′-thioguanosine (**21b**)

Under argon, to the solution of **20** (676 mg, 1.05 mmol) in DMF (10 mL), potassium thioacetate (240 mg, 2.10 mmol) was added. The mixture was stirred at 60 °C in an oil bath for 17 h. The solvent was removed, and the residue was isolated by silica gel chromatography, eluting with 1–3% methanol in dichloromethane to afford **21a**: 365 mg (63% yield, lower spot on TLC) and **21b**: 127 mg (21% yield, higher spot on TLC) as light yellow foams. **21a**: ^1^H NMR (400 MHz, CDCl_3_/TMS): δ 12.29 (s, 1H), 10.11 (s, 1H), 7.94 (dd, 1H, *J* = 8.4, 1.2 Hz), 7.92 (s, 1H), 7.70 (d, 1H, *J* = 8.0 Hz), 7.55 (m, 1H), 7.39 (m, 1H), 5.97 (d, 1H, *J* = 2.0 Hz), 5.20 (d, 1H, *J* = 14.8 Hz), 5.14 (d, 1H, *J* = 14.4 Hz), 4.59 (br s, 1H), 4.53 (m, 2H), 4.25 (m, 1H), 3.52 (dd, 1H, *J* = 14.0, 5.2 Hz), 3.26 (dd, 1H, *J* = 14.0, 6.8 Hz), 2.90 (m, 1H), 2.39 (s, 3H), 1.27 (d, 3H, *J* = 6.8 Hz), 1.25 (d, 3H, *J* = 6.8 Hz); ^13^C{^1^H} NMR (101 MHz, CDCl_3_): δ 195.9, 180.0, 155.7, 148.1, 147.9, 147.1, 138.1, 134.0, 133.9, 129.1, 128.5, 124.6, 121.6, 88.2, 82.6, 82.4, 71.9, 69.5, 36.3, 30.9, 30.7, 19.1, 19.0; HRMS (ESI/APCI) *m*/*z*: [M + H]^+^ calcd for C_23_H_27_N_6_O_8_S, 547.1606; found, 547.1595. **21b**: ^1^H NMR (500 MHz, CDCl_3_/TMS): δ 12.17 (s, 1H), 9.73 (s, 1H), 7.94 (d, 1H, *J* = 6.4 Hz), 7.85 (s, 1H), 7.55–7.50 (m, 2H), 7.40 (m, 1H), 5.91 (d, 1H, *J* = 3.6 Hz), 5.35 (t, 1H, *J* = 4.0 Hz), 5.16 (d, 1H, *J* = 12.0 Hz), 5.02 (t, 1H, *J* = 4.0 Hz), 4.95 (d, 1H, *J* = 12.0 Hz), 4.41 (m, 1H), 3.64 (dd, 1H, *J* = 11.2, 5.2 Hz), 3.33 (dd, 1H, *J* = 11.2, 4.8 Hz), 2.82 (m, 1H), 2.40 (s, 3H), 2.15 (s, 3H), 1.30 (d, 3H, *J* = 6.8 Hz), 1.28 (d, 3H, *J* = 6.8 Hz); ^13^C{^1^H} NMR (126 MHz, CDCl_3_): δ 196.0, 179.4, 170.1, 155.6, 147.8, 147.6, 147.4, 138.2, 133.83, 133.82, 128.8, 128.6, 124.6, 122.4, 88.4, 80.6, 79.3, 72.4, 69.5, 36.4, 30.8, 30.5, 20.8, 19.1, 19.0; HRMS (ESI/APCI) *m*/*z*: [M + H]^+^ calcd for C_25_H_29_N_6_O_9_S, 589.1711; found, 589.1714.

### 5′-Deoxy-*N*^2^-isobutyryl-2′-*O*-(*o*-nitrobenzyl)-5′-(5-nitropyridinyl-2-disulfanyl)guanosine (**22**)

From **21a**: Under argon, to the mixture of **21a** (300 mg, 0.55 mmol) and 2,2′-dithiobis(5-nitropyridine) (341 mg, 1.10 mmol) in anhydrous dichloromethane (20 mL) at 0 °C, the solution of guanidine hydrocholoride/guanidine (4:1) in methanol (10 mL) prepared from sodium methoxide (0.50 M solution in CH_3_OH, 1.2 mL, 0.60 mmol) and guanidine hydrochloride (268 mg, 2.80 mmol) in methanol (9.0 mL) was added. After stirring the reaction mixture at rt for 3 h, TLC showed that the reaction was not complete. Additional sodium methoxide (0.50 M solution in CH_3_OH, 1.2 mL, 0.60 mmol) was added, and the mixture was stirred at rt for an additional 5 h. The reaction mixture was neutralized with 1 N HCl. The solvent was removed, and the residue was isolated by silica gel chromatography, eluting with 0–3% methanol in dichloromethane to afford **22** as a light yellow foam: 147 mg (41% yield).

From **21b**: Under argon, to the mixture of **21b** (187 mg, 0.32 mmol), 2,2′-dithiobis(5-nitropyridine) (199 mg, 0.64 mmol), and guanidine hydrocholoride (366 mg, 3.83 mmol) in a mixed solvent of anhydrous dichloromethane/methanol (20 mL, v/v = 1:1) at 0 °C, sodium methoxide (0.50 M solution in CH_3_OH, 1.36 mL, 0.68 mmol) was added, and the mixture was stirred at rt for 3.5 h. The reaction mixture was neutralized with 1 N HCl. The solvent was removed, and the residue was isolated by silica gel chromatography, eluting with 2% methanol in dichloromethane to afford **22** as a light yellow foam: 133 mg (63% yield).

^1^H NMR (400 MHz, CDCl_3_/TMS): δ 12.33 (br s, 1H), 9.97 (br s, 1H), 9.15 (s, 1H), 8.32 (d, 1H, *J* = 8.8 Hz), 8.00–7.85 (m, 3H), 7.71 (d, 1H, *J* = 7.6 Hz), 7.57 (t, 1H, *J* = 7.6 Hz), 7.39 (m, 1H), 5.95 (s, 1H), 5.24 (d, 1H, *J* = 14.8 Hz), 5.15 (d, 1H, *J* = 12.0 Hz), 4.82 (br s, 1H), 4.72 (br s, 1H), 4.50 (m, 1H), 4.31 (m, 1H), 3.40 (m, 1H), 3.27 (m, 1H), 2.87 (m, 1H), 1.30 (d, 3H, *J* = 6.8 Hz), 1.28 (d, 3H, *J* = 6.8 Hz); ^13^C{^1^H} NMR (101 MHz, CDCl_3_): δ 180.1, 168.5, 155.8, 148.2, 148.0, 147.1, 145.0, 142.1, 138.0, 134.2, 134.0, 131.8, 129.1, 128.6, 124.8, 121.5, 119.7, 88.4, 82.7, 81.9, 72.3, 69.7, 41.9, 36.4, 19.1, 19.0; HRMS (ESI/APCI) *m*/*z*: [M + H]^+^ calcd for C_26_H_27_N_8_O_9_S_2_, 659.1337; found, 659.1331.

### 5′-Deoxy-*N*^2^-isobutyryl-2′-*O*-(*o*-nitrobenzyl)-5′-(5-nitropyridinyl-2-disulfanyl)guanosine 3′-*O*-Phosphonate (**23**)

2-Chlorophenyl phosphorodichloridate (140 mg, 0.58 mmol) was added to a magnetically stirred solution of 1,2,4-triazole (88 mg, 1.3 mmol) and dry Et_3_N (0.16 mL, 1.2 mmol) in dry THF (5.0 mL), after 15 min at rt, **22** (76.0 mg, 0.115 mmol) in THF (4.0 mL) and 1-methylimidazole (74 μL, 0.92 mmol) were added. After 60 min at rt, the resulting mixture was quenched by adding distilled water (29 μL) and Et_3_N (0.16 mL, 1.2 mmol). The solvent was removed, and the recovered crude yellow oil was partitioned between saturated aqueous NaHCO_3_ and dichloromethane. The organic layer was washed with brine and dried over MgSO_4_. The solution was evaporated, and the residue was purified by silica gel chromatography, eluting with 2% methanol in dichloromethane containing 2% Et_3_N to afford **23** as a brown solid: 104 mg (95% yield). ^1^H NMR (500 MHz, CDCl_3_/TMS): δ 11.08 (br s, 1H), 9.16 (d, 1H, *J* = 2.5 Hz), 8.31 (dd, 1H, *J* = 9.0, 2.5 Hz), 7.95 (d, 1H, *J* = 9.0 Hz), 7.89 (d, 1H, *J* = 8.0 Hz), 7.76 (s, 1H), 7.69 (d, 1H, *J* = 7.5 Hz), 7.57 (d, 1H, *J* = 8.0 Hz), 7.51 (t, 1H, *J* = 7.5 Hz), 7.38 (t, 1H, *J* = 7.8 Hz), 7.22 (d, 1H, *J* = 7.5 Hz), 6.95 (m, 1H), 6.85 (m, 1H), 5.88 (m, 1H), 5.84 (d, 1H, *J* = 2.5 Hz), 5.16 (m, 1H), 5.06 (dd, 1H, *J* = 23, 14.5 Hz), 3.50–3.30 (m, 3H), 2.90 (m, 1H); ^13^C NMR (126 MHz, CDCl_3_): δ 180.1, 169.2, 155.9, 149.1, 148.0, 147.7, 147.3, 144.7, 141.9, 138.5, 134.1, 133.4, 131.7, 130.02, 129.99, 129.3, 128.3, 127.5, 124.4, 123.8, 121.8, 121.0, 119.4, 88.1, 80.5, 80.1, 76.2, 69.7, 42.1, 35.9, 19.02, 18.98; ^31^P NMR (202 MHz, CDCl_3_): δ −6.42; HRMS (ESI/APCI) *m*/*z*: [M – H]^−^ calcd for C_32_H_29_N_8_O_12_PS_2_Cl, 847.0778; found, 847.0776.

### Synthesis of 5′-C_2′-NHX_-ps-G_2′-*o*-NBn_GGUCGGC-3′ (**24a**) and 5′-UUC_2′-NHX_-ps-G_2′-*o*-NBn_GGUCGGC-3′ (**24b**)

The 3′-end of these RNAs: 5′-HO-GGUCGGC (1.0 μmol scale) on a solid support was synthesized by the standard solid-phase synthesis and dried under vacuum. Under argon, a solution of 2′-*O*-photocaged 5′-disulfide guanosine **23** (50 mg, 53 μmol) in dry pyridine (0.35 mL) was injected into the flask containing MSNT (30 mg, 0.10 mmol). The flask was agitated gently to encourage dissolution of MSNT. 1-Methylimidazole (14 μL, 175 μmol) was then injected into the mixture. After 1 min, the solution was injected into the column containing 5′-HO-GGUCGGC on a solid support and the column, which was attached to two 1 mL syringes, was allowed to stand for 30 min. The support was subsequently washed with pyridine (1.0 mL) and dichloromethane (5.0 mL), and then dried under vacuum. The solid support was poured into the flask containing 2′-photocaged aminocytidine 3′-*H*-phosphonate **5** (10.5 mg, 10 μmol, 10 equiv). Under argon, THF (2.0 mL) and *N*,*O*-bistrimethylsilyl trifluoroacetamide (0.266 mL, 1.0 mmol) were added, and the mixture was stirred at reflux for 2 h. The support was collected by filtration with an empty column, rinsed with dichloromethane, and dried under vacuum for 30 min. The column was put back to the synthesizer. It can be deprotected at this stage to prepare **24a** or continued for the rest of the synthesis to **24b**. After the final DMTr removal, the support was transferred to a small vial. Cleavage was performed with a solution of pyridine-2-carboxaldoxime/tetramethylguanidine (0.10 M) in dioxane/water (2:1, 0.5 mL) for 19.5 h. The solvent was evaporated and the residue was treated with NH_4_OH/CH_3_CH_2_OH (3:1, v/v) at rt for 22 h and then at 55 °C in an oven for 6 h. The solution was transferred to a 1.7 mL vial. Followed by evaporation, desilylation (65 °C, 1.5 h) in a water bath and ethanol precipitation, the pellets were dissolved into TE (400 μL). The modified oligonucleotides: **24a** (16.1 nmol, 1.61% yield) and **24b** (8.6 nmol, 0.86% yield) were obtained by ion exchange column HPLC purification (HPLC conditions: Dionex DNAPac PA-100 column, 9 × 250 mm; flow rate: 2.0 mL/min; buffer A, 0.25 M tris, pH 8.93; B, water; C, 1.0 M NaCl; 0.0 min, 10% A, 60% B, 30% C; 10 min, 10% A, 60% B, 30% C; 40 min, 10% A, 30% B, 60% C; 42 min, 10% A, 0% B, 90% C) and then desalted by Sep-Pack C18. **24a**: retention time 32.9 min; calcd for [MH^+^], 3274.5; found, 3275.0. **24b**: retention time 36.2 min; calcd for [MH^+^], 3886.6; found, 3887.3.

### Synthesis of 5′-C_2′-NHX_G_2′-*o*-NBn_GGUCGGC-3′ (**26a**) and 5′-UUC_2′-NHX_G_2′-*o*-NBn_GGUCGGC-3′ (**26b**)

The 3′-end of these RNAs: 5′-HO-GGUCGGC (1.0 μmol scale) on a solid support was synthesized by the standard solid-phase synthesis. The protocol was then modified for double coupling to 2′-*O*-photocaged guanosine phosphoroamidite **25**(42) (70 mg) in dry CH_3_CN (0.75 mL). After standard capping, oxidation, and detritylation, half of the oligonucleotide on the sold support (∼0.5 μmol) was manually coupled to the 2′-photocaged aminocytidine phosphoamidite **4a** with one syringe containing the solution of **4a** (83 mg) in dry CH_3_CN (0.5 mL) and another syringe containing the activator (0.3 mL, 0.45 M tetrazole in CH_3_CN). The coupling time of **4a** was at rt for 30 min. After standard capping, oxidation, and detritylation, half of the support (∼0.25 μmol) was deprotected to prepare **26a**. The second half of the support (∼0.25 μmol) was put back to the synthesizer and continued for the rest of the synthesis to prepare **26b**. After the final DMTr removal, the support was transferred to a small vial and treated with NH_4_OH/ethanol (3:1, v/v) at rt overnight and then at 55 °C in oven for 6 h. Followed by evaporation, desilylation (65 °C, 1.5 h) in a water bath, and ethanol precipitation, the pellets were dissolved into TE (400 μL). The modified oligonucleotides, **26a** (17.6 nmol, 7.04% yield) and **26b** (12.2 nmol, 4.9% yield), were obtained by ion exchange column HPLC purification (HPLC conditions are the same as above for the purification of **24a** and **24b**) and desalted by Sep-Pack C18. **26a**: HPLC retention time 29.7 min; MALDI-TOF MS calcd for [MNa^+^], 3280.5; found, 3280.4. **26b**: HPLC retention time 34.7 min; calcd for [MNa^+^], 3892.6; found, 3892.8.

### 3′-Radiolabeling of Oligonucleotides **24b** and **26b**

**24b** (20 μM, 1.0 μL) or **26b** (20 μM, 1.0 μL), ATP (100 μM, 0.6 μL), 10X ligase buffer (1.0 μL), DTT (100 mM, 0.33 μL), DMSO (1.0 μL), T4 RNA ligase (20 U, 1 μL), and 5′-[^32^P]-pCp (3000 ci/mmol, 10 mci/mL, 6 μL, 20 pmol) in a single RNase-free microfuge tube were incubated at 5 °C overnight. The 3′-radiolabeled oligonucleotides **24b** and **26b** were purified by 20% denatural polyacrylamide *gel* electrophoresis (dPAGE). 10× ligase buffer: 500 mM Tris-HCl, pH 7.78, 100 mM MgCl_2_, 100 mM DTT, 10 mM ATP.

### Characterization of **24b** and **26b**

(i) Silver ion cleavage: 4K cpm of the 3′-radiolabeled oligonucleotide **24b** (4 μL) or **26b** was treated with AgNO_3_ (100 mM, 0.4 μL) in a total volume of 20 μL solution in the dark at rt for 60 min. DTT (100 mM, 0.6 μL) was then added, and the mixture was spun for 3 min. A 15 μL aliquot of solution was withdrawn, added to quenching solution (15 μL), and run on a 20% dPAGE. (ii) Hydrolysis ladder: 2K cpm of the 3′-radiolabeled oligonucleotide **24b** (2 μL) or **26b** was treated with NaHCO_3_ (50 mM, pH 9, 2 μL) in a total volume of 10 μL solution at 90 °C on a heating block for 15 min. The mixture was chilled on ice and added to a quenching solution (8 μL) and run on a 20% dPAGE. Quenching solution: 0.01% bb/xc in 90% formamide, 10 mM EDTA, 2 mM tris, pH 7.

### Cleavage of **24b** in the Presence and Absence of HDV Ribozyme

Following the previously described protocol for HDV ribozyme-catalyzed substrate cleavage,^12,41^ we investigated the cleavage reaction of 5′-radiolabeled **24b** (∼1 nM) in the presence and absence of anti-genomic HDV ribozyme^12,41^ (1 μM) and 10 mM MgCl_2_. The yield of photodeprotection was about 30%, and the ribozyme kinetics were evaluated based on the reacted materials.^48^

## Supporting Information Available

^1^H NMR and ^31^P NMR of phosphoramidites **4a** and **4b**; dinucleotide **11**; and a dinucleotide intermediate **18**; MALDI-TOF MS of **13**, **18**, **24a**, **24ba**, **26a**, and **26b**; and ^1^H NMR and ^13^C NMR spectra of all the other new compounds (PDF)
