# Supplementary material for: Synthesis of Oligoribonucleotides Containing a 2′-Amino-5′-S-phosphorothiolate Linkage
Source: J Org Chem. 2021 Sep 17;86(19):13231–44. doi: 10.1021/acs.joc.1c01059 (PMC8491167; doi:10.1021/acs.joc.1c01059)
Supplement: Supplementary file 1 — jo1c01059_si_001.pdf [file jo1c01059_si_001.pdf]

Synthesis of oligoribonucleotides containing a 2′-amino-5′-*S*-phosphorothiolate linkage

Nan-Sheng Li,[1,*] Selene C. Koo,[1] and Joseph A. Piccirilli[1,2,*]

[1]*Department of Biochemistry & Molecular Biology and* [2]*Department of Chemistry, University of Chicago, 929 East 57th Street, Chicago, Illinois 60637*

nli@uchicago.edu, jpicciri@uchicago.edu

Content Page#

^{1}H NMR of **2** S4
^{13}C{^{1}H} NMR of **2** S5
^{1}H NMR of **3** S6
^{13}C{^{1}H} NMR of **3** S7
^{1}H NMR of **4a** S8
^{31}P{^{1}H} NMR of **4a** S9
^{1}H NMR of **4b** S10
^{31}P{^{1}H} NMR of **4b** S11
^{1}H NMR of **5** S12
^{13}C{^{1}H} NMR of **5** S13
^{13}P{^{1}H} NMR of **5** S14
^{1}H NMR of **8a** S15
^{13}C{^{1}H} NMR of **8a** S16
^{1}H NMR of **8b** S17
^{13}C{^{1}H} NMR of **8b** S18
^{1}H NMR of **8c** S19
^{13}C{^{1}H} NMR of **8c** S20
^{1}H NMR of **8d** S21
^{13}C{^{1}H} NMR of **8d** S22
^{1}H NMR of **9** S23
^{13}C{^{1}H} NMR of **9** S24

^{1}H NMR of **10a** ………………………………………………………………………... S25
$^{13}C\{^{1}H\}$ NMR of **10a** ……………………………………………………………….. S26
^{1}H NMR of **10b** ………………………………………………………………………... S27
$^{13}C\{^{1}H\}$ NMR of **10b** ……………………………………………………………….. S28
^{1}H NMR of **15** ………………………………………………………………………… S29
$^{13}C\{^{1}H\}$ NMR of **15** ………………………………………………………………… S30
^{1}H NMR of **16** ………………………………………………………………………… S31
$^{13}C\{^{1}H\}$ NMR of **16** ………………………………………………………………… S32
^{1}H NMR of **18** ………………………………………………………………………… S33
$^{31}P\{^{1}H\}$ NMR of **18** ……………………………………………………………….... S34
MALDI-TOF MS of **18** …………………………………………………………….. S35
^{1}H NMR of **20** ………………………………………………………………………… S36
$^{13}C\{^{1}H\}$ NMR of **20** ………………………………………………………………… S37
^{1}H NMR of **21a** ………………………………………………………………………... S38
$^{13}C\{^{1}H\}$ NMR of **21a** ……………………………………………………………….. S39
^{1}H NMR of **21b** ………………………………………………………………………... S40
$^{13}C\{^{1}H\}$ NMR of **21b** ………………………………………………………………… S41
^{1}H NMR of **22** ………………………………………………………………………… S42
$^{13}C\{^{1}H\}$ NMR of **22** ………………………………………………………………… S43
^{1}H NMR of **23** ………………………………………………………………………… S44
$^{13}C\{^{1}H\}$ NMR of **23** ………………………………………………………………… S45
$^{31}P\{^{1}H\}$ NMR of **23** ……………………………………………………………….... S46
^{1}H NMR of **11** ………………………………………………………………………… S47
$^{31}P\{^{1}H\}$ NMR of **11** ……………………………………………………………….... S48
HRMS of **11** ……………....………………………………………………………… S49
HPLC profile of **11**…………...…………...……………………………………… S50
MALDI-TOF MS of **13**…………………………………………………………….... S51
MALDI-TOF MS of **24a**……………………………………………………………… S52
MALDI-TOF MS of **24b**……………………………………………………………… S53
MALDI-TOF MS of **26a**……………………………………………………………… S54
MALDI-TOF MS of **26b**……………………………………………………………… S55

Scheme S1. Anti-genomic HDV ribozyme bounding to the modified RNA substrate …….. S56
Figure S1. The representative gel image of the kinetic cleavage of **24b** …………….……... S57

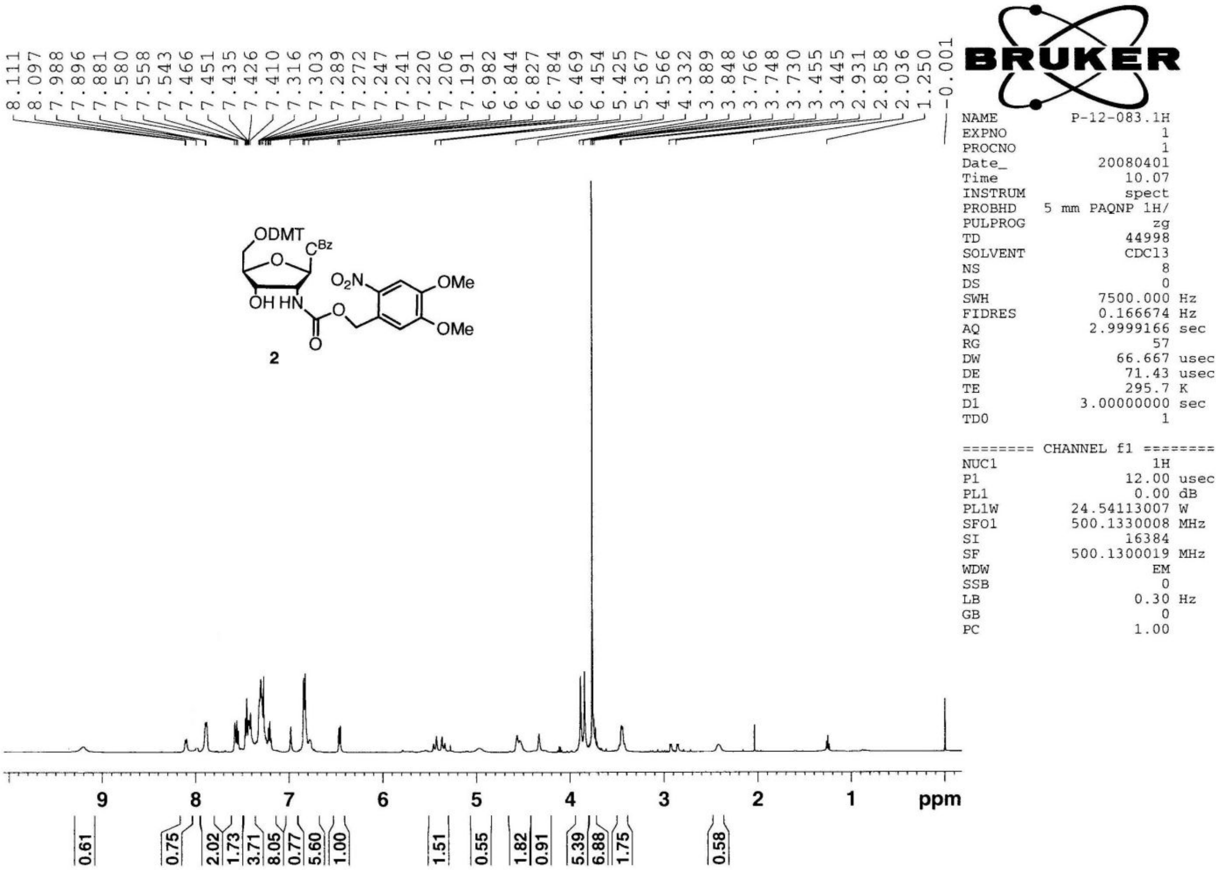
8.111 8.097 7.988 7.896 7.881 7.580 7.558 7.543 7.466 7.451 7.435 7.426 7.410 7.316 7.303 7.289 7.272 7.247 7.241 7.220 7.206 7.191 6.982 6.844 6.827 6.784 6.469 6.454 5.425 5.367 4.566 4.332 3.889 3.848 3.766 3.748 3.730 3.455 3.445 2.931 2.858 2.036 1.250 -0.001
BRUKER
NAME P-12-083.1H
EXPNO 1
PROCNO 1
Date_ 20080401
Time 10.07
INSTRUM spect
PROBHD 5 mm PAQNP 1H/
PULPROG zg
TD 44998
SOLVENT CDCl3
NS 8
DS 0
SWH 7500.000 Hz
FIDRES 0.166674 Hz
AQ 2.9999166 sec
RG 57
DW 66.667 usec
DE 71.43 usec
TE 295.7 K
D1 3.00000000 sec
TD0 1
======== CHANNEL f1 ========
NUC1 1H
P1 12.00 usec
PL1 0.00 dB
PL1W 24.54113007 W
SFO1 500.1330008 MHz
SI 16384
SF 500.1300019 MHz
WDW EM
SSB 0
LB 0.30 Hz
GB 0
PC 1.00
ODMT
Bz
O
O2N
OMe
OH HN
O
OMe
O
2
9 8 7 6 5 4 3 2 1 ppm
0.61 0.75 2.02 1.73 3.71 8.05 0.77 5.60 1.00 1.51 0.55 1.82 0.91 5.39 6.88 1.75 0.58

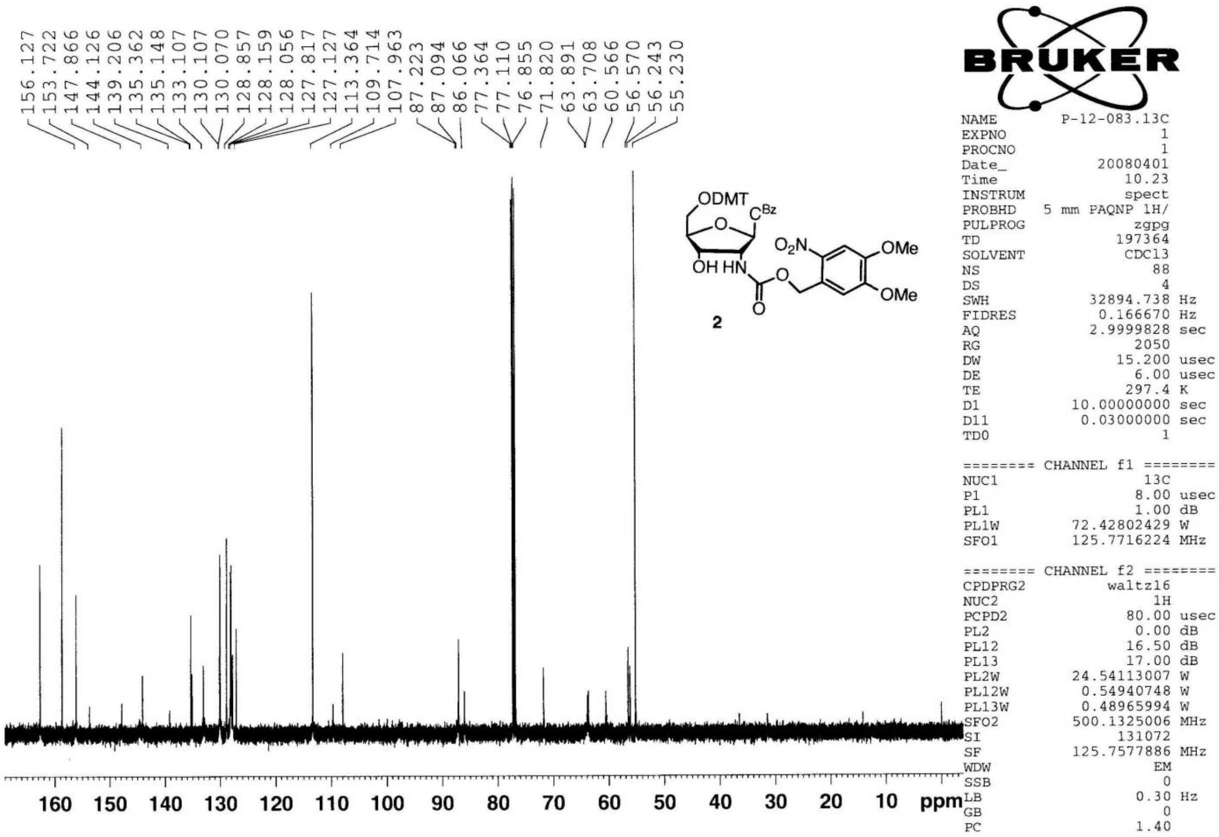
156.127
153.722
147.866
144.126
139.206
135.362
135.148
133.107
130.107
130.070
128.857
128.159
128.056
127.817
127.127
113.364
109.714
107.963
87.223
87.094
86.066
77.364
77.110
76.855
71.820
63.891
63.708
60.566
56.570
56.243
55.230
ODMT
Bz
O_2N
OMe
OMe
OH HN
O
2
160 150 140 130 120 110 100 90 80 70 60 50 40 30 20 10 ppm
BRUKER
NAME P-12-083.13C
EXPNO 1
PROCNO 1
Date_ 20080401
Time 10.23
INSTRUM spect
PROBHD 5 mm PAQNP 1H/
PULPROG zgpg
TD 197364
SOLVENT CDCl3
NS 88
DS 4
SWH 32894.738 Hz
FIDRES 0.166670 Hz
AQ 2.9999828 sec
RG 2050
DW 15.200 usec
DE 6.00 usec
TE 297.4 K
D1 10.00000000 sec
D11 0.03000000 sec
TD0 1
======== CHANNEL f1 ========
NUC1 13C
P1 8.00 usec
PL1 1.00 dB
PL1W 72.42802429 W
SFO1 125.7716224 MHz
======== CHANNEL f2 ========
CPDPRG2 waltz16
NUC2 1H
PCPD2 80.00 usec
PL2 0.00 dB
PL12 16.50 dB
PL13 17.00 dB
PL2W 24.54113007 W
PL12W 0.54940748 W
PL13W 0.48965994 W
SFO2 500.1325006 MHz
SI 131072
SF 125.7577886 MHz
WDW EM
SSB 0
LB 0.30 Hz
GB 0
PC 1.40

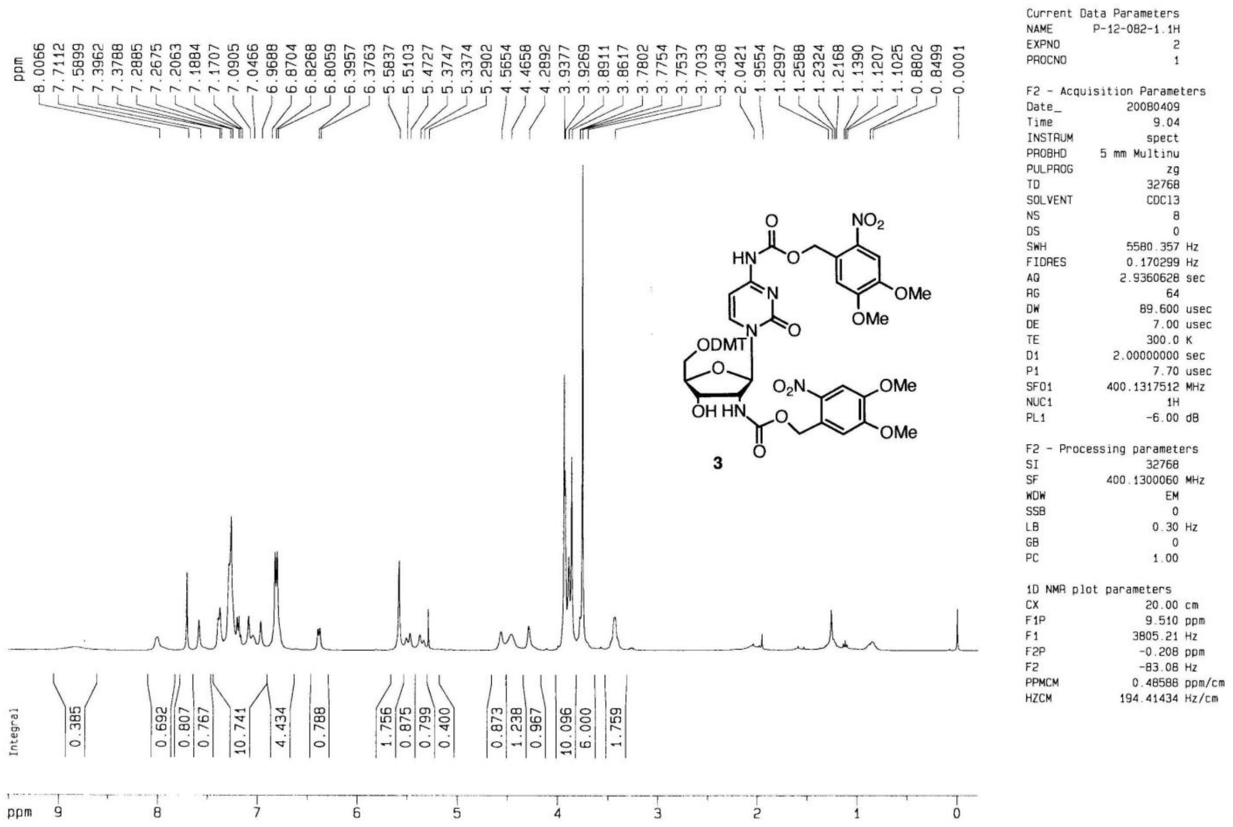
ppm
8.0066 7.7112 7.5899 7.3962 7.3788 7.2885 7.2675 7.2063 7.1884 7.1707 7.0905 7.0466 6.9688 6.8704 6.8268 6.8059 6.3957 6.3763 5.5837 5.5103 5.4727 5.3747 5.3374 5.2902 4.5654 4.4658 4.2892 3.9377 3.9269 3.8911 3.8617 3.7802 3.7754 3.7537 3.7033 3.4308 2.0421 1.9554 1.2997 1.2588 1.2324 1.2168 1.1390 1.1207 1.1025 0.8802 0.8499 0.0001
NO2
HN
OMe
OMe
ODMT
O2N
OMe
OH HN
OMe
3
Integral
0.385 0.692 0.807 0.767 10.741 4.434 0.788 1.756 0.875 0.799 0.400 0.873 1.238 0.967 10.096 6.000 1.759
ppm 9 8 7 6 5 4 3 2 1 0
Current Data Parameters
NAME P-12-082-1.1H
EXPNO 2
PROCNO 1
F2 - Acquisition Parameters
Date_ 20080409
Time 9.04
INSTRUM spect
PROBHD 5 mm Multinu
PULPROG zg
TD 32768
SOLVENT CDCl3
NS 8
DS 0
SWH 5580.357 Hz
FIDRES 0.170299 Hz
AQ 2.9360628 sec
RG 64
DW 89.600 usec
DE 7.00 usec
TE 300.0 K
D1 2.00000000 sec
P1 7.70 usec
SFO1 400.1317512 MHz
NUC1 1H
PL1 -6.00 dB
F2 - Processing parameters
SI 32768
SF 400.1300060 MHz
WDW EM
SSB 0
LB 0.30 Hz
GB 0
PC 1.00
1D NMR plot parameters
CX 20.00 cm
F1P 9.510 ppm
F1 3805.21 Hz
F2P -0.208 ppm
F2 -83.08 Hz
PPMCM 0.48588 ppm/cm
HZCM 194.41434 Hz/cm

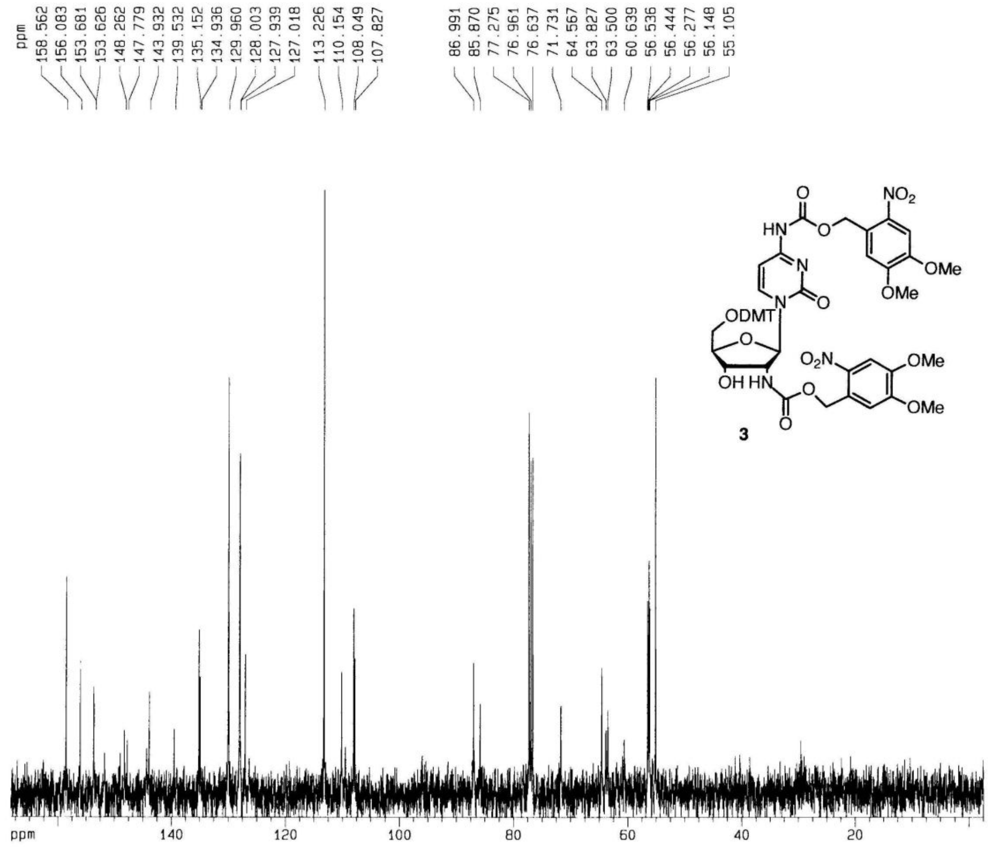

Current Data Parameters
NAME P-12-082-1.13C
EXPNO 2
PROCNO 1

F2 - Acquisition Parameters
Date_ 20080409
Time 9.10
INSTRUM spect
PROBHD 5 mm Multinu
PULPROG zgdc
TD 32768
SOLVENT CDCl3
NS 192
DS 0
SWH 30211.480 Hz
FIDRES 0.921981 Hz
AQ 0.5423604 sec
RG 8192
DW 16.550 usec
DE 23.64 usec
TE 300.0 K
d11 0.03000000 sec
PL12 20.00 dB
CPDPRG2 waltz16
PCPD2 105.00 usec
SFO2 400.1329209 MHz
NUC2 1H
PL2 120.00 dB
D1 2.00000000 sec
P1 6.80 usec
SFO1 100.6223610 MHz
NUC1 13C
PL1 -6.00 dB

F2 - Processing parameters
SI 16384
SF 100.6127809 MHz
WDW EM
SSB 0
LB 1.00 Hz
GB 0
PC 1.00

1D NMR plot parameters
CX 20.00 cm
F1P 168.337 ppm
F1 16936.88 Hz
F2P -2.536 ppm
F2 -255.12 Hz
PPMCM 8.54364 ppm/cm
HZCM 859.59967 Hz/cm

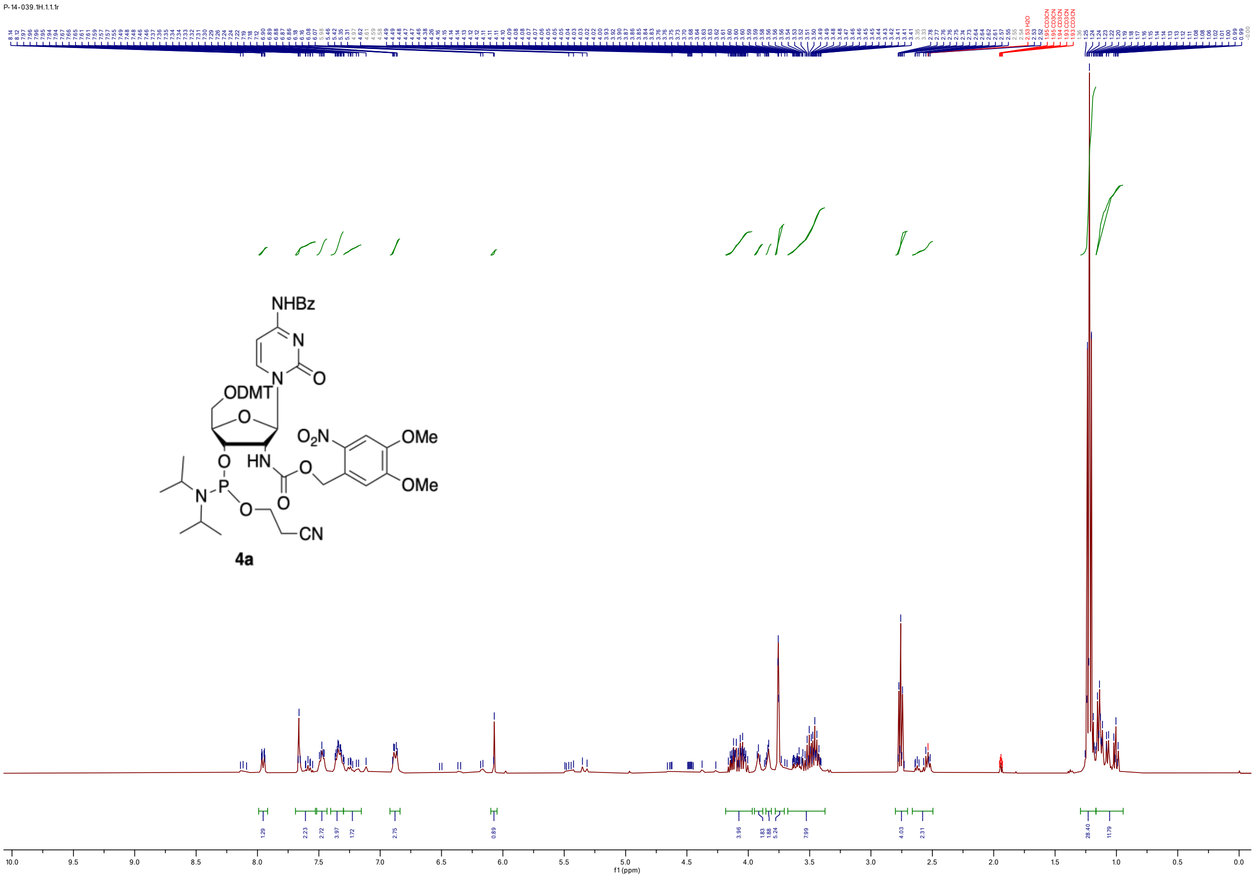
P-14-039.1H.1.1.1r
NHBz
ODMT
O₂N
OMe
OMe
HN
CN
4a
f1 (ppm)

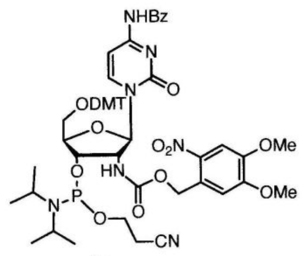

4a

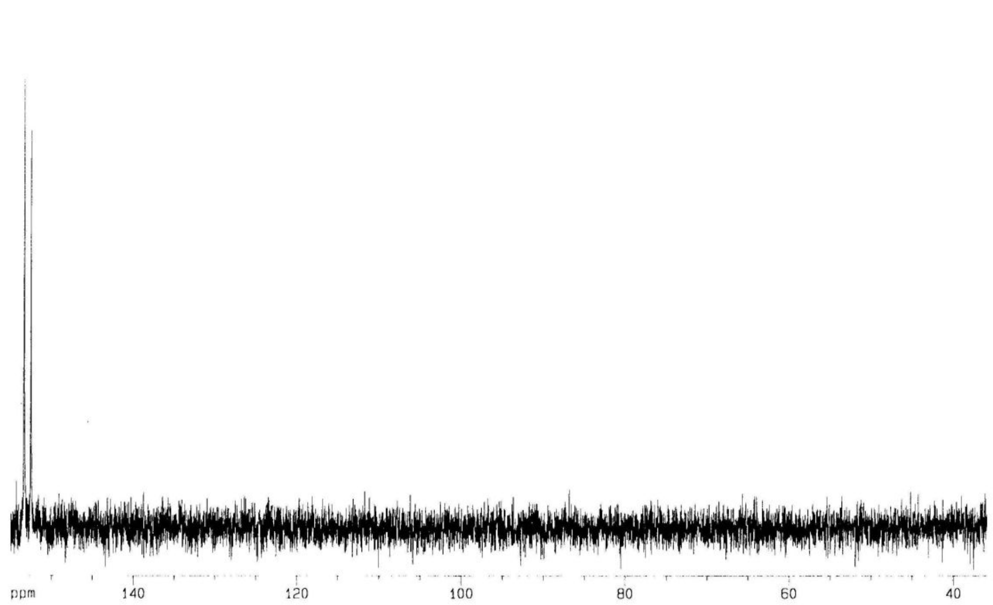

Current Data Parameters
NAME P-12-062308.31
EXPNO 1
PROCNO 1

F2 - Acquisition Parameters
Date_ 20080623
Time 17.09
INSTRUM spect
PROBHD 5 mm Multinu
PULPROG zgdc
TD 32768
SOLVENT CD3CN
NS 4
DS 0
SWH 64935.066 Hz
FIDRES 1.981661 Hz
AQ 0.2523636 sec
RG 5792.6
DW 7.700 usec
DE 11.00 usec
TE 300.0 K
d11 0.03000000 sec
PL12 20.00 dB
CPDPRG2 waltz16
PCPD2 100.00 usec
SFO2 400.1329209 MHz
NUC2 1H
PL2 120.00 dB
D1 1.00000000 sec
P1 6.00 usec
SFO1 161.9834918 MHz
NUC1 31P
PL1 -6.00 dB

F2 - Processing parameters
SI 32768
SF 161.9750852 MHz
WDW EM
SSB 0
LB 1.00 Hz
GB 0
PC 1.40

1D NMR plot parameters
CX 20.00 cm
F1P 154.976 ppm
F1 25102.27 Hz
F2P 35.893 ppm
F2 5813.75 Hz
PPMCM 5.95416 ppm/cm
HZCM 964.42609 Hz/cm

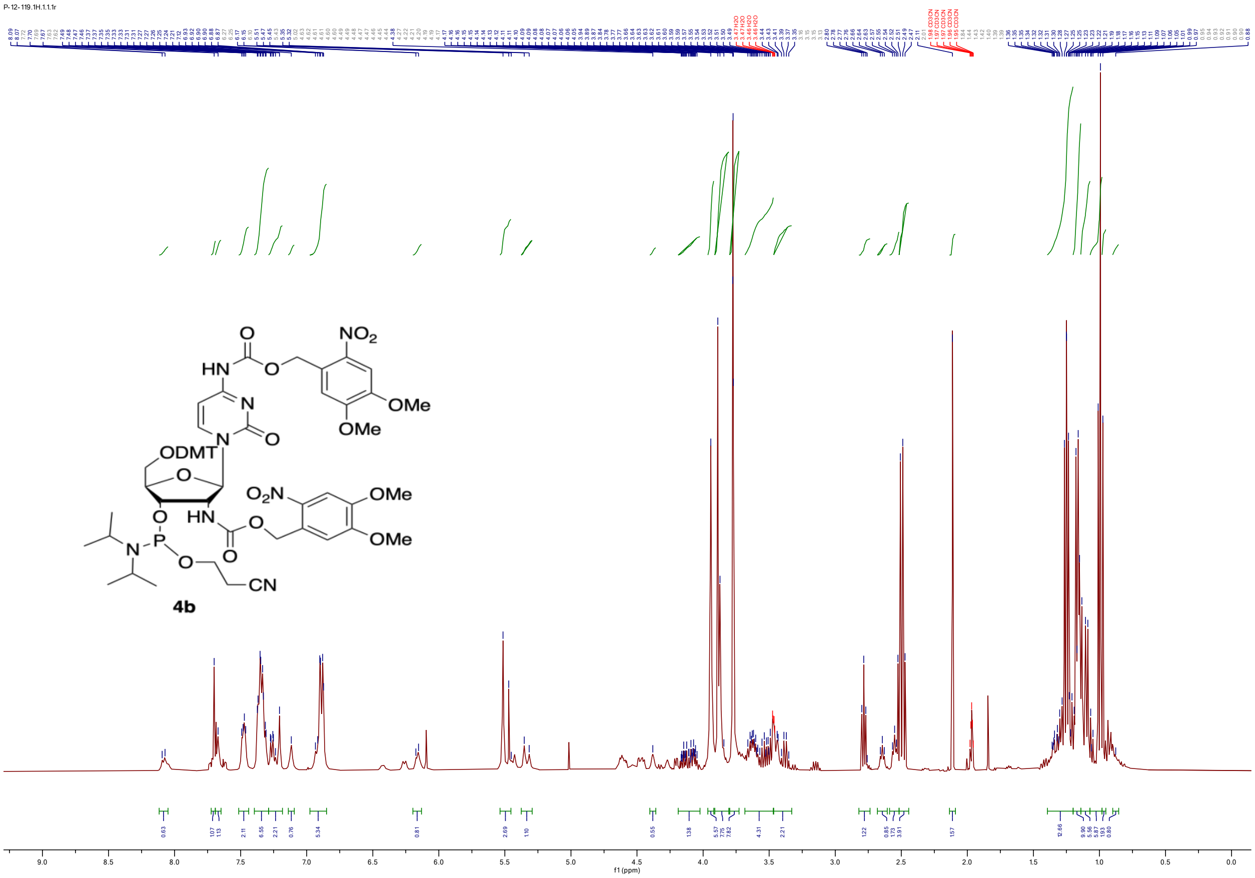
P-12-119.1H.1.1.1r
NO2
HN
O
N
OMe
ODMT
O2N
HN
OMe
N
P
CN
4b
f1 (ppm)

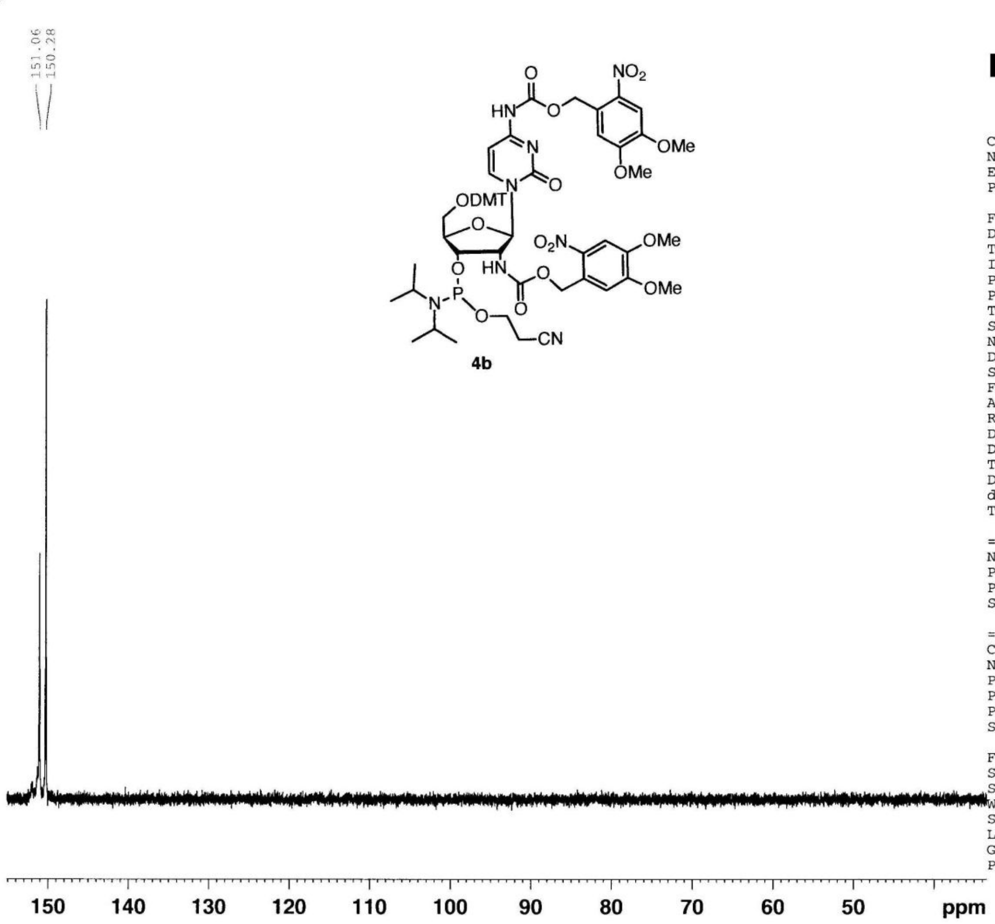

BRUKER

```
Current Data Parameters
NAME          P-12-119.31P
EXPNO                    2
PROCNO                   1

F2 - Acquisition Parameters
Date_             20081009
Time                 13.21
INSTRUM              spect
PROBHD   5 mm BBO BB-1H
PULPROG               zgdc
TD                  162596
SOLVENT               CD3CN
NS                       8
DS                       0
SWH              40000.000 Hz
FIDRES            0.246009 Hz
AQ               2.0325000 sec
RG                  9195.2
DW                  12.500 usec
DE                    6.00 usec
TE                   297.4 K
D1              3.00000000 sec
d11             0.03000000 sec
TD0                      1

======== CHANNEL f1 ========
NUC1                   31P
P1                    8.80 usec
PL1                  -4.00 dB
SFO1           161.9869984 MHz

======== CHANNEL f2 ========
CPDPRG2            waltz16
NUC2                    1H
PCPD2                80.00 usec
PL2                  -6.00 dB
PL12                 17.00 dB
SFO2           400.1328010 MHz

F2 - Processing parameters
SI                   32768
SF             161.9754607 MHz
WDW                     EM
SSB                      0
LB                    1.00 Hz
GB                       0
PC                    1.40
```

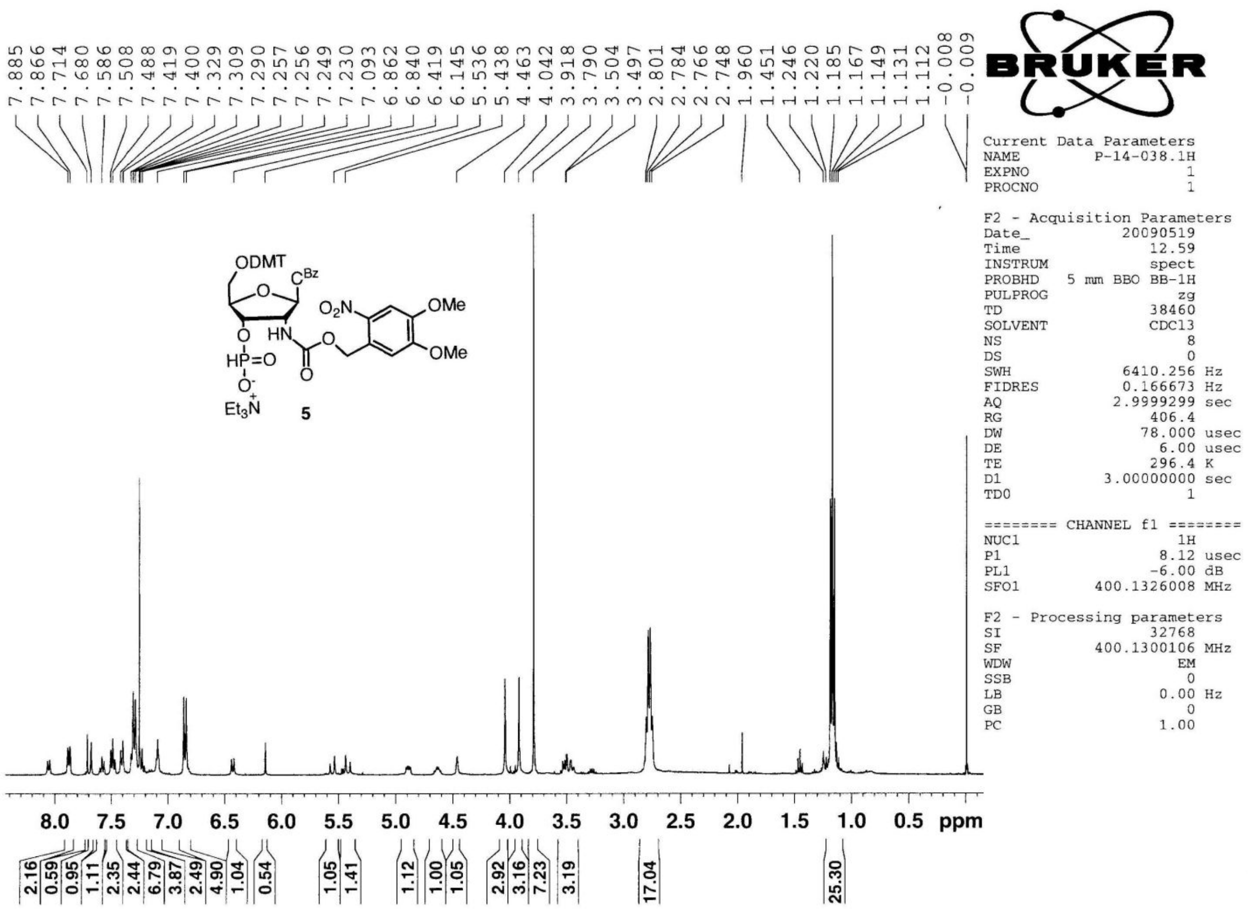

Current Data Parameters
NAME P-14-038.1H
EXPNO 1
PROCNO 1
F2 - Acquisition Parameters
Date_ 20090519
Time 12.59
INSTRUM spect
PROBHD 5 mm BBO BB-1H
PULPROG zg
TD 38460
SOLVENT CDCl3
NS 8
DS 0
SWH 6410.256 Hz
FIDRES 0.166673 Hz
AQ 2.9999299 sec
RG 406.4
DW 78.000 usec
DE 6.00 usec
TE 296.4 K
D1 3.00000000 sec
TD0 1
======== CHANNEL f1 ========
NUC1 1H
P1 8.12 usec
PL1 -6.00 dB
SFO1 400.1326008 MHz
F2 - Processing parameters
SI 32768
SF 400.1300106 MHz
WDW EM
SSB 0
LB 0.00 Hz
GB 0
PC 1.00
ppm

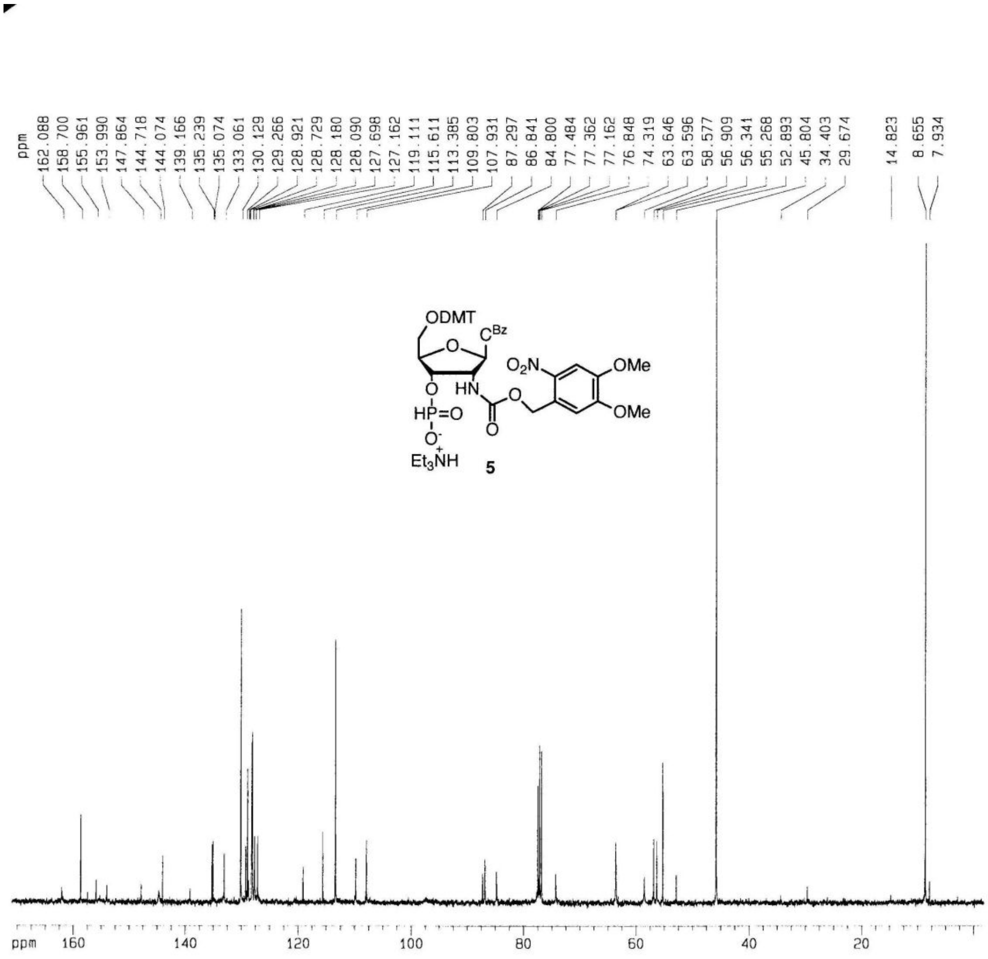

Current Data Parameters
NAME P-12-090.13C
EXPNO 1
PROCNO 1

F2 - Acquisition Parameters

Date_	20080428	
Time	7.38	
INSTRUM	spect	
PROBHD	5 mm Multinu	
PULPROG	zgdc	
TD	32768	
SOLVENT	CDCl3	
NS	1490	
DS	0	
SWH	30211.480	Hz
FIDRES	0.921981	Hz
AQ	0.5423604	sec
RG	11585.2	
DW	16.550	usec
DE	23.64	usec
TE	300.0	K
d11	0.03000000	sec
PL12	20.00	dB
CPDPRG2	waltz16	
PCPD2	105.00	usec
SFO2	400.1329209	MHz
NUC2	1H	
PL2	120.00	dB
D1	2.00000000	sec
P1	6.80	usec
SFO1	100.6223610	MHz
NUC1	13C	
PL1	-6.00	dB

F2 - Processing parameters

SI	16384	
SF	100.6127667	MHz
WDW	EM	
SSB	0	
LB	1.00	Hz
GB	0	
PC	1.00	

1D NMR plot parameters

CX	20.00	cm
F1P	170.973	ppm
F1	17202.12	Hz
F2P	-1.770	ppm
F2	-178.11	Hz
PPMCM	8.63719	ppm/cm
HZCM	869.01147	Hz/cm

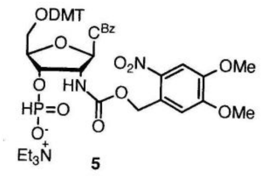

5

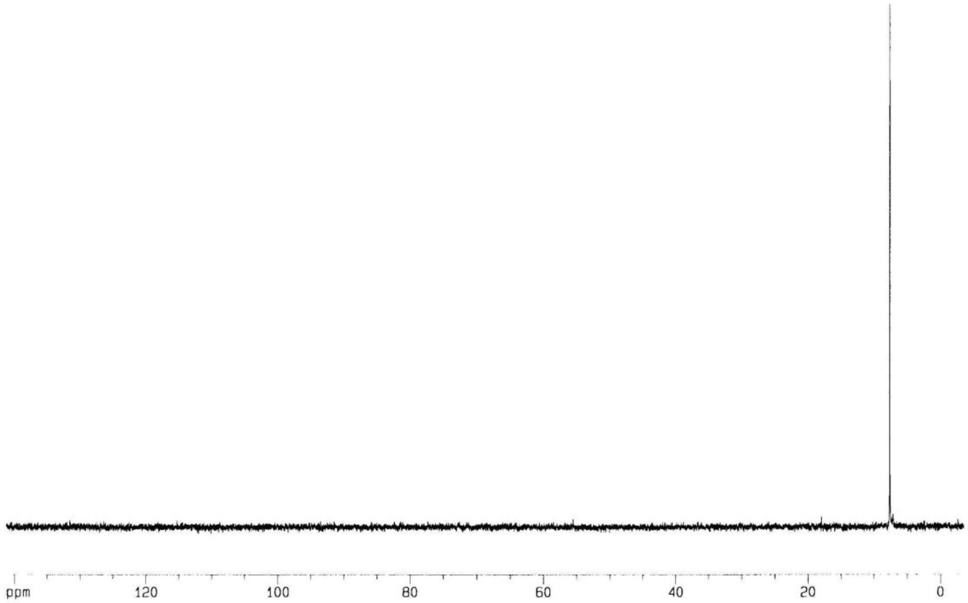

Current Data Parameters
NAME P-12-090.31P
EXPNO 1
PROCNO 1

F2 - Acquisition Parameters
Date_ 20080428
Time 7.29
INSTRUM spect
PROBHD 5 mm Multinu
PULPROG zgdc
TD 32768
SOLVENT ~~CD3CN~~ $CDCl_3$
NS 8
DS 0
SWH 64935.066 Hz
FIDRES 1.981661 Hz
AQ 0.2523636 sec
RG 9195.2
DW 7.700 usec
DE 11.00 usec
TE 300.0 K
d11 0.03000000 sec
PL12 20.00 dB
CPDPRG2 waltz16
PCPD2 100.00 usec
SFO2 400.1329209 MHz
NUC2 1H
PL2 120.00 dB
D1 1.00000000 sec
P1 6.00 usec
SFO1 161.9834918 MHz
NUC1 31P
PL1 -6.00 dB

F2 - Processing parameters
SI 32768
SF 161.9750852 MHz
WDW EM
SSB 0
LB 1.00 Hz
GB 0
PC 1.40

1D NMR plot parameters
CX 20.00 cm
F1P 141.217 ppm
F1 22873.62 Hz
F2P -3.442 ppm
F2 -557.57 Hz
PPMCM 7.23296 ppm/cm
HZCM 1171.55896 Hz/cm

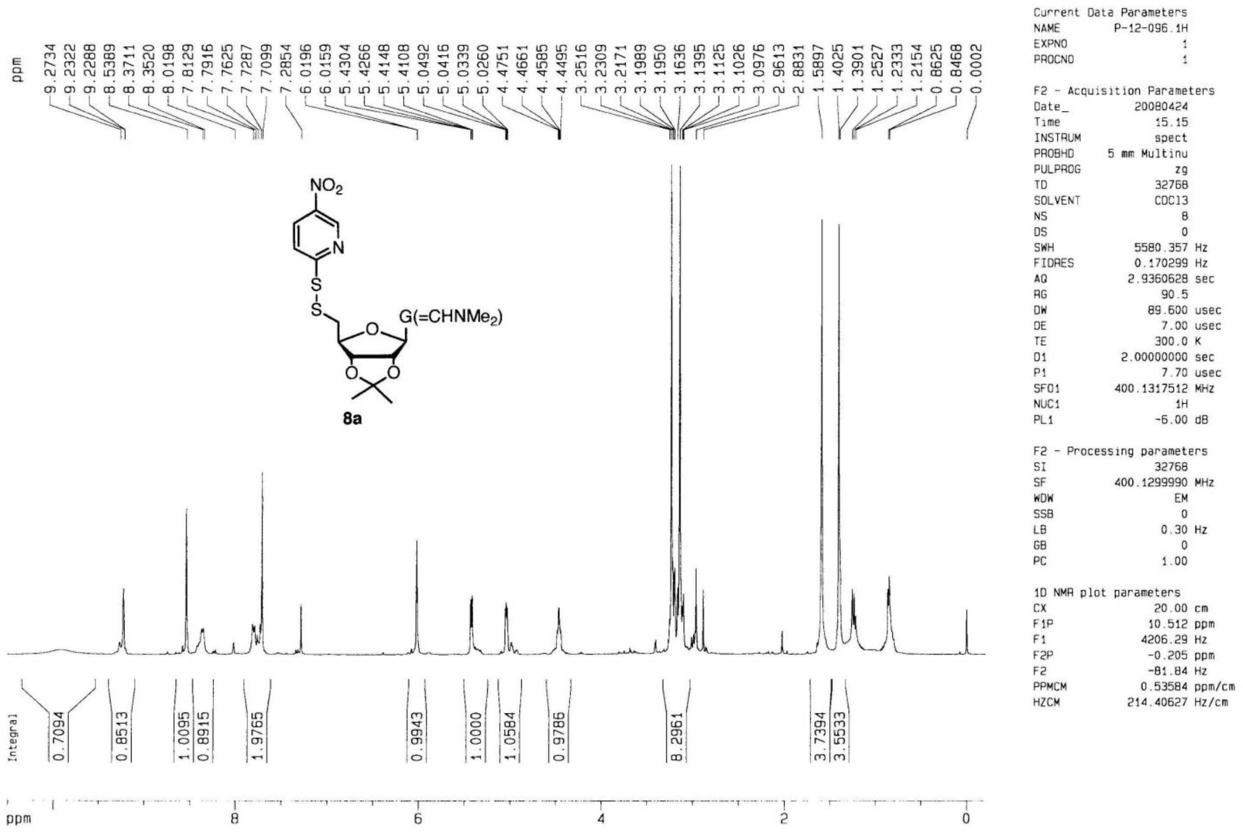
NO2
N
S
S
O
G(=CHNMe2)
8a
ppm
9.2734
9.2322
9.2288
8.5389
8.3711
8.3520
8.0198
7.8129
7.7916
7.7625
7.7287
7.7099
7.2854
6.0196
6.0159
5.4304
5.4266
5.4148
5.4108
5.0492
5.0416
5.0339
5.0260
4.4751
4.4661
4.4585
4.4495
3.2516
3.2309
3.2171
3.1989
3.1950
3.1636
3.1395
3.1125
3.1026
3.0976
2.9613
2.8831
1.5897
1.4025
1.3901
1.2527
1.2333
1.2154
0.8625
0.8468
0.0002
Integral
0.7094
0.8513
1.0095
0.8915
1.9765
0.9943
1.0000
1.0584
0.9786
8.2961
3.7394
3.5533
ppm
8
6
4
2
0
Current Data Parameters
NAME P-12-096.1H
EXPNO 1
PROCNO 1
F2 - Acquisition Parameters
Date_ 20080424
Time 15.15
INSTRUM spect
PROBHD 5 mm Multinu
PULPROG zg
TD 32768
SOLVENT CDCl3
NS 8
DS 0
SWH 5580.357 Hz
FIDRES 0.170299 Hz
AQ 2.9360628 sec
RG 90.5
DW 89.600 usec
DE 7.00 usec
TE 300.0 K
D1 2.00000000 sec
P1 7.70 usec
SFO1 400.1317512 MHz
NUC1 1H
PL1 -6.00 dB
F2 - Processing parameters
SI 32768
SF 400.1299990 MHz
WDW EM
SSB 0
LB 0.30 Hz
GB 0
PC 1.00
1D NMR plot parameters
CX 20.00 cm
F1P 10.512 ppm
F1 4206.29 Hz
F2P -0.205 ppm
F2 -81.84 Hz
PPMCM 0.53584 ppm/cm
HZCM 214.40627 Hz/cm

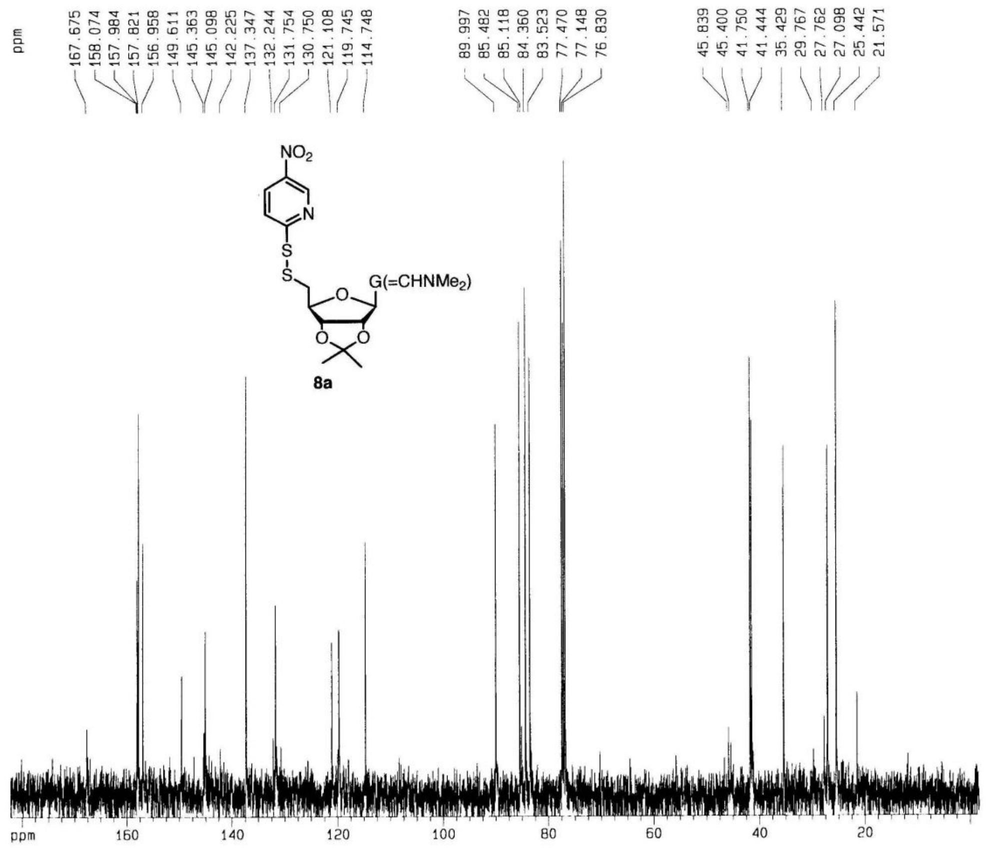

Current Data Parameters

NAME	P-12-096.13C
EXPNO	1
PROCNO	1

F2 - Acquisition Parameters

Date_	20080424
Time	15.21
INSTRUM	spect
PROBHD	5 mm Multinu
PULPROG	zgdc
TD	32768
SOLVENT	CDCl3
NS	531
DS	0
SWH	30211.480 Hz
FIDRES	0.921981 Hz
AQ	0.5423604 sec
RG	5160.6
DW	16.550 usec
DE	23.64 usec
TE	300.0 K
d11	0.03000000 sec
PL12	20.00 dB
CPDPRG2	waltz16
PCPD2	105.00 usec
SFO2	400.1329209 MHz
NUC2	1H
PL2	120.00 dB
D1	2.00000000 sec
P1	6.80 usec
SFO1	100.6223610 MHz
NUC1	13C
PL1	-6.00 dB

F2 - Processing parameters

SI	16384
SF	100.6127611 MHz
WDW	EM
SSB	0
LB	1.00 Hz
GB	0
PC	1.00

1D NMR plot parameters

CX	20.00 cm
F1P	182.254 ppm
F1	18337.04 Hz
F2P	-1.715 ppm
F2	-172.59 Hz
PPMCM	9.19845 ppm/cm
HZCM	925.48151 Hz/cm

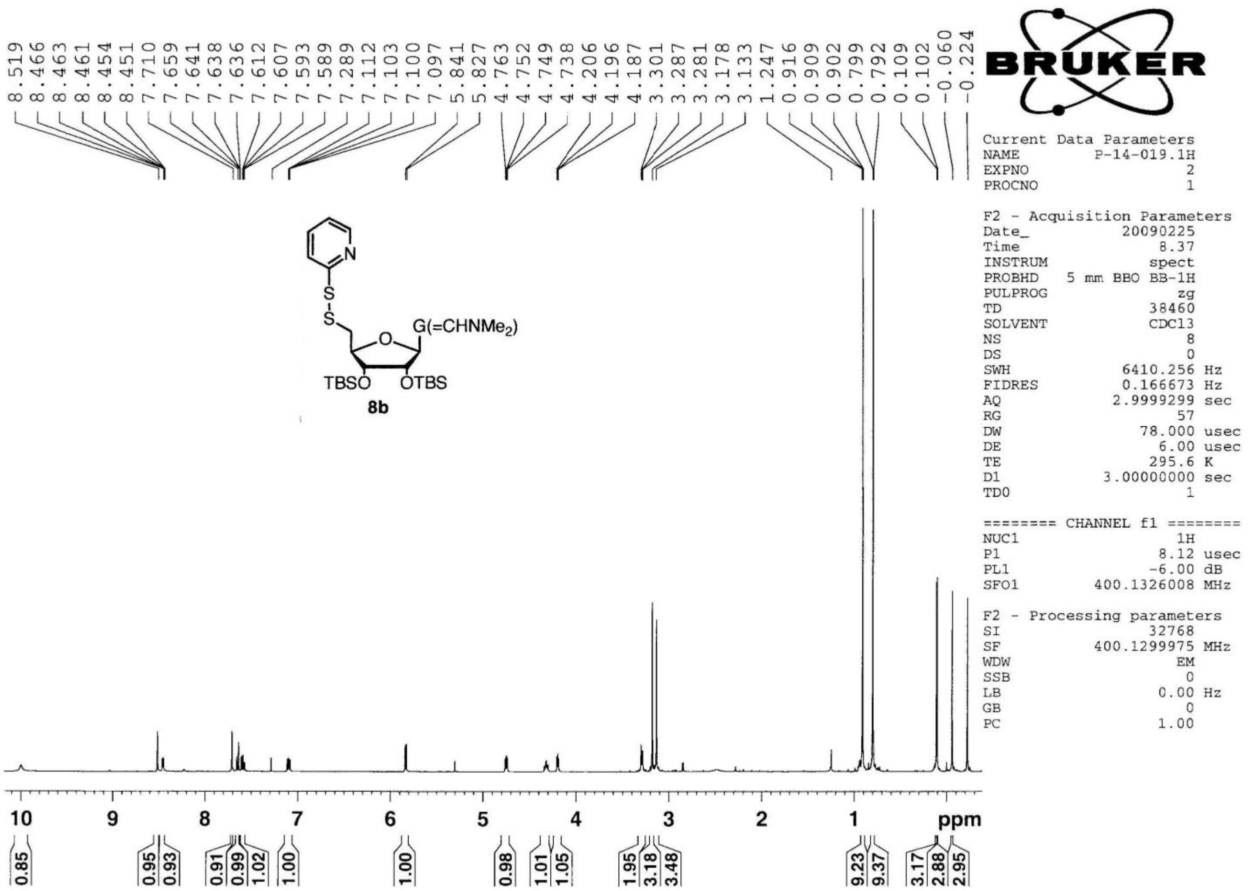
Current Data Parameters
NAME P-14-019.1H
EXPNO 2
PROCNO 1
F2 - Acquisition Parameters
Date_ 20090225
Time 8.37
INSTRUM spect
PROBHD 5 mm BBO BB-1H
PULPROG zg
TD 38460
SOLVENT CDCl3
NS 8
DS 0
SWH 6410.256 Hz
FIDRES 0.166673 Hz
AQ 2.9999299 sec
RG 57
DW 78.000 usec
DE 6.00 usec
TE 295.6 K
D1 3.00000000 sec
TD0 1
======== CHANNEL f1 ========
NUC1 1H
P1 8.12 usec
PL1 -6.00 dB
SFO1 400.1326008 MHz
F2 - Processing parameters
SI 32768
SF 400.1299975 MHz
WDW EM
SSB 0
LB 0.00 Hz
GB 0
PC 1.00
G(=CHNMe2)
TBSO
OTBS
8b
ppm

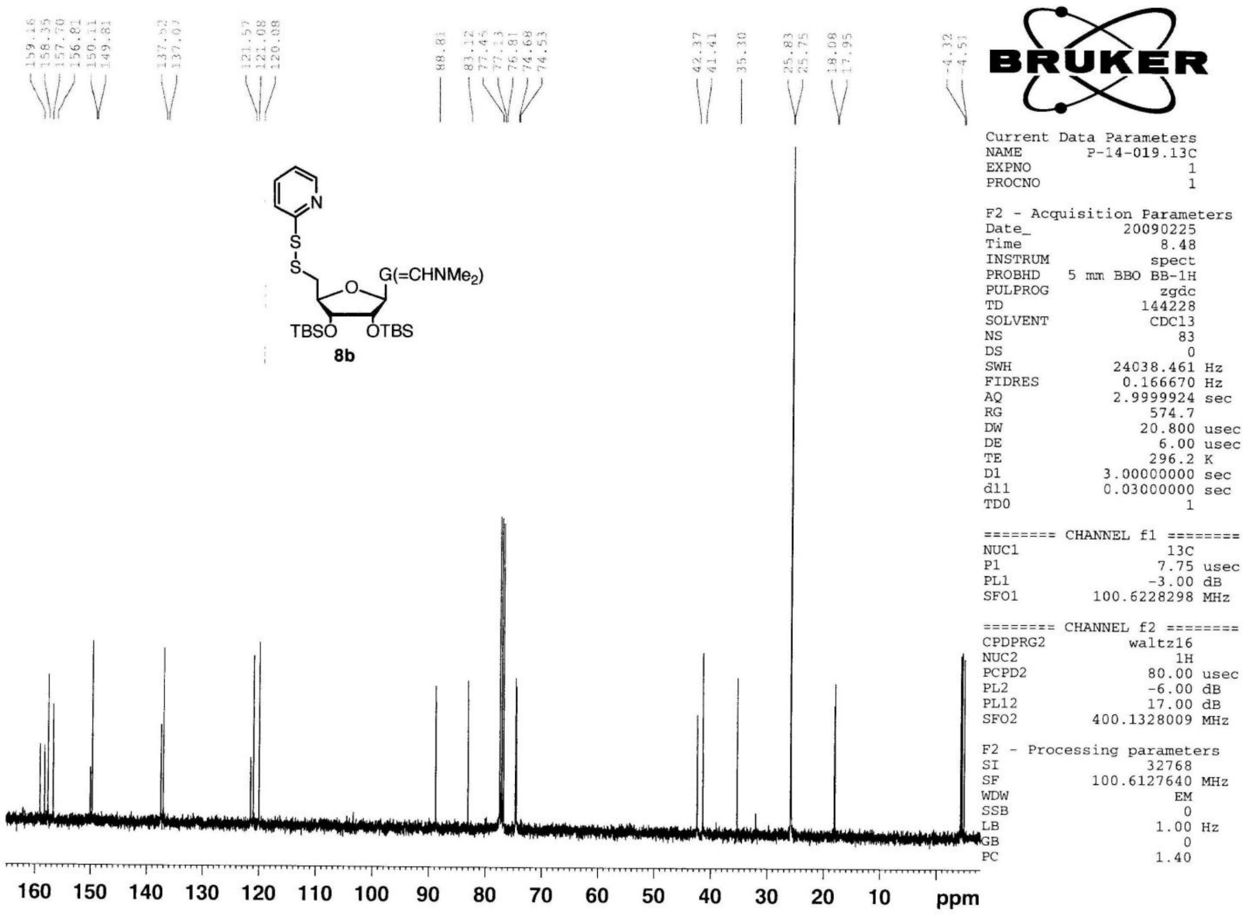
159.16 158.35 157.70 156.81 150.11 149.81
137.52 137.07
121.57 121.08 120.08
88.81
83.12 77.45 77.13 76.81 74.68 74.53
42.37 41.41
35.30
25.83 25.75
18.08 17.95
-4.32 -4.51
G(=CHNMe2)
TBSO OTBS
8b
BRUKER
Current Data Parameters
NAME P-14-019.13C
EXPNO 1
PROCNO 1
F2 - Acquisition Parameters
Date_ 20090225
Time 8.48
INSTRUM spect
PROBHD 5 mm BBO BB-1H
PULPROG zgdc
TD 144228
SOLVENT CDCl3
NS 83
DS 0
SWH 24038.461 Hz
FIDRES 0.166670 Hz
AQ 2.9999924 sec
RG 574.7
DW 20.800 usec
DE 6.00 usec
TE 296.2 K
D1 3.00000000 sec
d11 0.03000000 sec
TD0 1
======== CHANNEL f1 ========
NUC1 13C
P1 7.75 usec
PL1 -3.00 dB
SFO1 100.6228298 MHz
======== CHANNEL f2 ========
CPDPRG2 waltz16
NUC2 1H
PCPD2 80.00 usec
PL2 -6.00 dB
PL12 17.00 dB
SFO2 400.1328009 MHz
F2 - Processing parameters
SI 32768
SF 100.6127640 MHz
WDW EM
SSB 0
LB 1.00 Hz
GB 0
PC 1.40
160 150 140 130 120 110 100 90 80 70 60 50 40 30 20 10 ppm

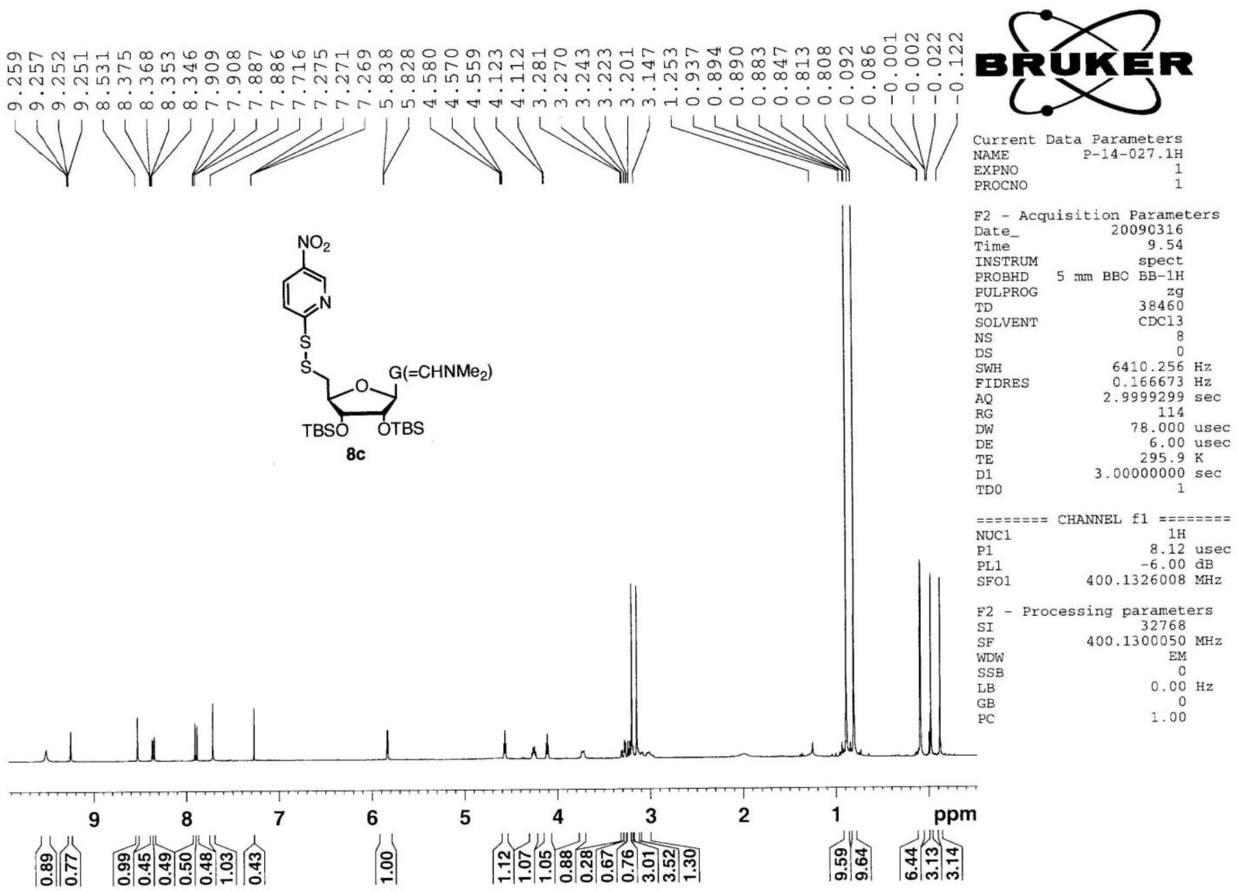
NO2
N
S
S
G(=CHNMe2)
O
TBSO
OTBS
8c
BRUKER
Current Data Parameters
NAME P-14-027.1H
EXPNO 1
PROCNO 1
F2 - Acquisition Parameters
Date_ 20090316
Time 9.54
INSTRUM spect
PROBHD 5 mm BBO BB-1H
PULPROG zg
TD 38460
SOLVENT CDCl3
NS 8
DS 0
SWH 6410.256 Hz
FIDRES 0.166673 Hz
AQ 2.9999299 sec
RG 114
DW 78.000 usec
DE 6.00 usec
TE 295.9 K
D1 3.00000000 sec
TD0 1
======== CHANNEL f1 ========
NUC1 1H
P1 8.12 usec
PL1 -6.00 dB
SFO1 400.1326008 MHz
F2 - Processing parameters
SI 32768
SF 400.1300050 MHz
WDW EM
SSB 0
LB 0.00 Hz
GB 0
PC 1.00
ppm

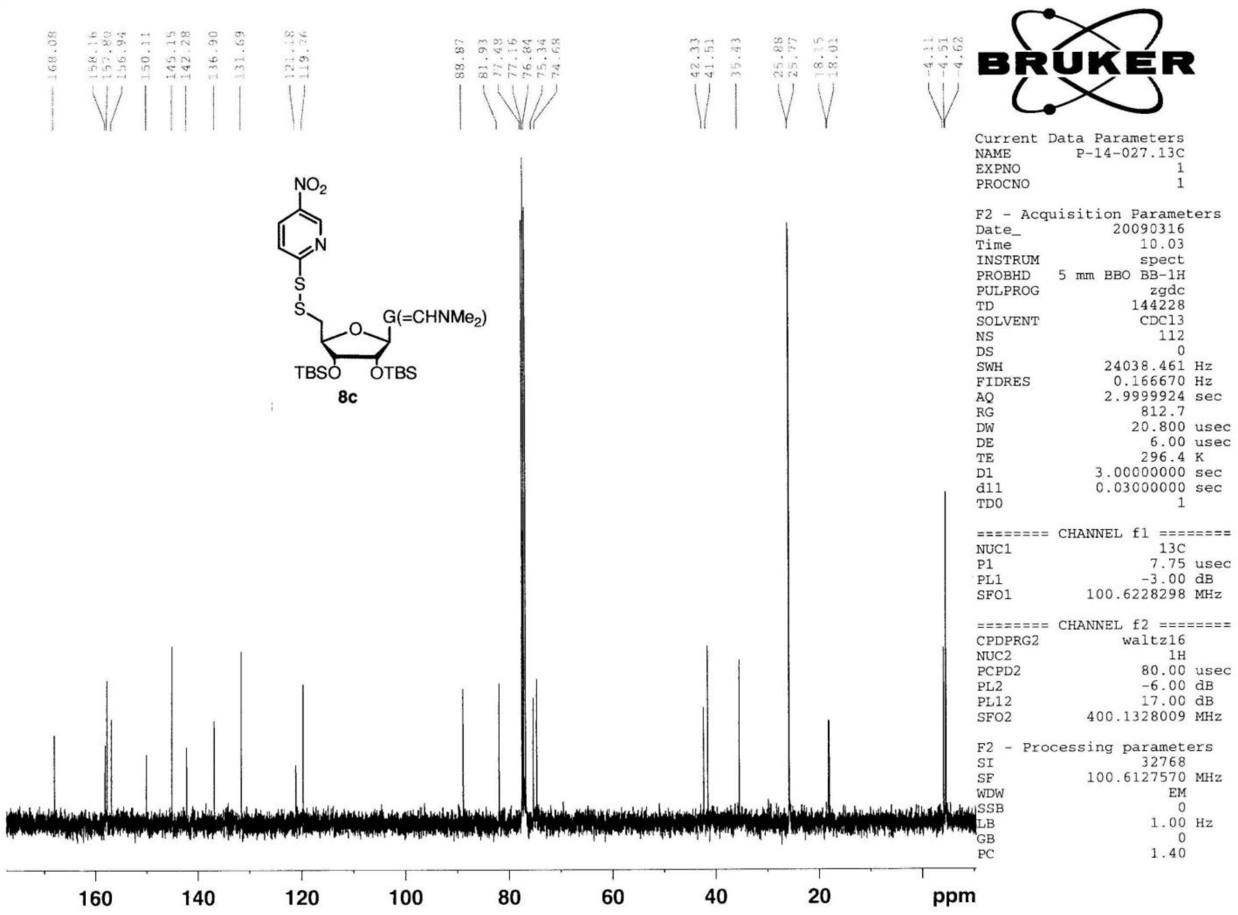

168.08
158.16
157.80
156.94
150.11
145.15
142.28
136.90
131.69
121.18
119.74
88.87
81.93
77.48
77.16
76.84
75.34
74.68
42.33
41.51
35.43
25.88
25.77
18.15
18.01
-4.11
-4.51
-4.62
NO_2
N
S
S
O
G(=CHNMe$_2$)
TBSO
OTBS
8c
BRUKER
Current Data Parameters
NAME P-14-027.13C
EXPNO 1
PROCNO 1
F2 - Acquisition Parameters
Date_ 20090316
Time 10.03
INSTRUM spect
PROBHD 5 mm BBO BB-1H
PULPROG zgdc
TD 144228
SOLVENT CDCl3
NS 112
DS 0
SWH 24038.461 Hz
FIDRES 0.166670 Hz
AQ 2.9999924 sec
RG 812.7
DW 20.800 usec
DE 6.00 usec
TE 296.4 K
D1 3.00000000 sec
d11 0.03000000 sec
TD0 1
======== CHANNEL f1 ========
NUC1 13C
P1 7.75 usec
PL1 -3.00 dB
SFO1 100.6228298 MHz
======== CHANNEL f2 ========
CPDPRG2 waltz16
NUC2 1H
PCPD2 80.00 usec
PL2 -6.00 dB
PL12 17.00 dB
SFO2 400.1328009 MHz
F2 - Processing parameters
SI 32768
SF 100.6127570 MHz
WDW EM
SSB 0
LB 1.00 Hz
GB 0
PC 1.40
160
140
120
100
80
60
40
20
ppm

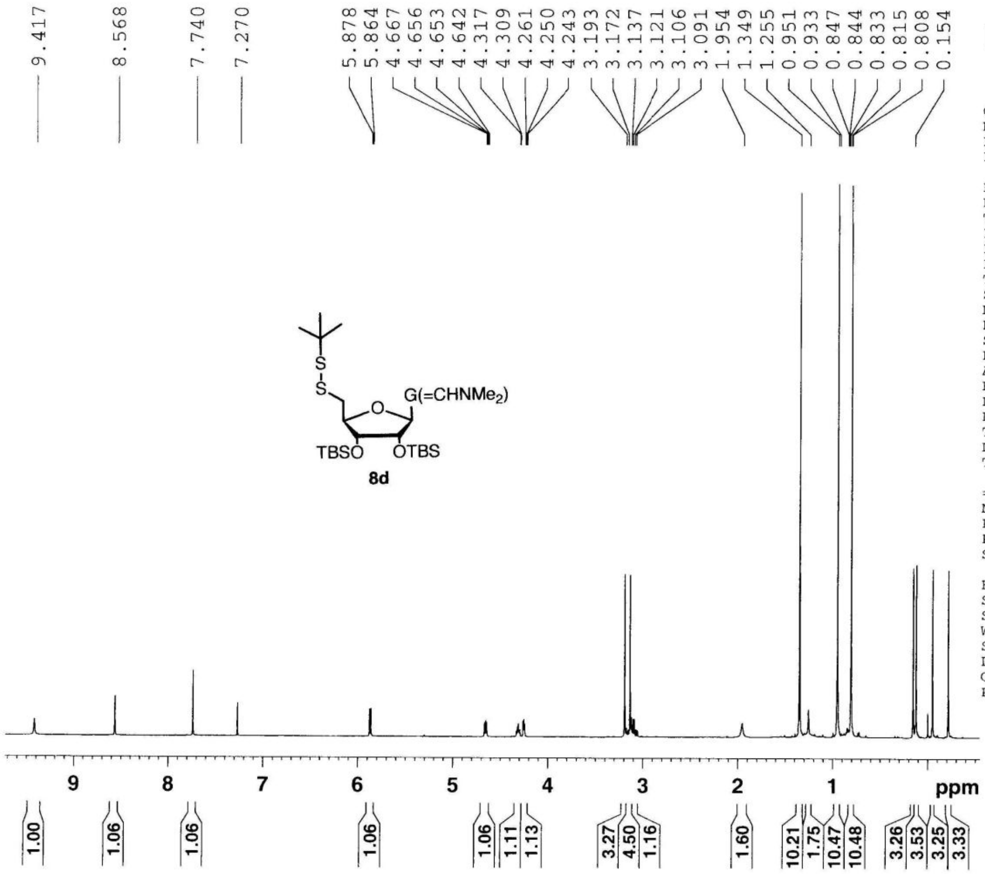

Current Data Parameters
NAME P-14-022.1H
EXPNO 1
PROCNO 1

F2 - Acquisition Parameters
Date_ 20090226
Time 8.42
INSTRUM spect
PROBHD 5 mm BBO BB-1H
PULPROG zg
TD 38460
SOLVENT CDCl3
NS 8
DS 0
SWH 6410.256 Hz
FIDRES 0.166673 Hz
AQ 2.9999299 sec
RG 128
DW 78.000 usec
DE 6.00 usec
TE 296.1 K
D1 3.00000000 sec
TD0 1

======== CHANNEL f1 ========
NUC1 1H
P1 8.12 usec
PL1 -6.00 dB
SFO1 400.1326008 MHz

F2 - Processing parameters
SI 32768
SF 400.1300052 MHz
WDW EM
SSB 0
LB 0.00 Hz
GB 0
PC 1.00

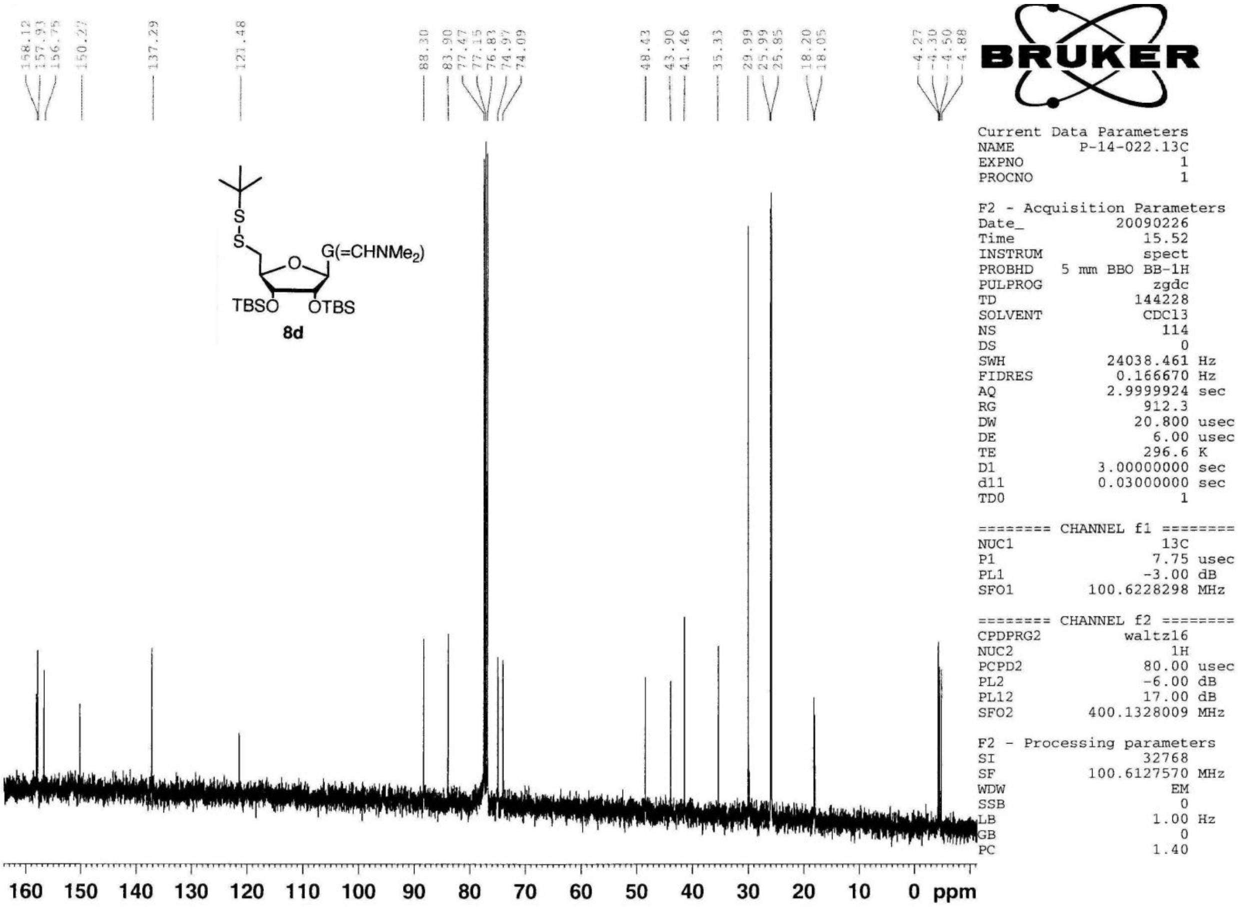

158.12
157.93
156.75
150.27
137.29
121.48
88.30
83.90
77.47
77.15
76.83
74.97
74.09
48.43
43.90
41.46
35.33
29.99
25.99
25.85
18.20
18.05
-4.27
-4.30
-4.50
-4.88
S
S
O
G(=CHNMe2)
TBSO
OTBS
8d
BRUKER
Current Data Parameters
NAME P-14-022.13C
EXPNO 1
PROCNO 1
F2 - Acquisition Parameters
Date_ 20090226
Time 15.52
INSTRUM spect
PROBHD 5 mm BBO BB-1H
PULPROG zgdc
TD 144228
SOLVENT CDCl3
NS 114
DS 0
SWH 24038.461 Hz
FIDRES 0.166670 Hz
AQ 2.9999924 sec
RG 912.3
DW 20.800 usec
DE 6.00 usec
TE 296.6 K
D1 3.00000000 sec
d11 0.03000000 sec
TD0 1
======== CHANNEL f1 ========
NUC1 13C
P1 7.75 usec
PL1 -3.00 dB
SFO1 100.6228298 MHz
======== CHANNEL f2 ========
CPDPRG2 waltz16
NUC2 1H
PCPD2 80.00 usec
PL2 -6.00 dB
PL12 17.00 dB
SFO2 400.1328009 MHz
F2 - Processing parameters
SI 32768
SF 100.6127570 MHz
WDW EM
SSB 0
LB 1.00 Hz
GB 0
PC 1.40
160 150 140 130 120 110 100 90 80 70 60 50 40 30 20 10 0 ppm

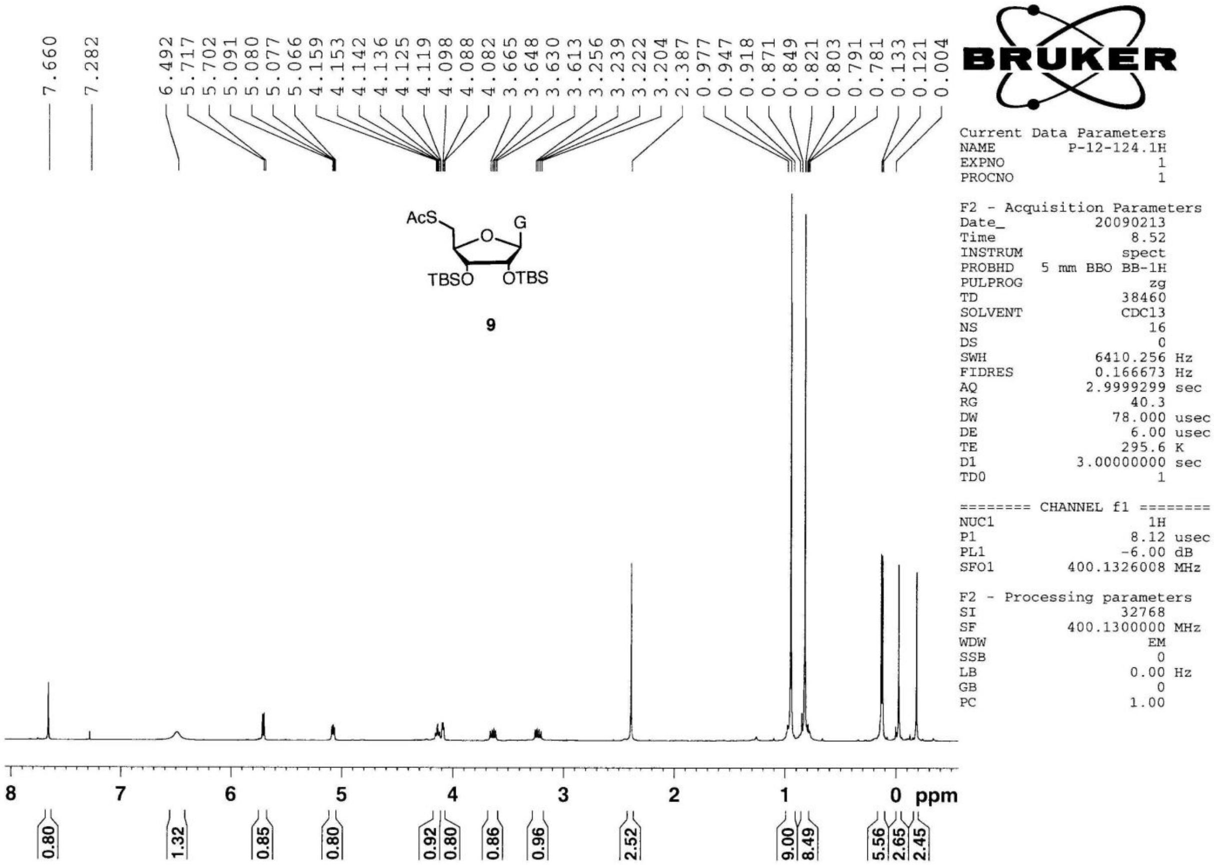
7.660
7.282
6.492
5.717
5.702
5.091
5.080
5.077
5.066
4.159
4.153
4.142
4.136
4.125
4.119
4.098
4.088
4.082
3.665
3.648
3.630
3.613
3.256
3.239
3.222
3.204
2.387
0.977
0.947
0.918
0.871
0.849
0.821
0.803
0.791
0.781
0.133
0.121
0.004
BRUKER
AcS
O
G
TBSO
OTBS
9
Current Data Parameters
NAME P-12-124.1H
EXPNO 1
PROCNO 1
F2 - Acquisition Parameters
Date_ 20090213
Time 8.52
INSTRUM spect
PROBHD 5 mm BBO BB-1H
PULPROG zg
TD 38460
SOLVENT CDCl3
NS 16
DS 0
SWH 6410.256 Hz
FIDRES 0.166673 Hz
AQ 2.9999299 sec
RG 40.3
DW 78.000 usec
DE 6.00 usec
TE 295.6 K
D1 3.00000000 sec
TD0 1
======== CHANNEL f1 ========
NUC1 1H
P1 8.12 usec
PL1 -6.00 dB
SFO1 400.1326008 MHz
F2 - Processing parameters
SI 32768
SF 400.1300000 MHz
WDW EM
SSB 0
LB 0.00 Hz
GB 0
PC 1.00
8
7
6
5
4
3
2
1
0 ppm
0.80
1.32
0.85
0.80
0.92
0.80
0.86
0.96
2.52
9.00
8.49
5.56
2.65
2.45

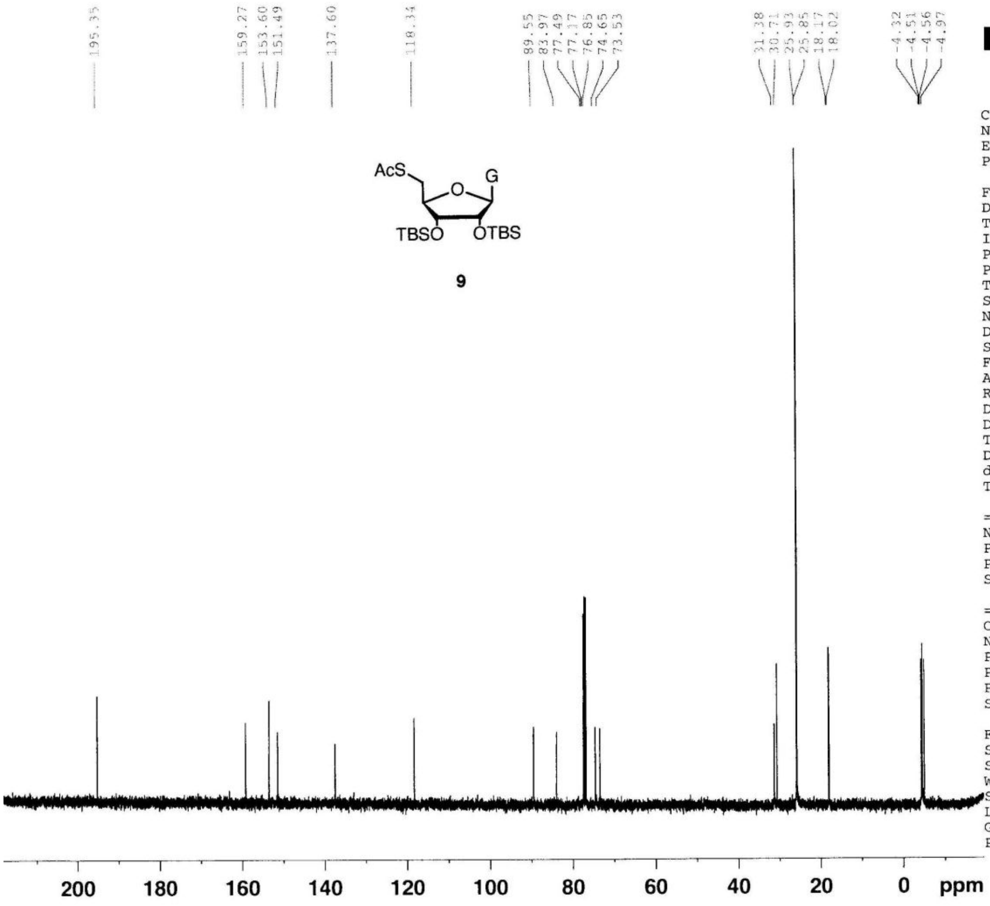


```
Current Data Parameters
NAME          P-12-124.13C
EXPNO                    1
PROCNO                   1

F2 - Acquisition Parameters
Date_             20090213
Time                  8.58
INSTRUM              spect
PROBHD    5 mm BBO BB-1H
PULPROG               zgdc
TD                  144228
SOLVENT              CDCl3
NS                      43
DS                       0
SWH          24038.461 Hz
FIDRES        0.166670 Hz
AQ           2.9999924 sec
RG                  1290.2
DW              20.800 usec
DE               6.00 usec
TE               296.0 K
D1          3.00000000 sec
d11         0.03000000 sec
TD0                      1

======== CHANNEL f1 ========
NUC1                   13C
P1               7.75 usec
PL1              -3.00 dB
SFO1       100.6228298 MHz

======== CHANNEL f2 ========
CPDPRG2            waltz16
NUC2                    1H
PCPD2           80.00 usec
PL2              -6.00 dB
PL12             17.00 dB
SFO2       400.1328009 MHz

F2 - Processing parameters
SI                   32768
SF         100.6127584 MHz
WDW                     EM
SSB                      0
LB                 1.00 Hz
GB                       0
PC                    1.40
```

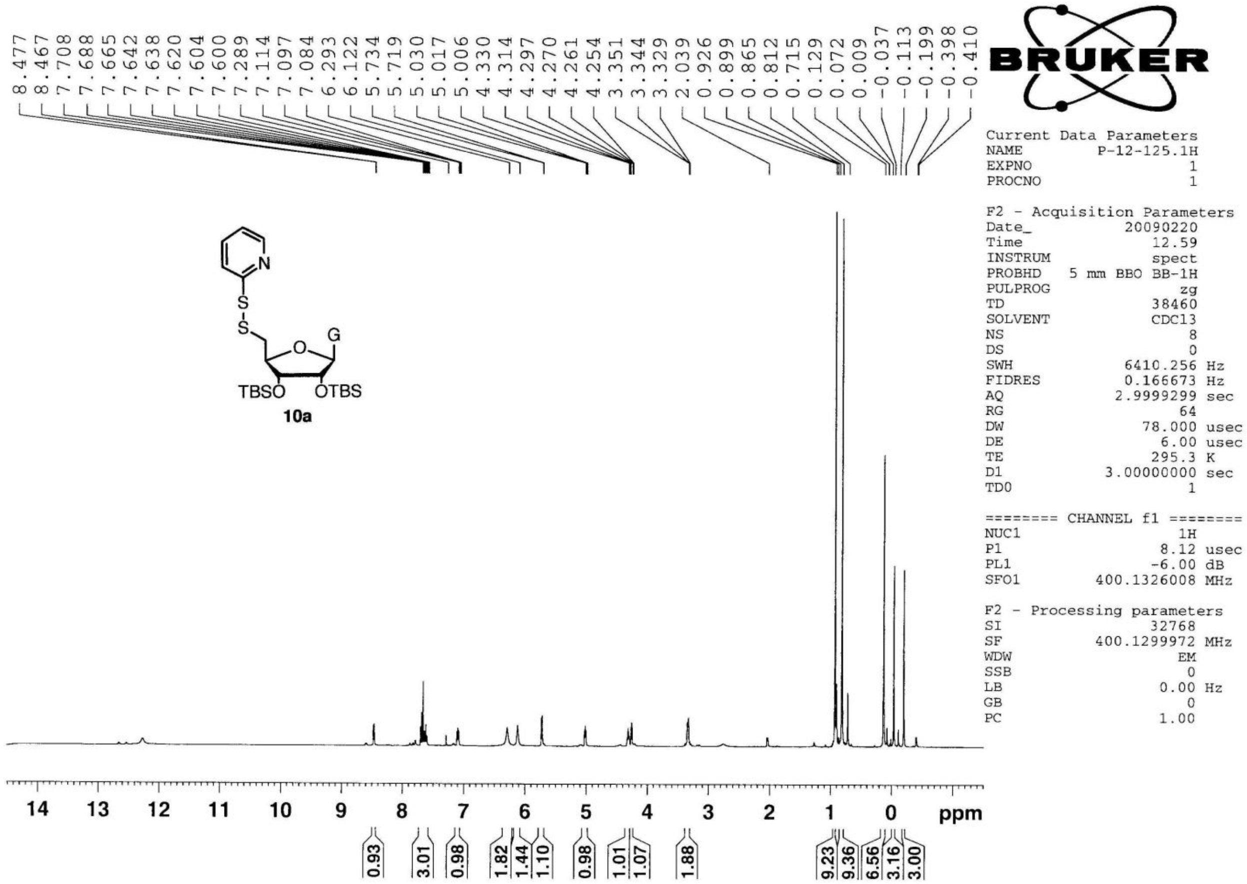

8.477
8.467
7.708
7.688
7.665
7.642
7.638
7.620
7.604
7.600
7.289
7.114
7.097
7.084
6.293
6.122
5.734
5.719
5.030
5.017
5.006
4.330
4.314
4.297
4.270
4.261
4.254
3.351
3.344
3.329
2.039
0.926
0.899
0.865
0.812
0.715
0.129
0.072
0.009
-0.037
-0.113
-0.199
-0.398
-0.410
BRUKER
Current Data Parameters
NAME P-12-125.1H
EXPNO 1
PROCNO 1
F2 - Acquisition Parameters
Date_ 20090220
Time 12.59
INSTRUM spect
PROBHD 5 mm BBO BB-1H
PULPROG zg
TD 38460
SOLVENT CDCl3
NS 8
DS 0
SWH 6410.256 Hz
FIDRES 0.166673 Hz
AQ 2.9999299 sec
RG 64
DW 78.000 usec
DE 6.00 usec
TE 295.3 K
D1 3.00000000 sec
TD0 1
======== CHANNEL f1 ========
NUC1 1H
P1 8.12 usec
PL1 -6.00 dB
SFO1 400.1326008 MHz
F2 - Processing parameters
SI 32768
SF 400.1299972 MHz
WDW EM
SSB 0
LB 0.00 Hz
GB 0
PC 1.00
N
S
S
O
G
TBSO
OTBS
10a
14 13 12 11 10 9 8 7 6 5 4 3 2 1 0 ppm
0.93
3.01
0.98
1.82
1.44
1.10
0.98
1.01
1.07
1.88
9.23
9.36
6.56
3.16
3.00

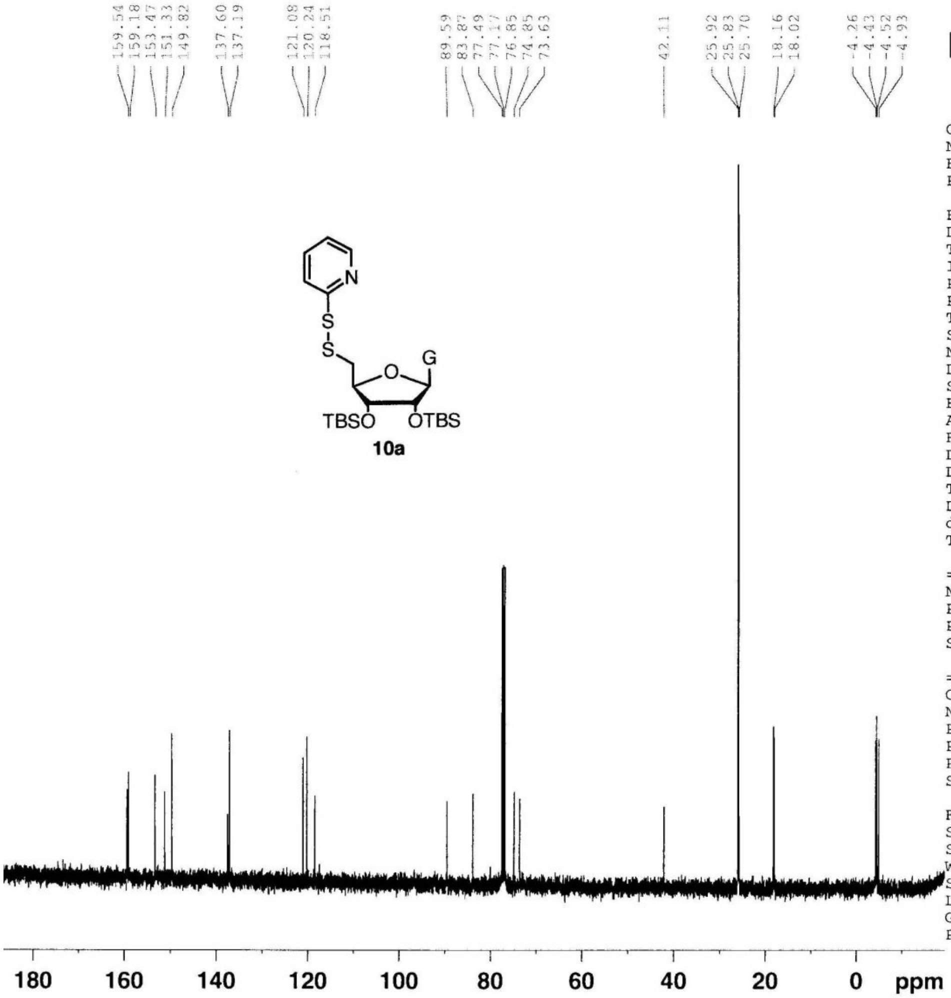

10a

BRUKER

Current Data Parameters
NAME P-12-125.13C
EXPNO 2
PROCNO 1

F2 - Acquisition Parameters
Date_ 20090220
Time 13.06
INSTRUM spect
PROBHD 5 mm BBO BB-1H
PULPROG zgdc
TD 144228
SOLVENT CDCl3
NS 44
DS 0
SWH 24038.461 Hz
FIDRES 0.166670 Hz
AQ 2.9999924 sec
RG 1149.4
DW 20.800 usec
DE 6.00 usec
TE 295.8 K
D1 3.00000000 sec
d11 0.03000000 sec
TD0 1

======== CHANNEL f1 ========
NUC1 13C
P1 7.75 usec
PL1 -3.00 dB
SFO1 100.6228298 MHz

======== CHANNEL f2 ========
CPDPRG2 waltz16
NUC2 1H
PCPD2 80.00 usec
PL2 -6.00 dB
PL12 17.00 dB
SFO2 400.1328009 MHz

F2 - Processing parameters
SI 32768
SF 100.6127591 MHz
WDW EM
SSB 0
LB 1.00 Hz
GB 0
PC 1.40

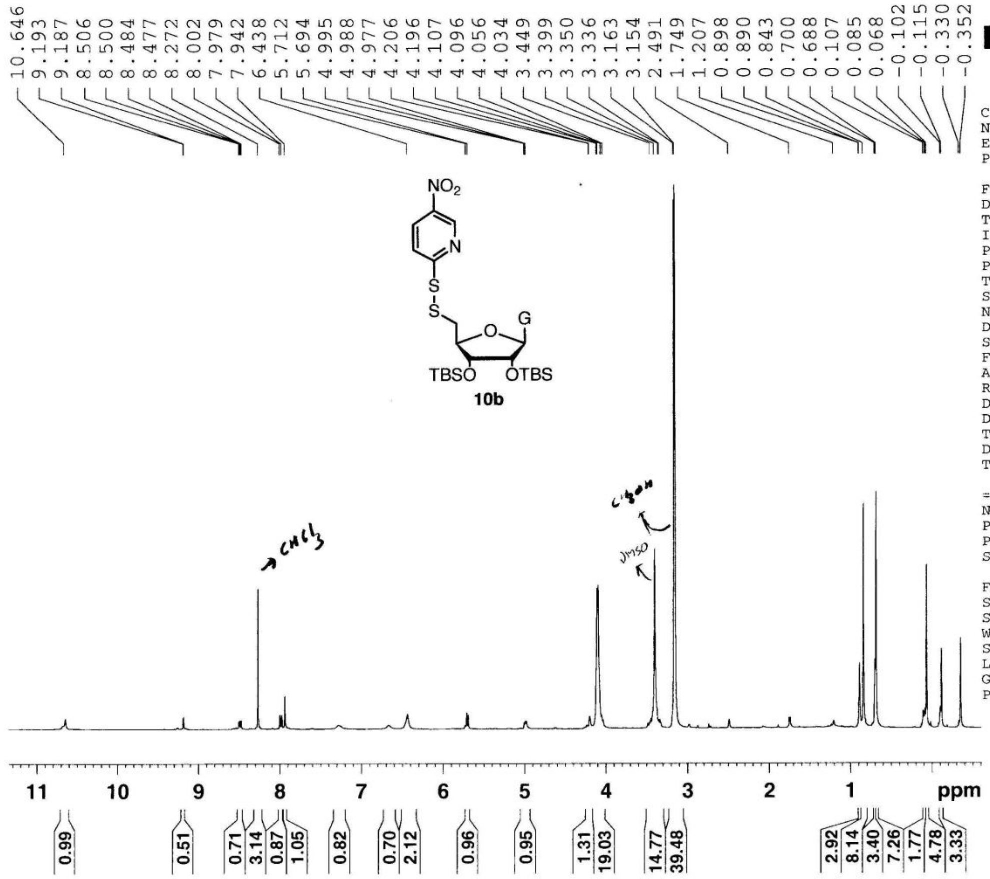

Current Data Parameters
NAME P-14-036.1H
EXPNO 1
PROCNO 1

F2 - Acquisition Parameters
Date_ 20090423
Time 15.03
INSTRUM spect
PROBHD 5 mm BBO BB-1H
PULPROG zg
TD 38460
SOLVENT DMSO
NS 12
DS 0
SWH 6410.256 Hz
FIDRES 0.166673 Hz
AQ 2.9999299 sec
RG 35.9
DW 78.000 usec
DE 6.00 usec
TE 295.6 K
D1 3.00000000 sec
TD0 1

======== CHANNEL f1 ========
NUC1 1H
P1 8.12 usec
PL1 -6.00 dB
SFO1 400.1326008 MHz

F2 - Processing parameters
SI 32768
SF 400.1300050 MHz
WDW EM
SSB 0
LB 0.00 Hz
GB 0
PC 1.00

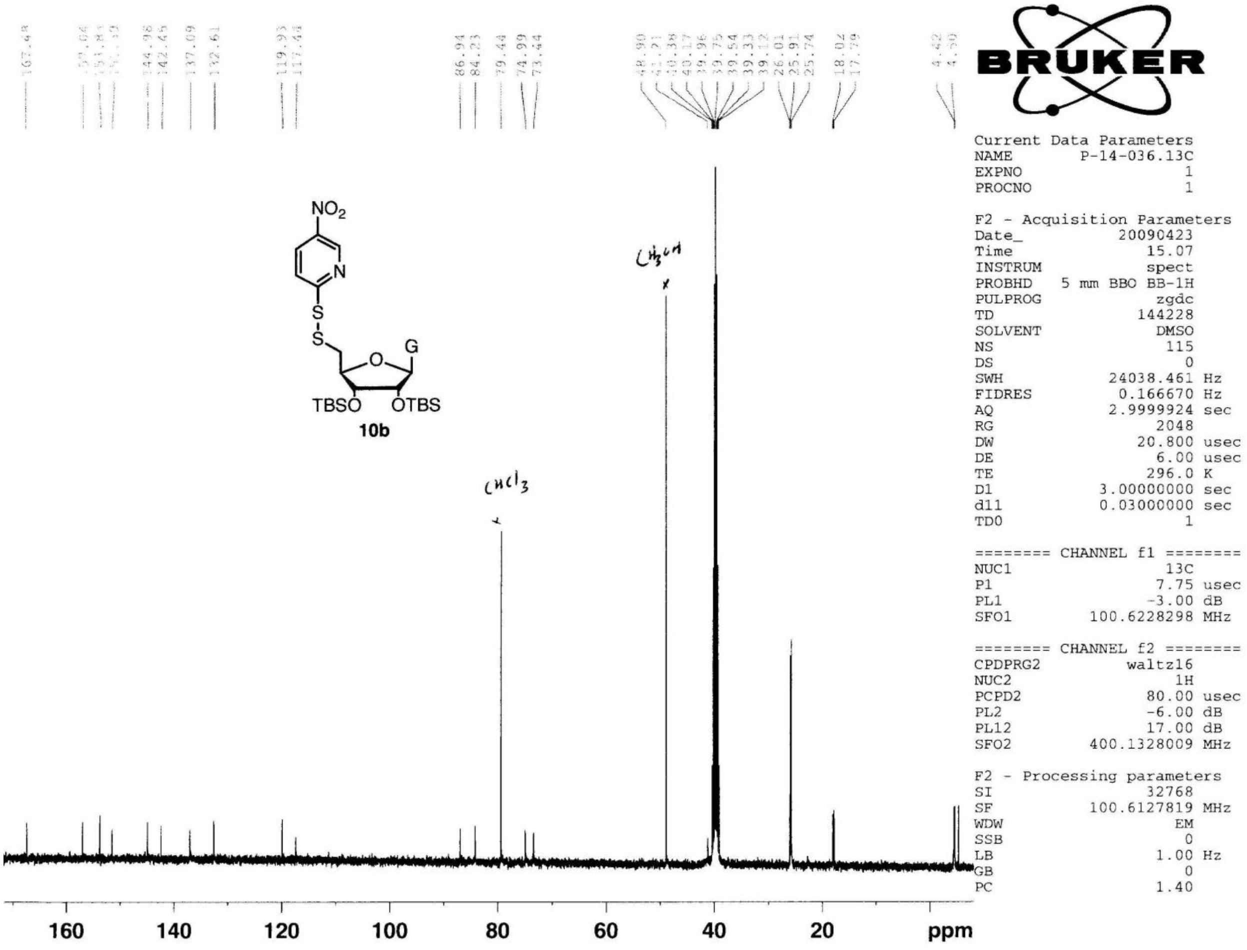

BRUKER
NO2
N
S
S
O
G
TBSO
OTBS
10b
CH3OH
CHCl3
160
140
120
100
80
60
40
20
ppm
Current Data Parameters
NAME P-14-036.13C
EXPNO 1
PROCNO 1
F2 - Acquisition Parameters
Date_ 20090423
Time 15.07
INSTRUM spect
PROBHD 5 mm BBO BB-1H
PULPROG zgdc
TD 144228
SOLVENT DMSO
NS 115
DS 0
SWH 24038.461 Hz
FIDRES 0.166670 Hz
AQ 2.9999924 sec
RG 2048
DW 20.800 usec
DE 6.00 usec
TE 296.0 K
D1 3.00000000 sec
d11 0.03000000 sec
TD0 1
======== CHANNEL f1 ========
NUC1 13C
P1 7.75 usec
PL1 -3.00 dB
SFO1 100.6228298 MHz
======== CHANNEL f2 ========
CPDPRG2 waltz16
NUC2 1H
PCPD2 80.00 usec
PL2 -6.00 dB
PL12 17.00 dB
SFO2 400.1328009 MHz
F2 - Processing parameters
SI 32768
SF 100.6127819 MHz
WDW EM
SSB 0
LB 1.00 Hz
GB 0
PC 1.40

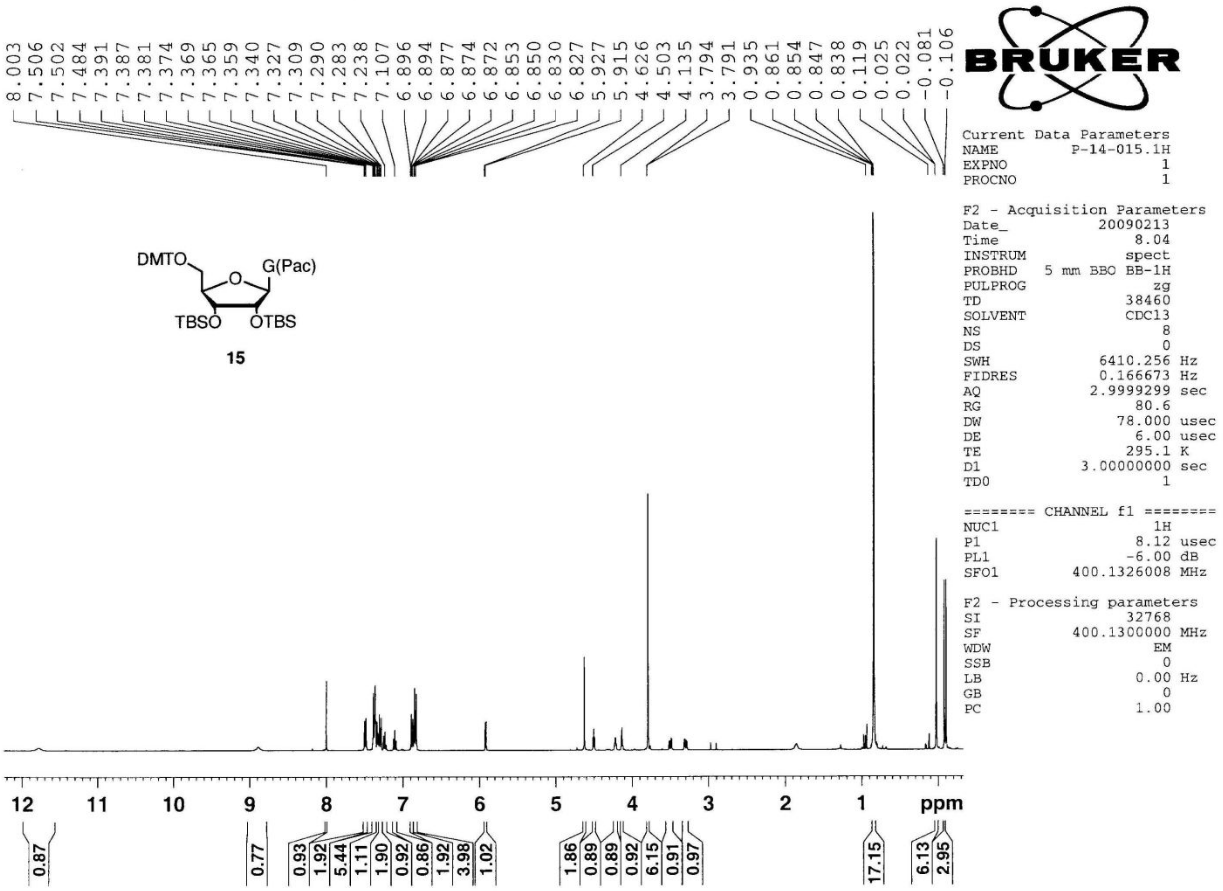
DMTO
G(Pac)
TBSO
OTBS
15
Current Data Parameters
NAME P-14-015.1H
EXPNO 1
PROCNO 1
F2 - Acquisition Parameters
Date_ 20090213
Time 8.04
INSTRUM spect
PROBHD 5 mm BBO BB-1H
PULPROG zg
TD 38460
SOLVENT CDC13
NS 8
DS 0
SWH 6410.256 Hz
FIDRES 0.166673 Hz
AQ 2.9999299 sec
RG 80.6
DW 78.000 usec
DE 6.00 usec
TE 295.1 K
D1 3.00000000 sec
TD0 1
======== CHANNEL f1 ========
NUC1 1H
P1 8.12 usec
PL1 -6.00 dB
SFO1 400.1326008 MHz
F2 - Processing parameters
SI 32768
SF 400.1300000 MHz
WDW EM
SSB 0
LB 0.00 Hz
GB 0
PC 1.00
ppm

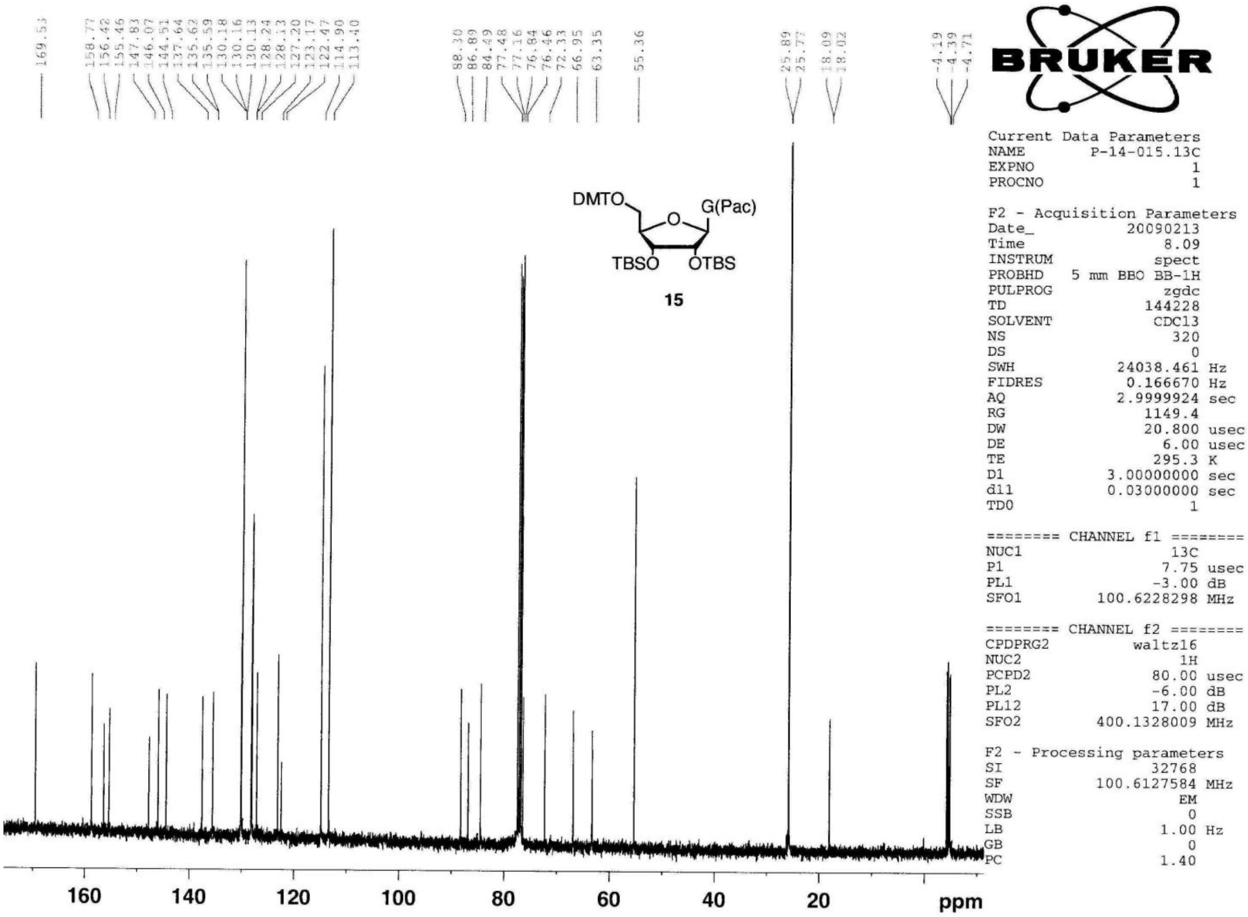
169.53
158.77
156.42
155.46
147.83
146.07
144.51
137.64
135.62
135.59
130.18
130.16
130.13
128.24
128.13
127.20
123.17
122.47
114.90
113.40
88.30
86.89
84.49
77.48
77.16
76.84
76.46
72.33
66.95
63.35
55.36
25.89
25.77
18.09
18.02
-4.19
-4.39
-4.71
DMTO
G(Pac)
O
TBSO
OTBS
15
160
140
120
100
80
60
40
20
ppm
BRUKER
Current Data Parameters
NAME P-14-015.13C
EXPNO 1
PROCNO 1
F2 - Acquisition Parameters
Date_ 20090213
Time 8.09
INSTRUM spect
PROBHD 5 mm BBO BB-1H
PULPROG zgdc
TD 144228
SOLVENT CDCl3
NS 320
DS 0
SWH 24038.461 Hz
FIDRES 0.166670 Hz
AQ 2.9999924 sec
RG 1149.4
DW 20.800 usec
DE 6.00 usec
TE 295.3 K
D1 3.00000000 sec
d11 0.03000000 sec
TD0 1
======== CHANNEL f1 ========
NUC1 13C
P1 7.75 usec
PL1 -3.00 dB
SFO1 100.6228298 MHz
======== CHANNEL f2 ========
CPDPRG2 waltz16
NUC2 1H
PCPD2 80.00 usec
PL2 -6.00 dB
PL12 17.00 dB
SFO2 400.1328009 MHz
F2 - Processing parameters
SI 32768
SF 100.6127584 MHz
WDW EM
SSB 0
LB 1.00 Hz
GB 0
PC 1.40

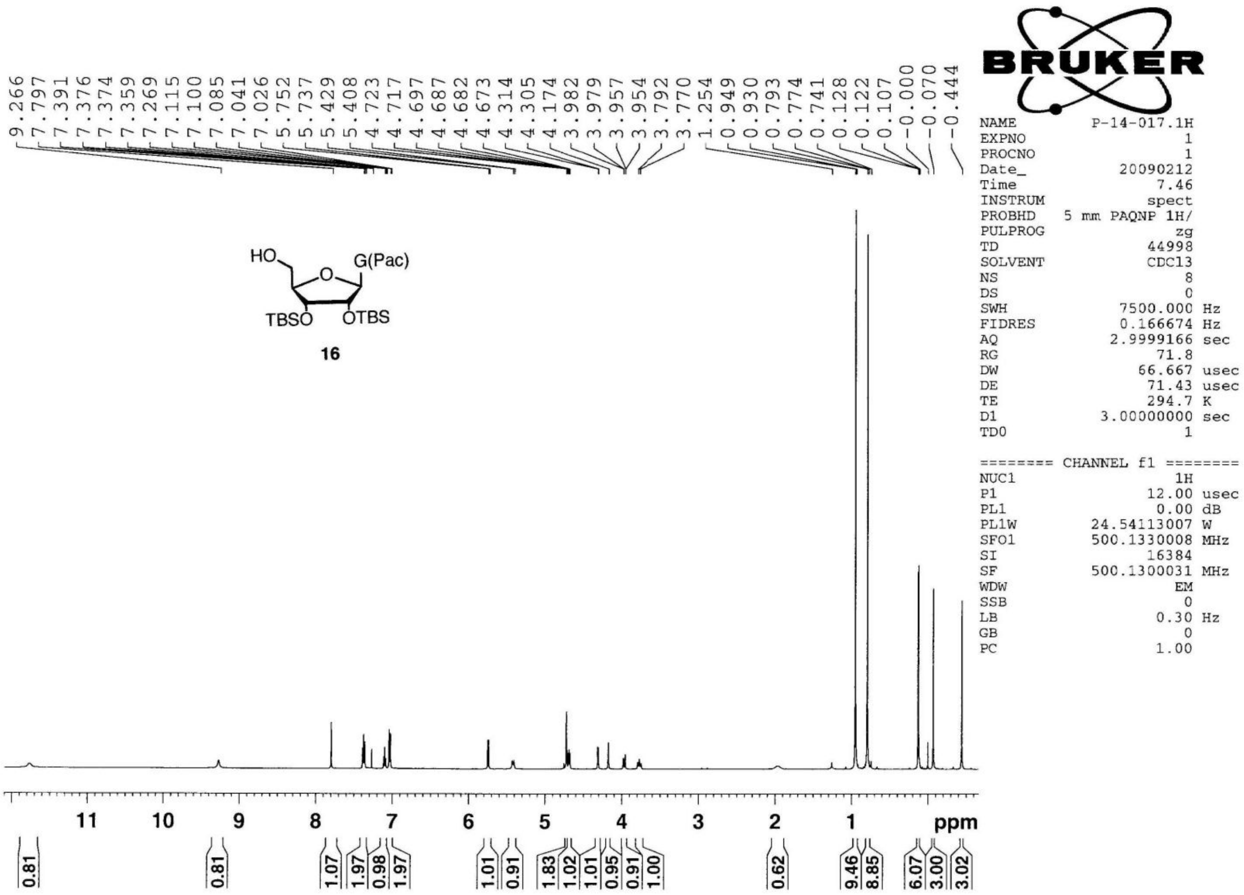
9.266 7.797 7.391 7.376 7.374 7.359 7.269 7.115 7.100 7.085 7.041 7.026 5.752 5.737 5.429 5.408 4.723 4.717 4.697 4.687 4.682 4.673 4.314 4.305 4.174 3.982 3.979 3.957 3.954 3.792 3.770 1.254 0.949 0.930 0.793 0.774 0.741 0.128 0.122 0.107 -0.000 -0.070 -0.444
HO
G(Pac)
O
TBSO
OTBS
16
BRUKER
NAME P-14-017.1H
EXPNO 1
PROCNO 1
Date_ 20090212
Time 7.46
INSTRUM spect
PROBHD 5 mm PAQNP 1H/
PULPROG zg
TD 44998
SOLVENT CDCl3
NS 8
DS 0
SWH 7500.000 Hz
FIDRES 0.166674 Hz
AQ 2.9999166 sec
RG 71.8
DW 66.667 usec
DE 71.43 usec
TE 294.7 K
D1 3.00000000 sec
TD0 1
======== CHANNEL f1 ========
NUC1 1H
P1 12.00 usec
PL1 0.00 dB
PL1W 24.54113007 W
SFO1 500.1330008 MHz
SI 16384
SF 500.1300031 MHz
WDW EM
SSB 0
LB 0.30 Hz
GB 0
PC 1.00
11 10 9 8 7 6 5 4 3 2 1 ppm
0.81 0.81 1.07 1.97 0.98 1.97 1.01 0.91 1.83 1.02 1.01 0.95 0.91 1.00 0.62 9.46 8.85 6.07 3.00 3.02

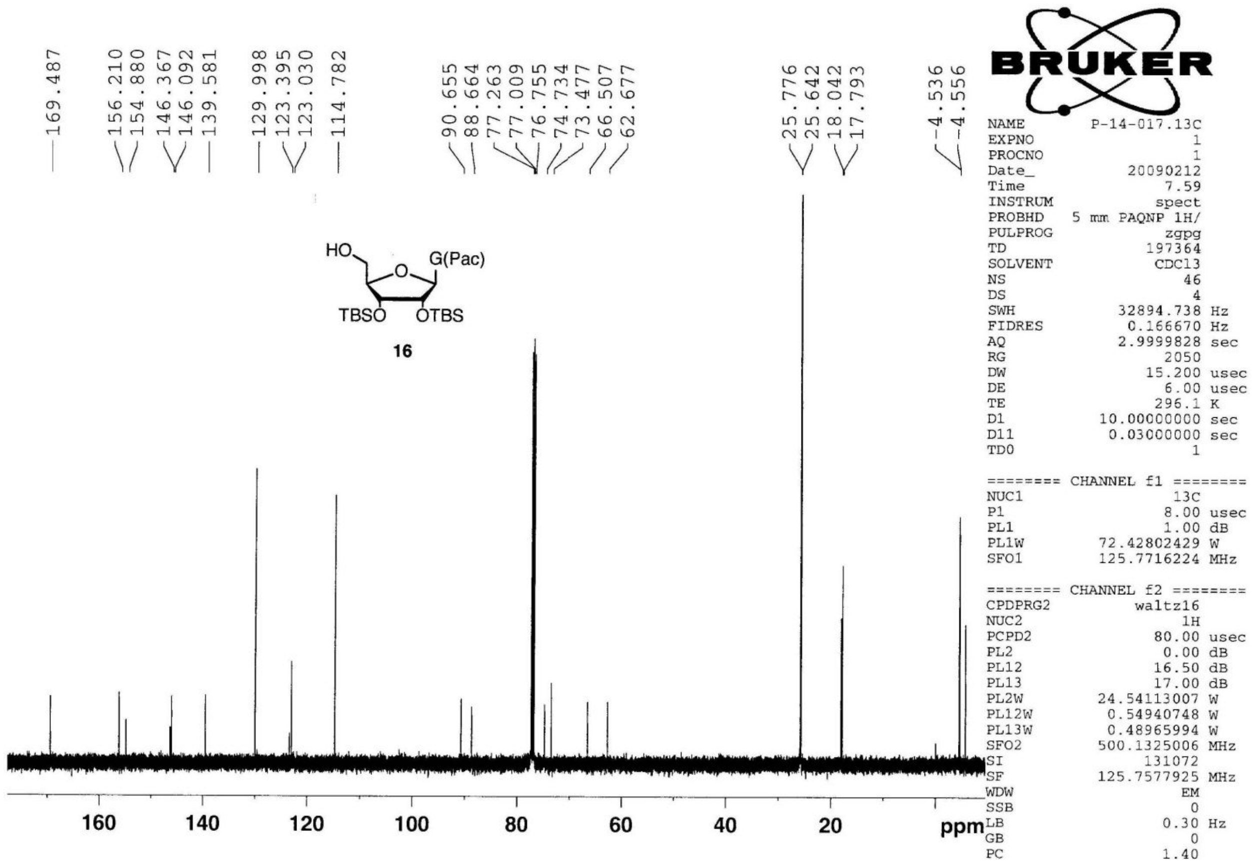
169.487
156.210
154.880
146.367
146.092
139.581
129.998
123.395
123.030
114.782
90.655
88.664
77.263
77.009
76.755
74.734
73.477
66.507
62.677
25.776
25.642
18.042
17.793
-4.536
-4.556
HO
G(Pac)
O
TBSO
OTBS
16
160
140
120
100
80
60
40
20
ppm
BRUKER
NAME P-14-017.13C
EXPNO 1
PROCNO 1
Date_ 20090212
Time 7.59
INSTRUM spect
PROBHD 5 mm PAQNP 1H/
PULPROG zgpg
TD 197364
SOLVENT CDCl3
NS 46
DS 4
SWH 32894.738 Hz
FIDRES 0.166670 Hz
AQ 2.9999828 sec
RG 2050
DW 15.200 usec
DE 6.00 usec
TE 296.1 K
D1 10.00000000 sec
D11 0.03000000 sec
TD0 1
======== CHANNEL f1 ========
NUC1 13C
P1 8.00 usec
PL1 1.00 dB
PL1W 72.42802429 W
SFO1 125.7716224 MHz
======== CHANNEL f2 ========
CPDPRG2 waltz16
NUC2 1H
PCPD2 80.00 usec
PL2 0.00 dB
PL12 16.50 dB
PL13 17.00 dB
PL2W 24.54113007 W
PL12W 0.54940748 W
PL13W 0.48965994 W
SFO2 500.1325006 MHz
SI 131072
SF 125.7577925 MHz
WDW EM
SSB 0
LB 0.30 Hz
GB 0
PC 1.40

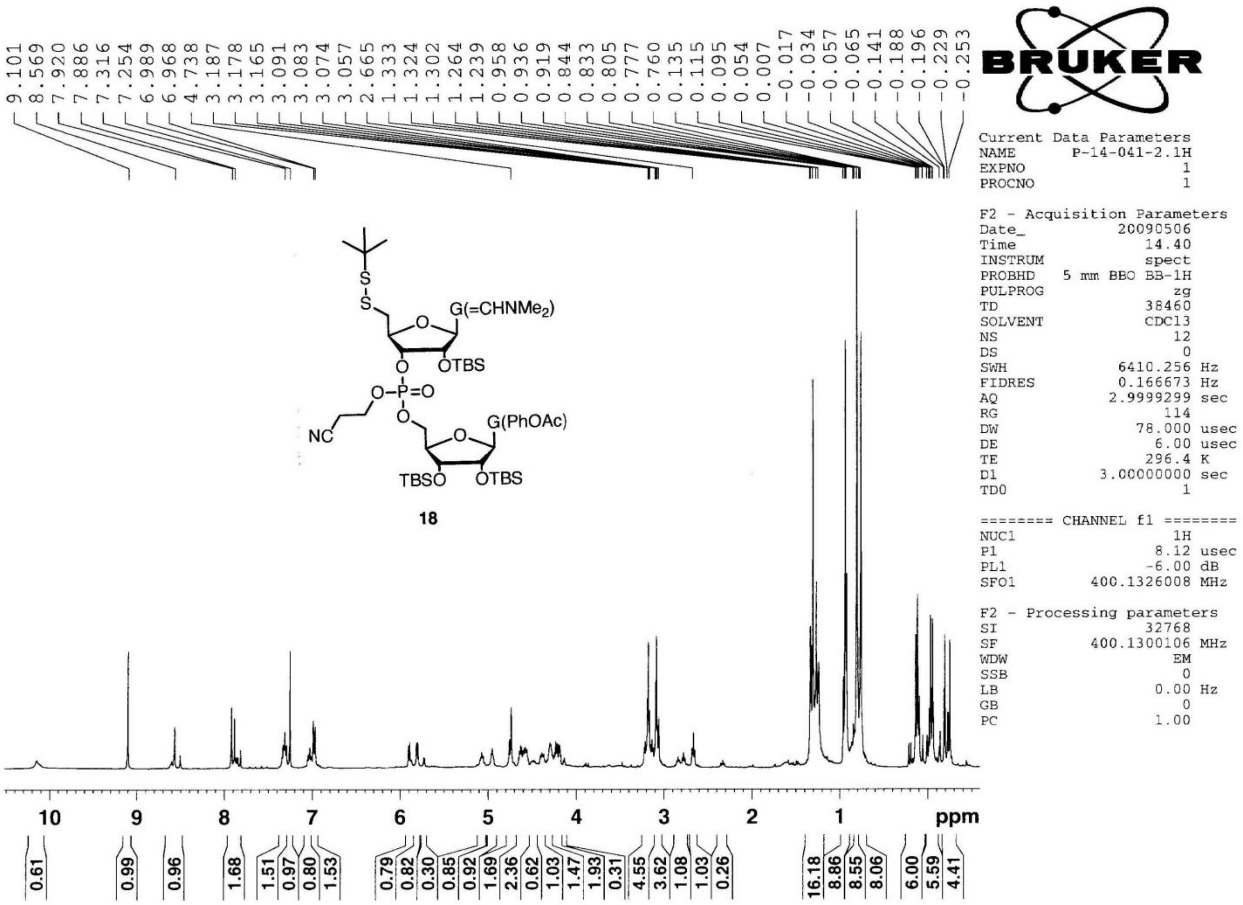
9.101
8.569
7.920
7.886
7.316
7.254
6.989
6.968
4.738
3.187
3.178
3.165
3.091
3.083
3.074
3.057
2.665
1.333
1.324
1.302
1.264
1.239
0.958
0.936
0.919
0.844
0.833
0.805
0.777
0.760
0.135
0.115
0.095
0.054
0.007
-0.017
-0.034
-0.057
-0.065
-0.141
-0.188
-0.196
-0.229
-0.253
BRUKER
Current Data Parameters
NAME P-14-041-2.1H
EXPNO 1
PROCNO 1
F2 - Acquisition Parameters
Date_ 20090506
Time 14.40
INSTRUM spect
PROBHD 5 mm BBO BB-1H
PULPROG zg
TD 38460
SOLVENT CDCl3
NS 12
DS 0
SWH 6410.256 Hz
FIDRES 0.166673 Hz
AQ 2.9999299 sec
RG 114
DW 78.000 usec
DE 6.00 usec
TE 296.4 K
D1 3.00000000 sec
TD0 1
======== CHANNEL f1 ========
NUC1 1H
P1 8.12 usec
PL1 -6.00 dB
SFO1 400.1326008 MHz
F2 - Processing parameters
SI 32768
SF 400.1300106 MHz
WDW EM
SSB 0
LB 0.00 Hz
GB 0
PC 1.00
S
S
G(=CHNMe2)
O
O
OTBS
O-P=O
O
NC
G(PhOAc)
O
TBSO
OTBS
18
10
9
8
7
6
5
4
3
2
1
ppm
0.61
0.99
0.96
1.68
1.51
0.97
0.80
1.53
0.79
0.82
0.30
0.85
0.92
1.69
2.36
0.62
1.03
1.47
1.93
0.31
4.55
3.62
1.08
1.03
0.26
16.18
8.86
8.55
8.06
6.00
5.59
4.41

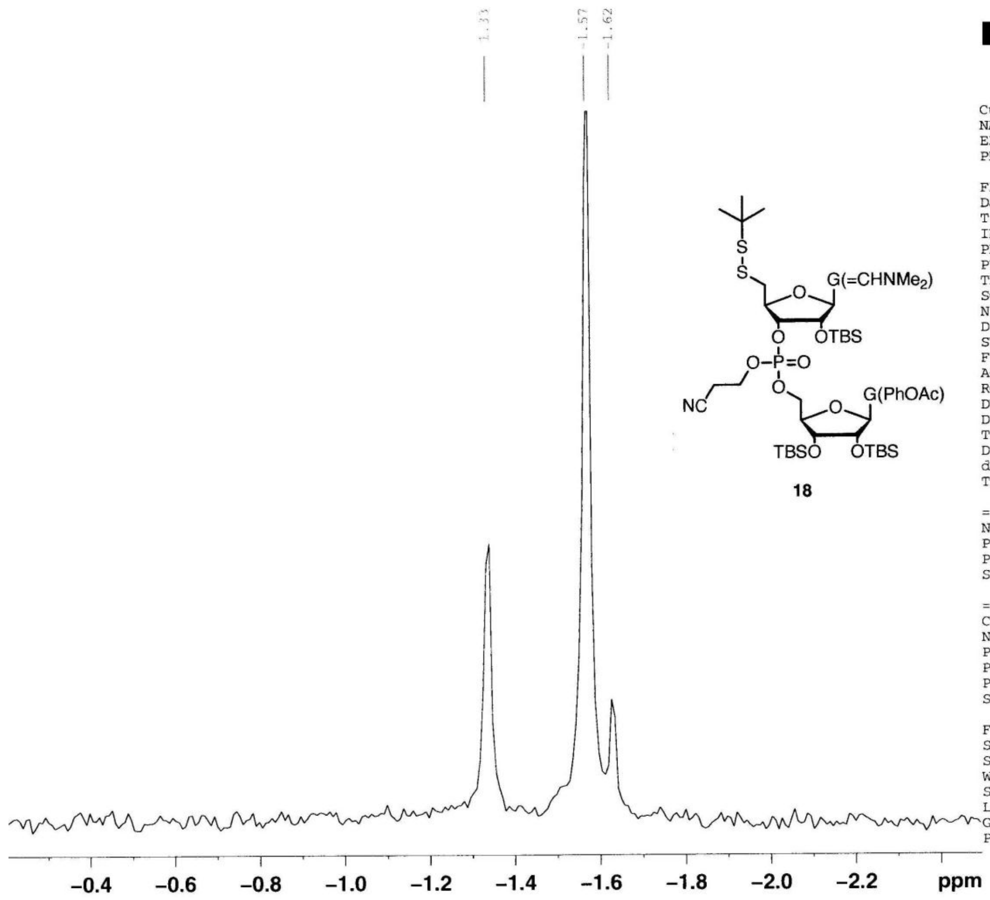


```
Current Data Parameters
NAME       P-11-041-2.31P
EXPNO                   1
PROCNO                  1

F2 - Acquisition Parameters
Date_            20090506
Time                14.34
INSTRUM             spect
PROBHD   5 mm BBO BB-1H
PULPROG              zgdc
TD                 162596
SOLVENT             CDCl3
NS                      7
DS                      0
SWH            40000.000 Hz
FIDRES          0.246009 Hz
AQ             2.0325000 sec
RG                  16384
DW                 12.500 usec
DE                   6.00 usec
TE                  296.5 K
D1            3.00000000 sec
d11           0.03000000 sec
TD0                     1

======== CHANNEL f1 ========
NUC1                  31P
P1                   8.80 usec
PL1                 -4.00 dB
SFO1         161.9869984 MHz

======== CHANNEL f2 ========
CPDPRG2           waltz16
NUC2                   1H
PCPD2               80.00 usec
PL2                 -6.00 dB
PL12                17.00 dB
SFO2         400.1328010 MHz

F2 - Processing parameters
SI                  32768
SF           161.9754607 MHz
WDW                    EM
SSB                     0
LB                   1.00 Hz
GB                      0
PC                   1.40
```

Applied Biosystems Voyager System 6187

Voyager Spec #1[BP = 1339.6, 8259]

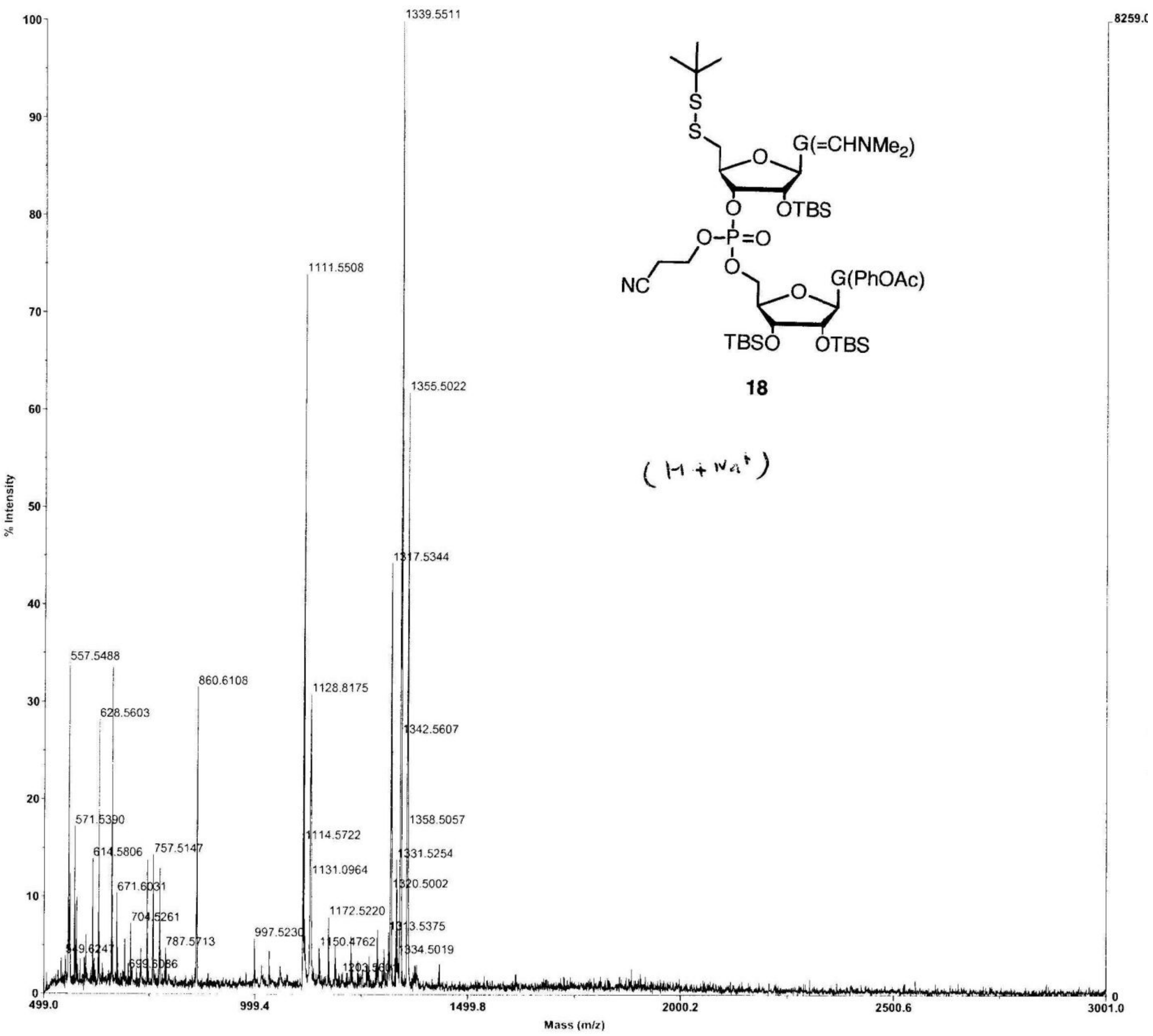

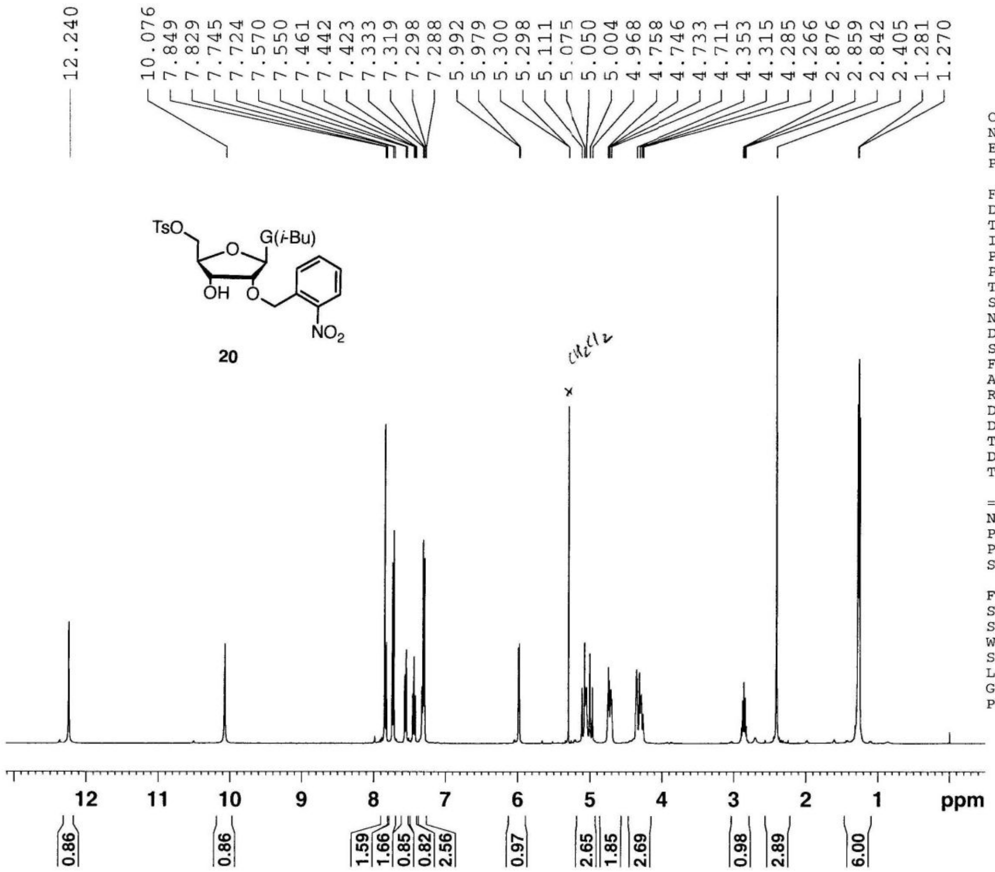


```
Current Data Parameters
NAME          P-14-053-2.1H
EXPNO                     1
PROCNO                    1

F2 - Acquisition Parameters
Date_              20120119
Time                   8.44
INSTRUM               spect
PROBHD   5 mm BBO BB-1H
PULPROG                  zg
TD                    38460
SOLVENT               CDCl3
NS                        8
DS                        0
SWH              6410.256 Hz
FIDRES           0.166673 Hz
AQ              2.9999299 sec
RG                     71.8
DW                   78.000 usec
DE                   111.43 usec
TE                    295.1 K
D1             1.00000000 sec
TD0                       1

======== CHANNEL f1 ========
NUC1                     1H
P1                     8.00 usec
PL1                   -6.00 dB
SFO1         400.1326008 MHz

F2 - Processing parameters
SI                    32768
SF           400.1299971 MHz
WDW                      EM
SSB                       0
LB                     0.00 Hz
GB                        0
PC                    10.00
```

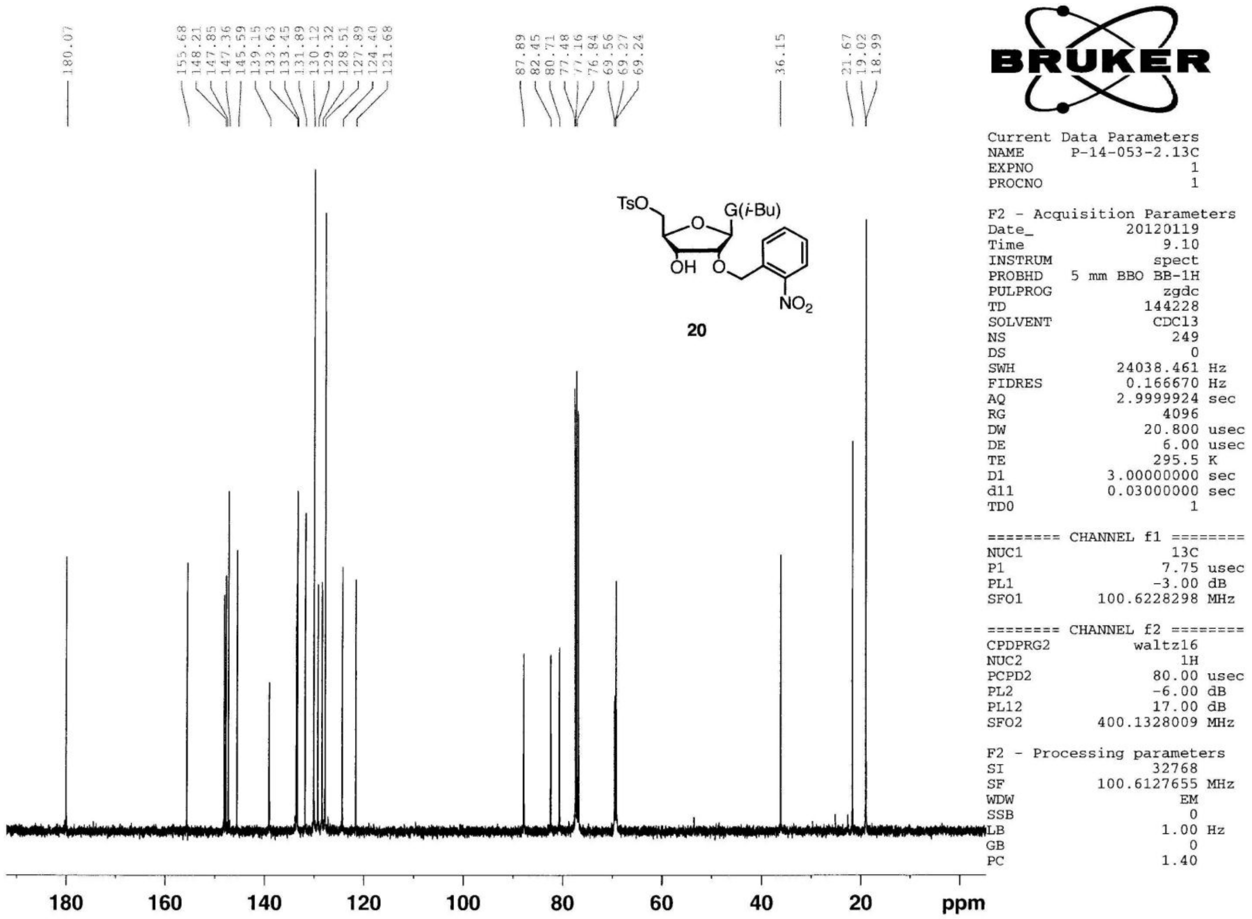
180.07
155.68
148.21
147.85
147.36
145.59
139.15
133.63
133.45
131.89
130.12
129.32
128.51
127.89
124.40
121.68
87.89
82.45
80.71
77.48
77.16
76.84
69.56
69.27
69.24
36.15
21.67
19.02
18.99
TsO
G(i-Bu)
O
OH
O
NO2
20
BRUKER
Current Data Parameters
NAME P-14-053-2.13C
EXPNO 1
PROCNO 1
F2 - Acquisition Parameters
Date_ 20120119
Time 9.10
INSTRUM spect
PROBHD 5 mm BBO BB-1H
PULPROG zgdc
TD 144228
SOLVENT CDCl3
NS 249
DS 0
SWH 24038.461 Hz
FIDRES 0.166670 Hz
AQ 2.9999924 sec
RG 4096
DW 20.800 usec
DE 6.00 usec
TE 295.5 K
D1 3.00000000 sec
d11 0.03000000 sec
TD0 1
======== CHANNEL f1 ========
NUC1 13C
P1 7.75 usec
PL1 -3.00 dB
SFO1 100.6228298 MHz
======== CHANNEL f2 ========
CPDPRG2 waltz16
NUC2 1H
PCPD2 80.00 usec
PL2 -6.00 dB
PL12 17.00 dB
SFO2 400.1328009 MHz
F2 - Processing parameters
SI 32768
SF 100.6127655 MHz
WDW EM
SSB 0
LB 1.00 Hz
GB 0
PC 1.40
180
160
140
120
100
80
60
40
20
ppm

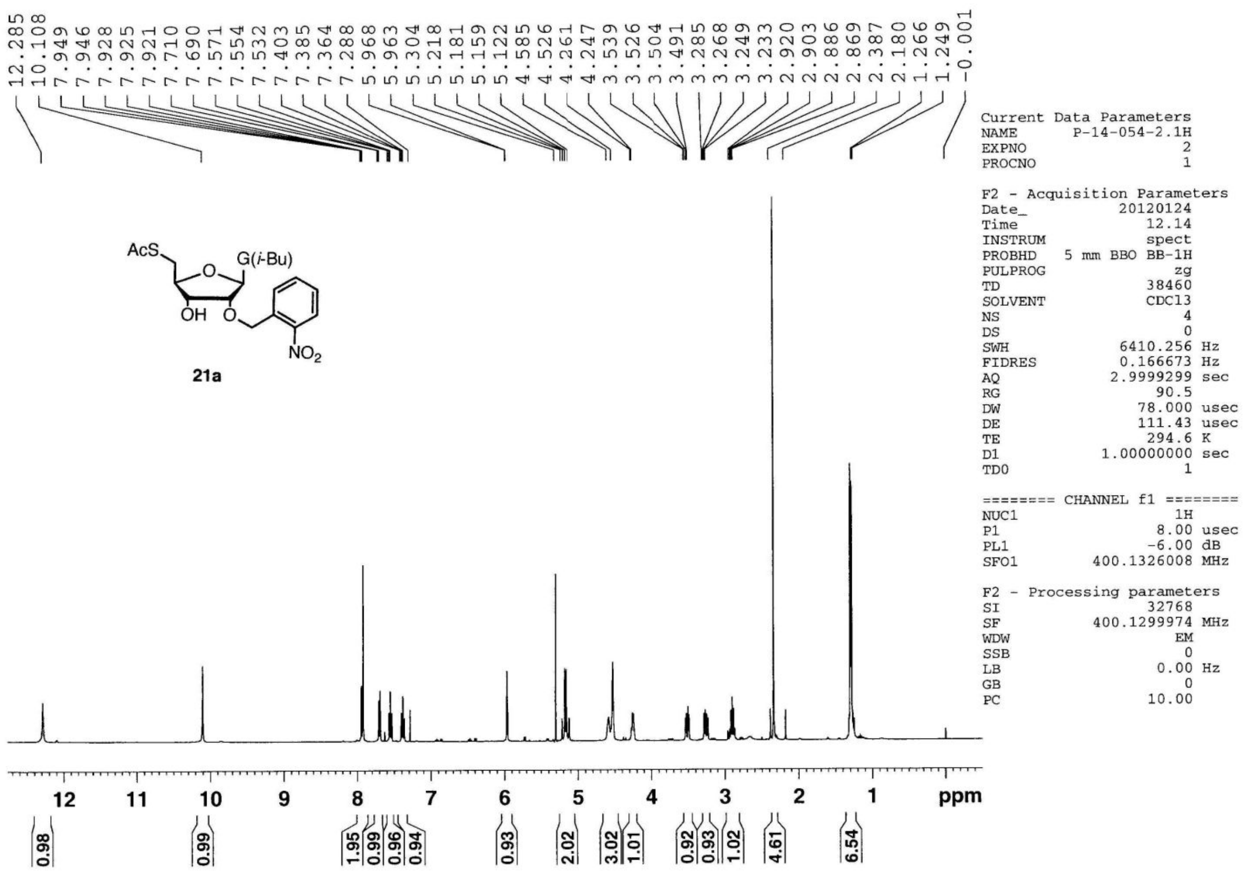
12.285
10.108
7.949
7.946
7.928
7.925
7.921
7.710
7.690
7.571
7.554
7.532
7.403
7.385
7.364
7.288
5.968
5.963
5.304
5.218
5.181
5.159
5.122
4.585
4.526
4.261
4.247
3.539
3.526
3.504
3.491
3.285
3.268
3.249
3.233
2.920
2.903
2.886
2.869
2.387
2.180
1.266
1.249
-0.001
AcS
G(i-Bu)
OH
NO2
21a
Current Data Parameters
NAME P-14-054-2.1H
EXPNO 2
PROCNO 1
F2 - Acquisition Parameters
Date_ 20120124
Time 12.14
INSTRUM spect
PROBHD 5 mm BBO BB-1H
PULPROG zg
TD 38460
SOLVENT CDCl3
NS 4
DS 0
SWH 6410.256 Hz
FIDRES 0.166673 Hz
AQ 2.9999299 sec
RG 90.5
DW 78.000 usec
DE 111.43 usec
TE 294.6 K
D1 1.00000000 sec
TD0 1
======== CHANNEL f1 ========
NUC1 1H
P1 8.00 usec
PL1 -6.00 dB
SFO1 400.1326008 MHz
F2 - Processing parameters
SI 32768
SF 400.1299974 MHz
WDW EM
SSB 0
LB 0.00 Hz
GB 0
PC 10.00
12 11 10 9 8 7 6 5 4 3 2 1 ppm
0.98
0.99
1.95
0.99
0.96
0.94
0.93
2.02
3.02
1.01
0.92
0.93
1.02
4.61
6.54

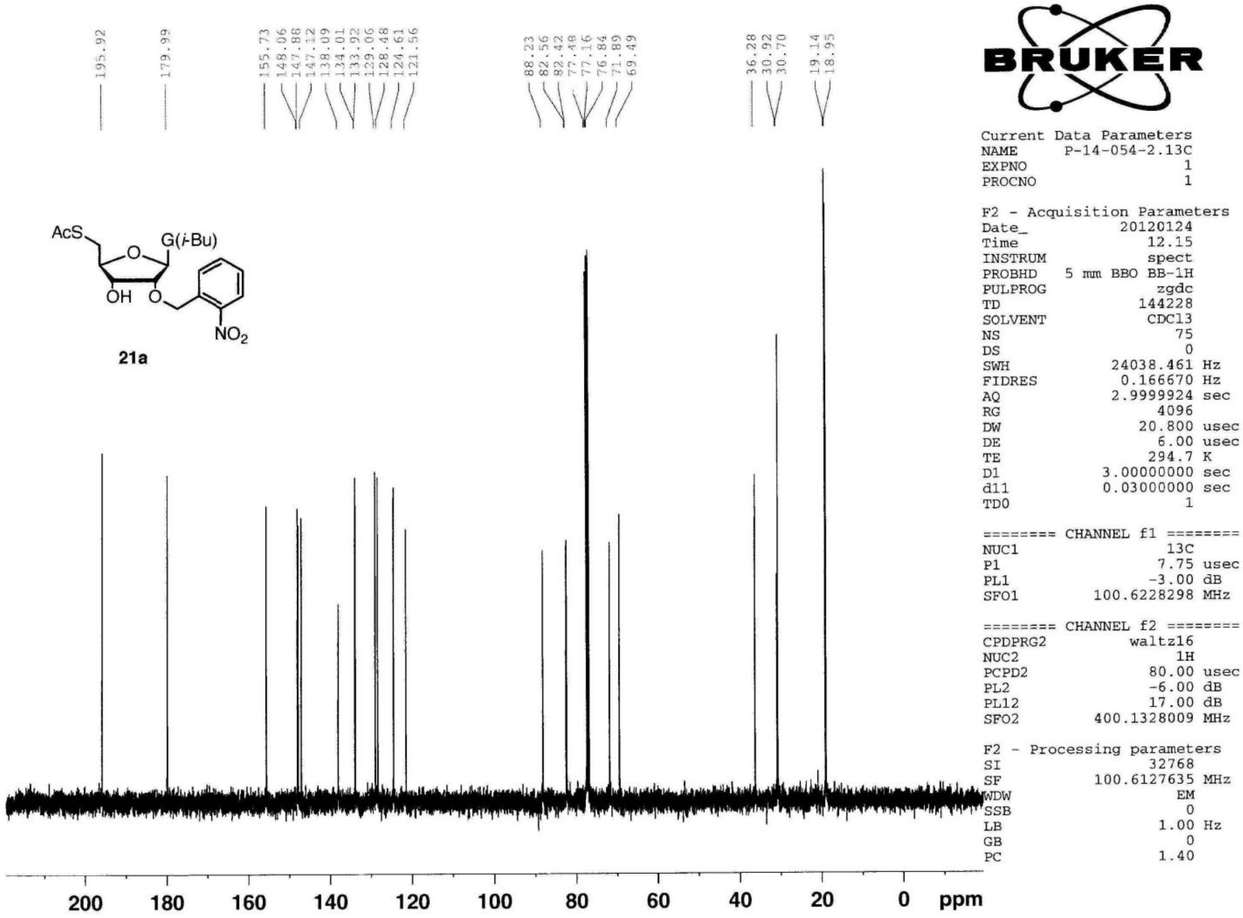
195.92
179.99
155.73
148.06
147.88
147.12
138.09
134.01
133.92
129.06
128.48
124.61
121.56
88.23
82.56
82.42
77.48
77.16
76.84
71.89
69.49
36.28
30.92
30.70
19.14
18.95
AcS
G(i-Bu)
O
OH
O
NO2
21a
BRUKER
Current Data Parameters
NAME P-14-054-2.13C
EXPNO 1
PROCNO 1
F2 - Acquisition Parameters
Date_ 20120124
Time 12.15
INSTRUM spect
PROBHD 5 mm BBO BB-1H
PULPROG zgdc
TD 144228
SOLVENT CDCl3
NS 75
DS 0
SWH 24038.461 Hz
FIDRES 0.166670 Hz
AQ 2.9999924 sec
RG 4096
DW 20.800 usec
DE 6.00 usec
TE 294.7 K
D1 3.00000000 sec
d11 0.03000000 sec
TD0 1
======== CHANNEL f1 ========
NUC1 13C
P1 7.75 usec
PL1 -3.00 dB
SFO1 100.6228298 MHz
======== CHANNEL f2 ========
CPDPRG2 waltz16
NUC2 1H
PCPD2 80.00 usec
PL2 -6.00 dB
PL12 17.00 dB
SFO2 400.1328009 MHz
F2 - Processing parameters
SI 32768
SF 100.6127635 MHz
WDW EM
SSB 0
LB 1.00 Hz
GB 0
PC 1.40
200
180
160
140
120
100
80
60
40
20
0
ppm

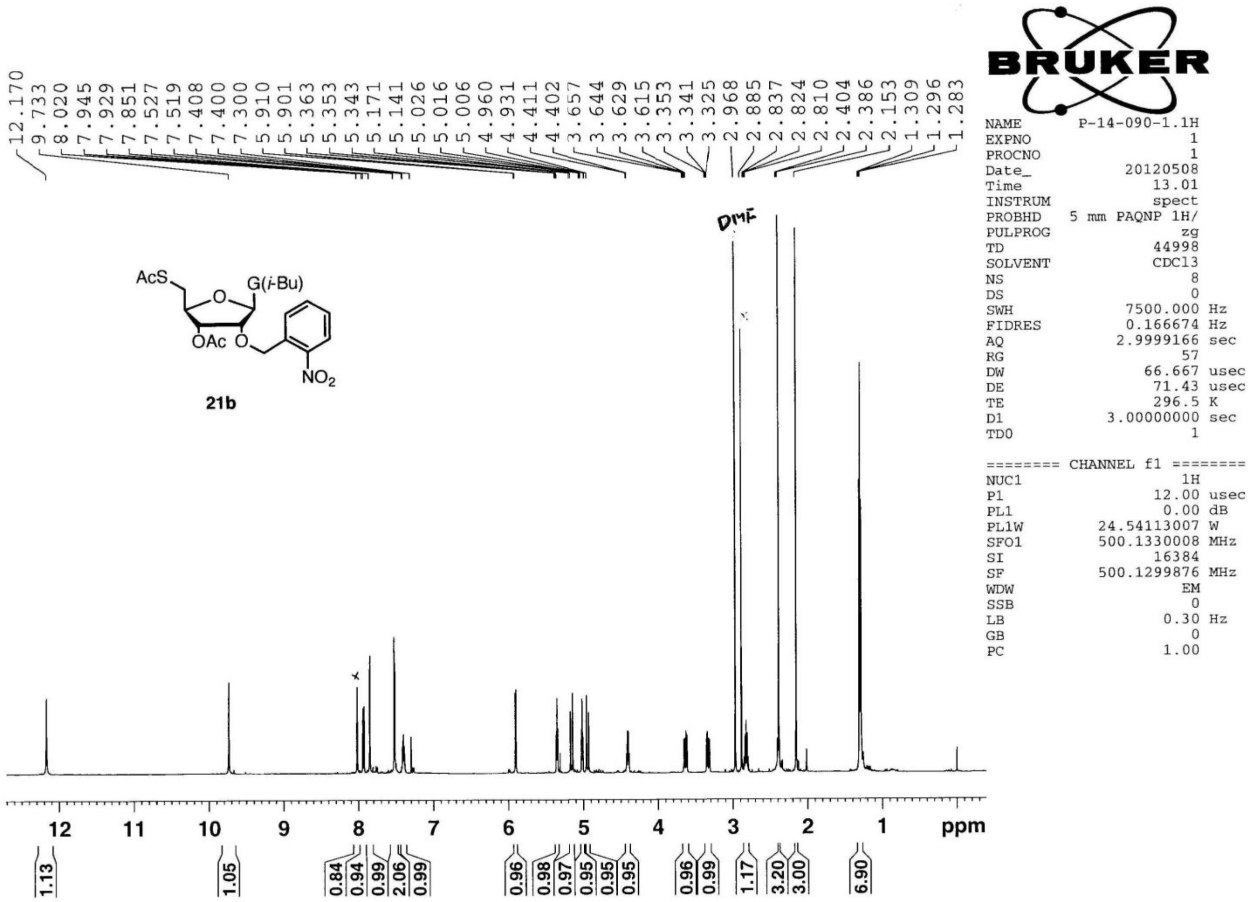
12.170
9.733
8.020
7.945
7.929
7.851
7.527
7.519
7.408
7.400
7.300
5.910
5.901
5.363
5.353
5.343
5.171
5.141
5.026
5.016
5.006
4.960
4.931
4.411
4.402
3.657
3.644
3.629
3.615
3.353
3.341
3.325
2.968
2.885
2.837
2.824
2.810
2.404
2.386
2.153
1.309
1.296
1.283
DMF
AcS
G(i-Bu)
OAc
NO2
21b
NAME P-14-090-1.1H
EXPNO 1
PROCNO 1
Date_ 20120508
Time 13.01
INSTRUM spect
PROBHD 5 mm PAQNP 1H/
PULPROG zg
TD 44998
SOLVENT CDCl3
NS 8
DS 0
SWH 7500.000 Hz
FIDRES 0.166674 Hz
AQ 2.9999166 sec
RG 57
DW 66.667 usec
DE 71.43 usec
TE 296.5 K
D1 3.00000000 sec
TD0 1
======== CHANNEL f1 ========
NUC1 1H
P1 12.00 usec
PL1 0.00 dB
PL1W 24.54113007 W
SFO1 500.1330008 MHz
SI 16384
SF 500.1299876 MHz
WDW EM
SSB 0
LB 0.30 Hz
GB 0
PC 1.00
12
11
10
9
8
7
6
5
4
3
2
1
ppm
1.13
1.05
0.84
0.94
0.99
2.06
0.99
0.96
0.98
0.97
0.95
0.95
0.95
0.96
0.99
1.17
3.20
3.00
6.90

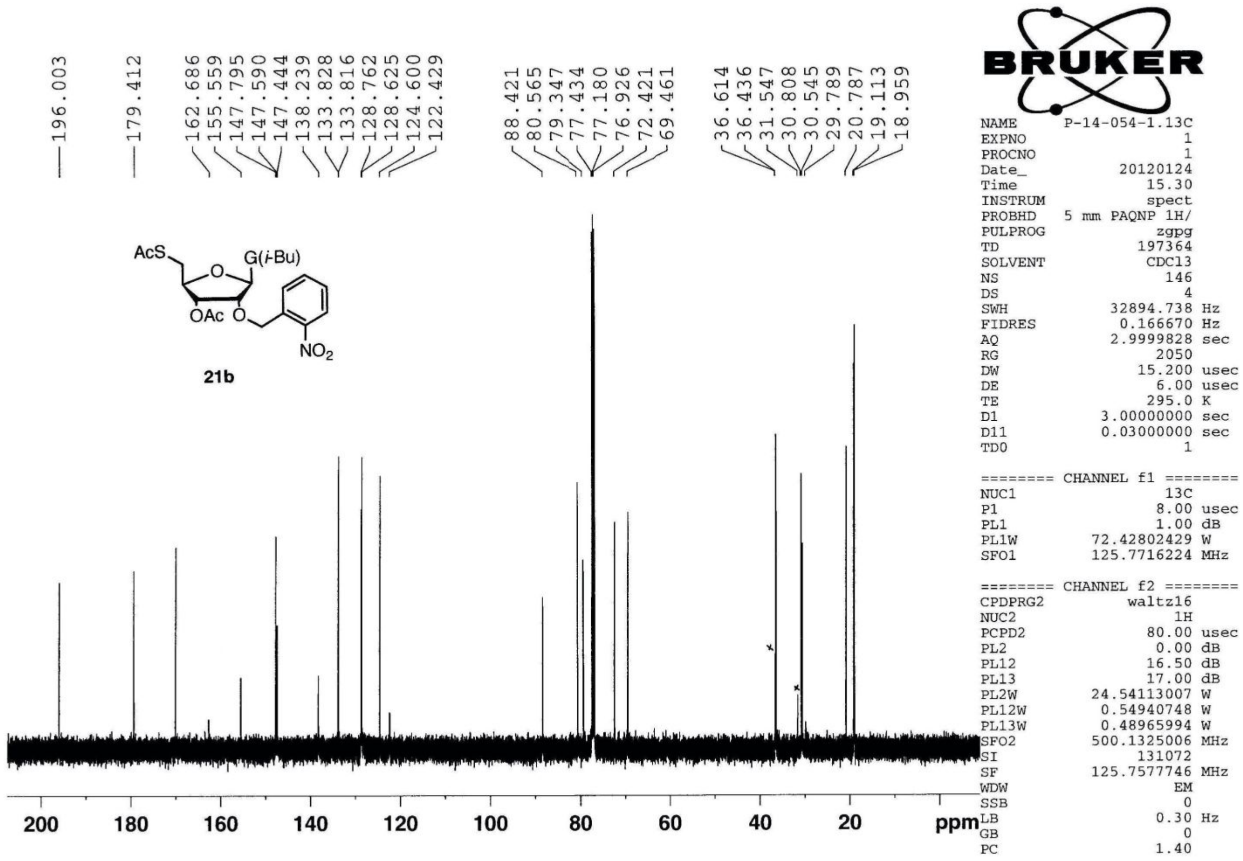
196.003
179.412
162.686
155.559
147.795
147.590
147.444
138.239
133.828
133.816
128.762
128.625
124.600
122.429
88.421
80.565
79.347
77.434
77.180
76.926
72.421
69.461
36.614
36.436
31.547
30.808
30.545
29.789
20.787
19.113
18.959
AcS
G(i-Bu)
O
OAc
NO2
21b
BRUKER
NAME P-14-054-1.13C
EXPNO 1
PROCNO 1
Date_ 20120124
Time 15.30
INSTRUM spect
PROBHD 5 mm PAQNP 1H/
PULPROG zgpg
TD 197364
SOLVENT CDCl3
NS 146
DS 4
SWH 32894.738 Hz
FIDRES 0.166670 Hz
AQ 2.9999828 sec
RG 2050
DW 15.200 usec
DE 6.00 usec
TE 295.0 K
D1 3.00000000 sec
D11 0.03000000 sec
TD0 1
======== CHANNEL f1 ========
NUC1 13C
P1 8.00 usec
PL1 1.00 dB
PL1W 72.42802429 W
SFO1 125.7716224 MHz
======== CHANNEL f2 ========
CPDPRG2 waltz16
NUC2 1H
PCPD2 80.00 usec
PL2 0.00 dB
PL12 16.50 dB
PL13 17.00 dB
PL2W 24.54113007 W
PL12W 0.54940748 W
PL13W 0.48965994 W
SFO2 500.1325006 MHz
SI 131072
SF 125.7577746 MHz
WDW EM
SSB 0
LB 0.30 Hz
GB 0
PC 1.40
200 180 160 140 120 100 80 60 40 20 ppm

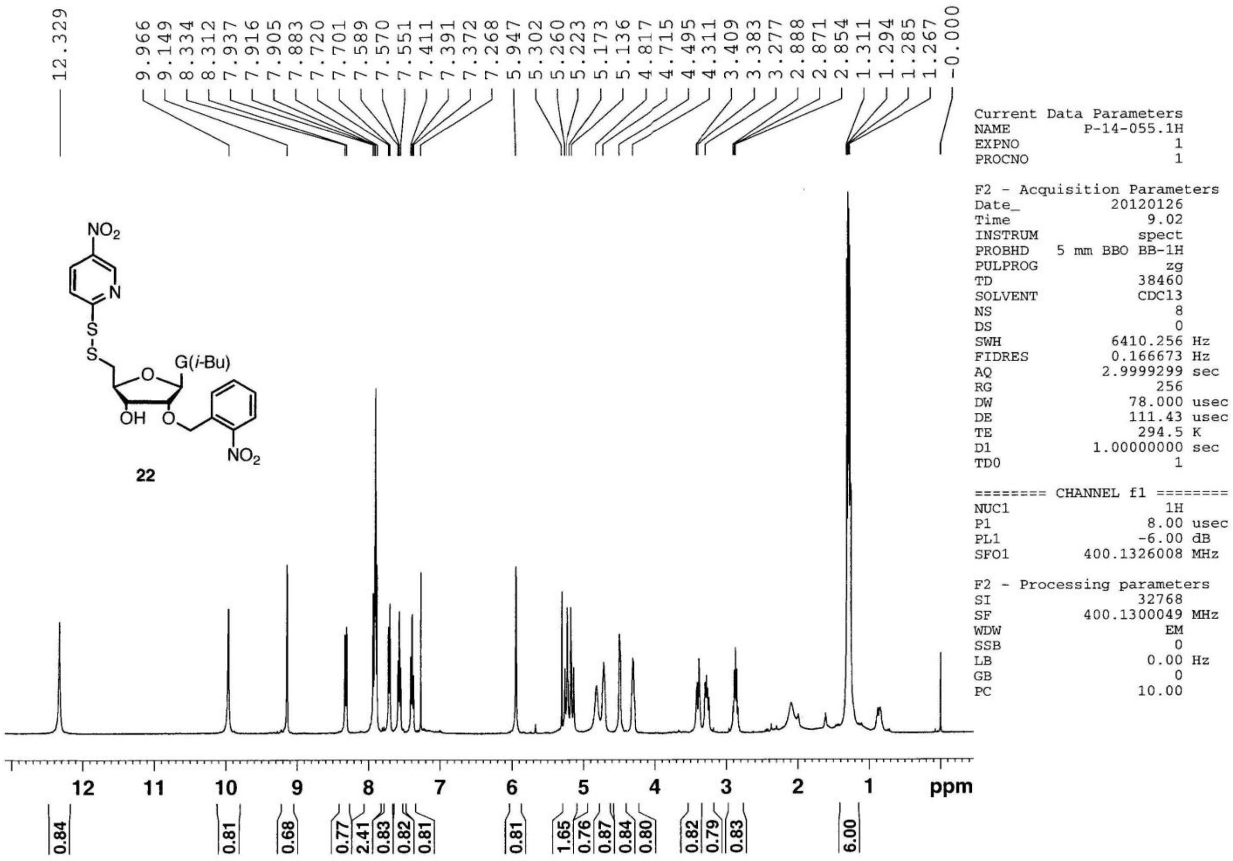
12.329
9.966 9.149 8.334 8.312 7.937 7.916 7.905 7.883 7.720 7.701 7.589 7.570 7.551 7.411 7.391 7.372 7.268 5.947 5.302 5.260 5.223 5.173 5.136 4.817 4.715 4.495 4.311 3.409 3.383 3.277 2.888 2.871 2.854 1.311 1.294 1.285 1.267 -0.000
Current Data Parameters
NAME P-14-055.1H
EXPNO 1
PROCNO 1
F2 - Acquisition Parameters
Date_ 20120126
Time 9.02
INSTRUM spect
PROBHD 5 mm BBO BB-1H
PULPROG zg
TD 38460
SOLVENT CDCl3
NS 8
DS 0
SWH 6410.256 Hz
FIDRES 0.166673 Hz
AQ 2.9999299 sec
RG 256
DW 78.000 usec
DE 111.43 usec
TE 294.5 K
D1 1.00000000 sec
TD0 1
======== CHANNEL f1 ========
NUC1 1H
P1 8.00 usec
PL1 -6.00 dB
SFO1 400.1326008 MHz
F2 - Processing parameters
SI 32768
SF 400.1300049 MHz
WDW EM
SSB 0
LB 0.00 Hz
GB 0
PC 10.00
NO2
N
S
S
O
G(i-Bu)
OH
O
NO2
22
12 11 10 9 8 7 6 5 4 3 2 1 ppm
0.84 0.81 0.68 0.77 2.41 0.83 0.82 0.81 0.81 1.65 0.76 0.87 0.84 0.80 0.82 0.79 0.83 6.00

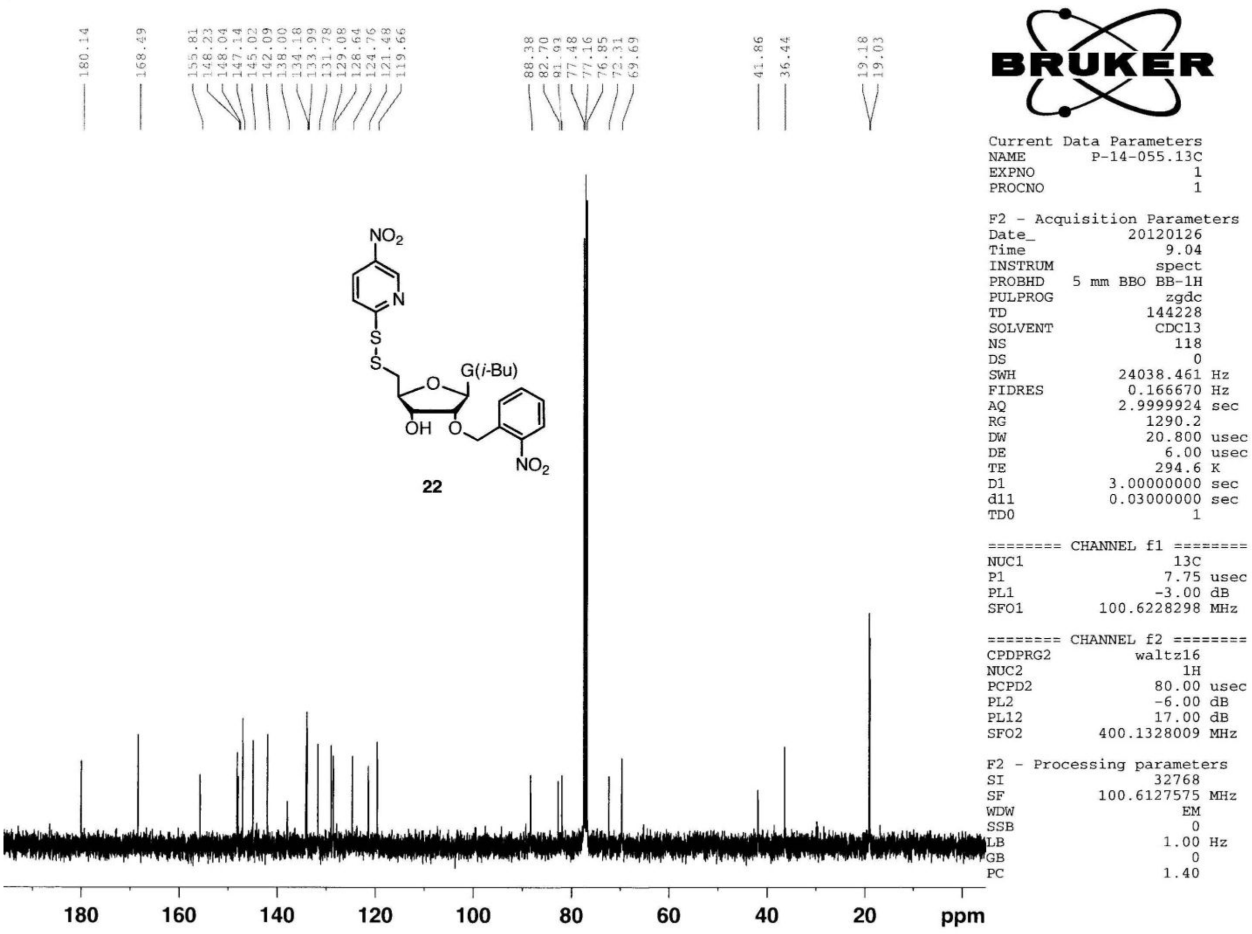
180.14
168.49
155.81
148.23
148.04
147.14
145.02
142.09
138.00
134.18
133.99
131.78
129.08
128.64
124.76
121.48
119.66
88.38
82.70
81.93
77.48
77.16
76.85
72.31
69.69
41.86
36.44
19.18
19.03
NO_2
N
S
S
O
G(i-Bu)
OH
O
NO_2
22
BRUKER
Current Data Parameters
NAME P-14-055.13C
EXPNO 1
PROCNO 1
F2 - Acquisition Parameters
Date_ 20120126
Time 9.04
INSTRUM spect
PROBHD 5 mm BBO BB-1H
PULPROG zgdc
TD 144228
SOLVENT CDCl3
NS 118
DS 0
SWH 24038.461 Hz
FIDRES 0.166670 Hz
AQ 2.9999924 sec
RG 1290.2
DW 20.800 usec
DE 6.00 usec
TE 294.6 K
D1 3.00000000 sec
d11 0.03000000 sec
TD0 1
======== CHANNEL f1 ========
NUC1 13C
P1 7.75 usec
PL1 -3.00 dB
SFO1 100.6228298 MHz
======== CHANNEL f2 ========
CPDPRG2 waltz16
NUC2 1H
PCPD2 80.00 usec
PL2 -6.00 dB
PL12 17.00 dB
SFO2 400.1328009 MHz
F2 - Processing parameters
SI 32768
SF 100.6127575 MHz
WDW EM
SSB 0
LB 1.00 Hz
GB 0
PC 1.40
180
160
140
120
100
80
60
40
20
ppm

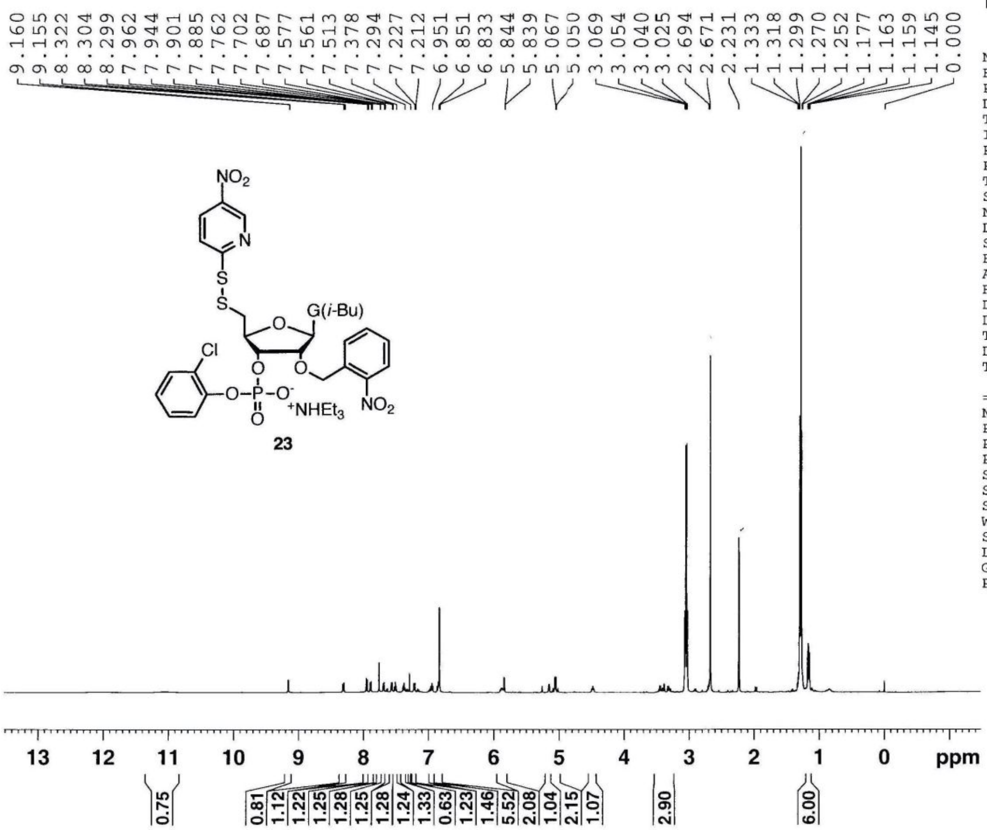

BRUKER

NAME	P-11-060.1H
EXPNO	1
PROCNO	1
Date_	20120511
Time	13.16
INSTRUM	spect
PROBHD	5 mm PAQNP 1H/
PULPROG	zg
TD	44998
SOLVENT	CDCl3
NS	8
DS	0
SWH	7500.000 Hz
FIDRES	0.166674 Hz
AQ	2.9999166 sec
RG	45.2
DW	66.667 usec
DE	71.43 usec
TE	296.5 K
D1	3.00000000 sec
TD0	1

======== CHANNEL f1 ========

NUC1	1H
P1	12.00 usec
PL1	0.00 dB
PL1W	24.54113007 W
SFO1	500.1330008 MHz
SI	16384
SF	500.1299908 MHz
WDW	EM
SSB	0
LB	0.30 Hz
GB	0
PC	1.00

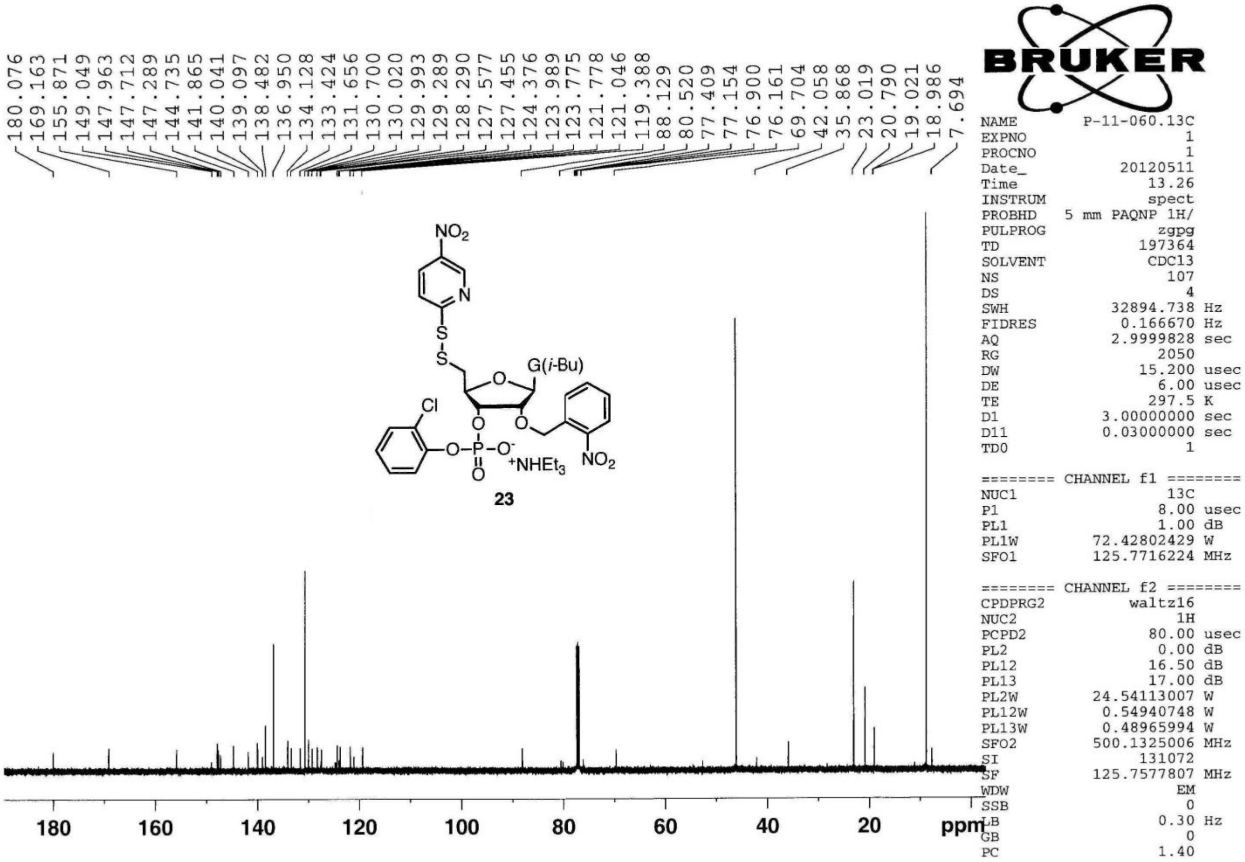
180.076
169.163
155.871
149.049
147.963
147.712
147.289
144.735
141.865
140.041
139.097
138.482
136.950
134.128
133.424
131.656
130.700
130.020
129.993
129.289
128.290
127.577
127.455
124.376
123.989
123.775
121.778
121.046
119.388
88.129
80.520
77.409
77.154
76.900
76.161
69.704
42.058
35.868
23.019
20.790
19.021
18.986
7.694
NO2
N
S
S
G(i-Bu)
O
Cl
O
O
O–P–O-
O
+NHEt3
NO2
23
180
160
140
120
100
80
60
40
20
ppm
BRUKER
NAME P-11-060.13C
EXPNO 1
PROCNO 1
Date_ 20120511
Time 13.26
INSTRUM spect
PROBHD 5 mm PAQNP 1H/
PULPROG zgpg
TD 197364
SOLVENT CDCl3
NS 107
DS 4
SWH 32894.738 Hz
FIDRES 0.166670 Hz
AQ 2.9999828 sec
RG 2050
DW 15.200 usec
DE 6.00 usec
TE 297.5 K
D1 3.00000000 sec
D11 0.03000000 sec
TD0 1
======== CHANNEL f1 ========
NUC1 13C
P1 8.00 usec
PL1 1.00 dB
PL1W 72.42802429 W
SFO1 125.7716224 MHz
======== CHANNEL f2 ========
CPDPRG2 waltz16
NUC2 1H
PCPD2 80.00 usec
PL2 0.00 dB
PL12 16.50 dB
PL13 17.00 dB
PL2W 24.54113007 W
PL12W 0.54940748 W
PL13W 0.48965994 W
SFO2 500.1325006 MHz
SI 131072
SF 125.7577807 MHz
WDW EM
SSB 0
LB 0.30 Hz
GB 0
PC 1.40

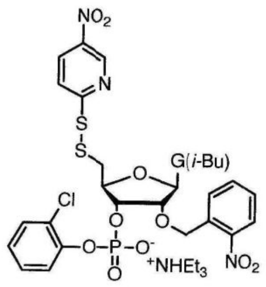

23

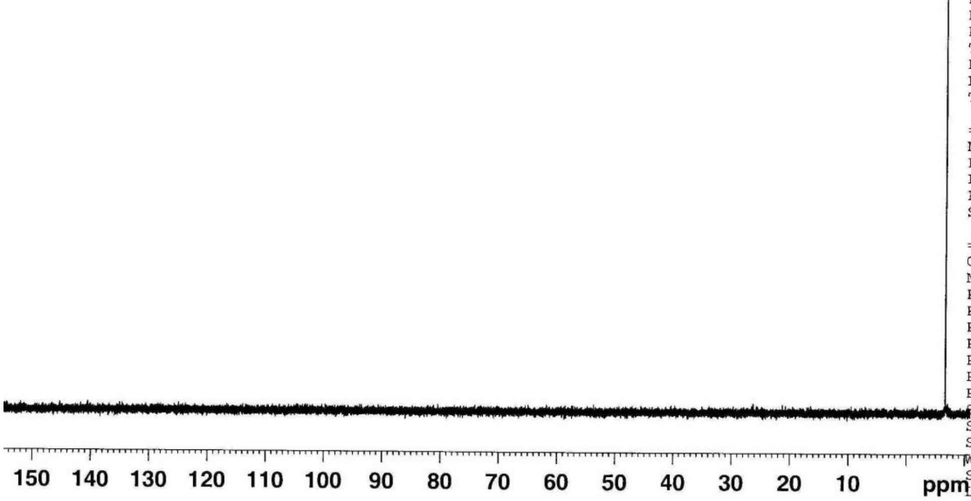

NAME	P-11-060.31P
EXPNO	1
PROCNO	1
Date_	20120511
Time	13.30
INSTRUM	spect
PROBHD	5 mm PAQNP 1H/
PULPROG	zgpg
TD	288456
SOLVENT	CDCl3
NS	4
DS	0
SWH	48076.922 Hz
FIDRES	0.166670 Hz
AQ	2.9999924 sec
RG	2890
DW	10.400 usec
DE	6.00 usec
TE	297.1 K
D1	3.00000000 sec
D11	0.03000000 sec
TD0	1

======== CHANNEL f1 ========

NUC1	31P
P1	11.00 usec
PL1	3.00 dB
PL1W	41.92221451 W
SFO1	202.4706373 MHz

======== CHANNEL f2 ========

CPDPRG2	waltz16
NUC2	1H
PCPD2	100.00 usec
PL2	120.00 dB
PL12	16.50 dB
PL13	17.00 dB
PL2W	0.00000000 W
PL12W	0.54940748 W
PL13W	0.48965994 W
SFO2	500.1325000 MHz
SI	32768
SF	202.4563350 MHz
WDW	EM
SSB	0
LB	0.00 Hz
GB	0
PC	1.40

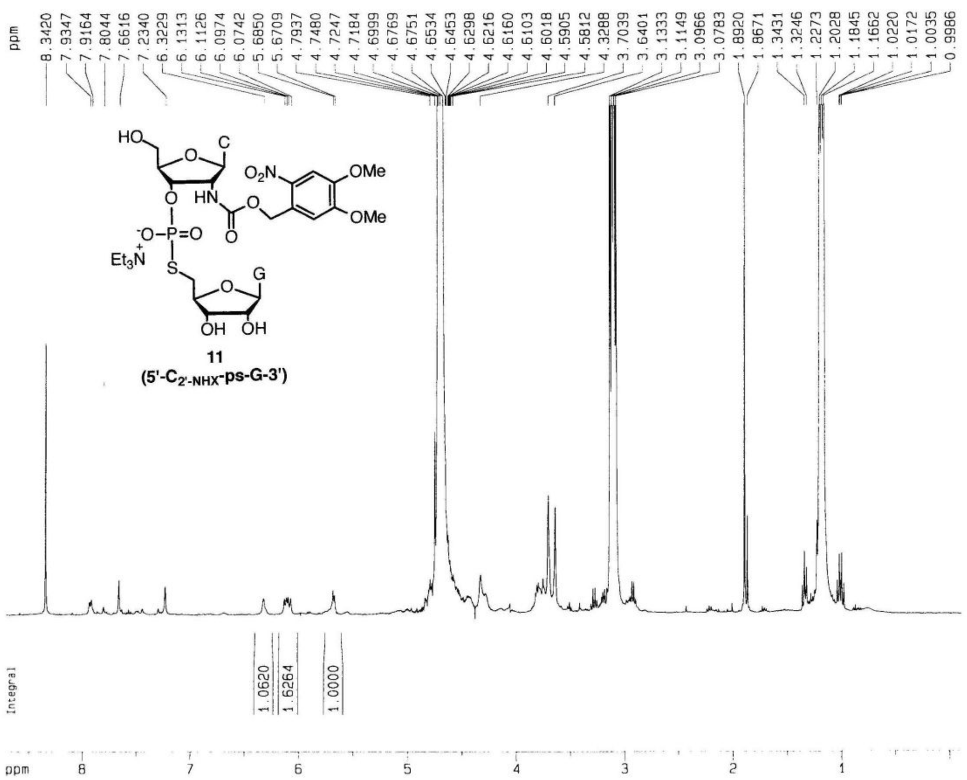

Current Data Parameters
NAME P-12-097.1H
EXPNO 1
PROCNO 1

F2 - Acquisition Parameters
Date_ 20080502
Time 14.51
INSTRUM spect
PROBHD 5 mm Multinu
PULPROG zg
TD 32768
SOLVENT ~~CDCl3~~ D_2O
NS 16
DS 0
SWH 5580.357 Hz
FIDRES 0.170299 Hz
AQ 2.9360628 sec
RG 57
DW 89.600 usec
DE 7.00 usec
TE 300.0 K
D1 2.00000000 sec
P1 7.70 usec
SFO1 400.1317512 MHz
NUC1 1H
PL1 -6.00 dB

F2 - Processing parameters
SI 32768
SF 400.1299990 MHz
WDW EM
SSB 0
LB 0.30 Hz
GB 0
PC 1.00

1D NMR plot parameters
CX 20.00 cm
F1P 8.717 ppm
F1 3487.74 Hz
F2P -0.103 ppm
F2 -41.27 Hz
PPMCM 0.44098 ppm/cm
HZCM 176.45053 Hz/cm

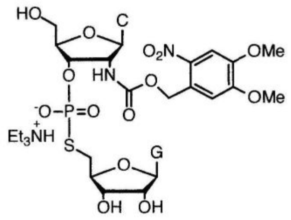

11
(5'-$C_{2'-NHX}$-ps-G-3')

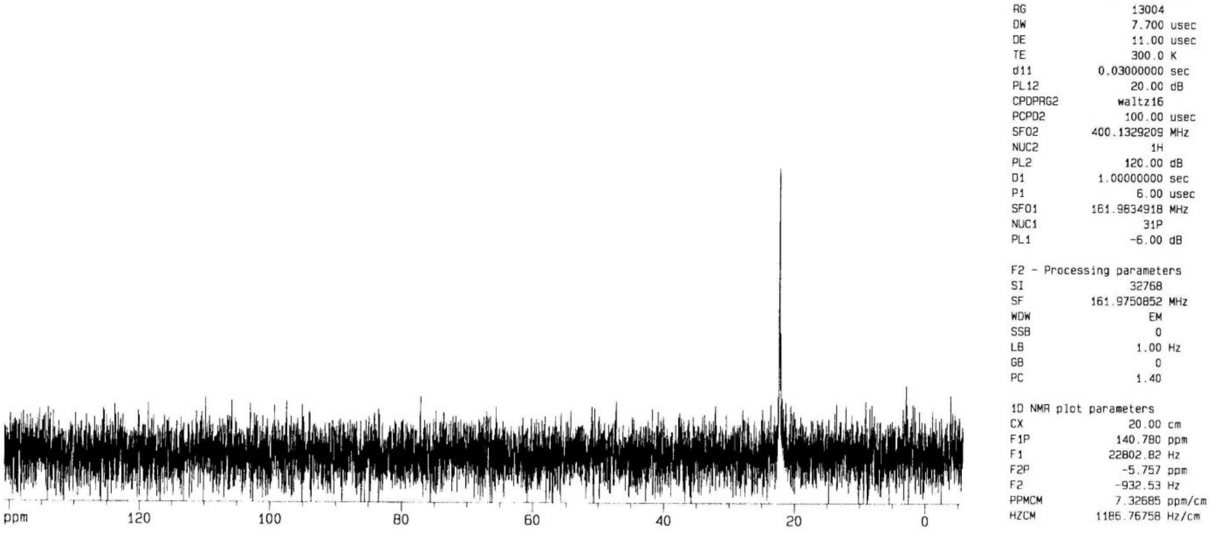

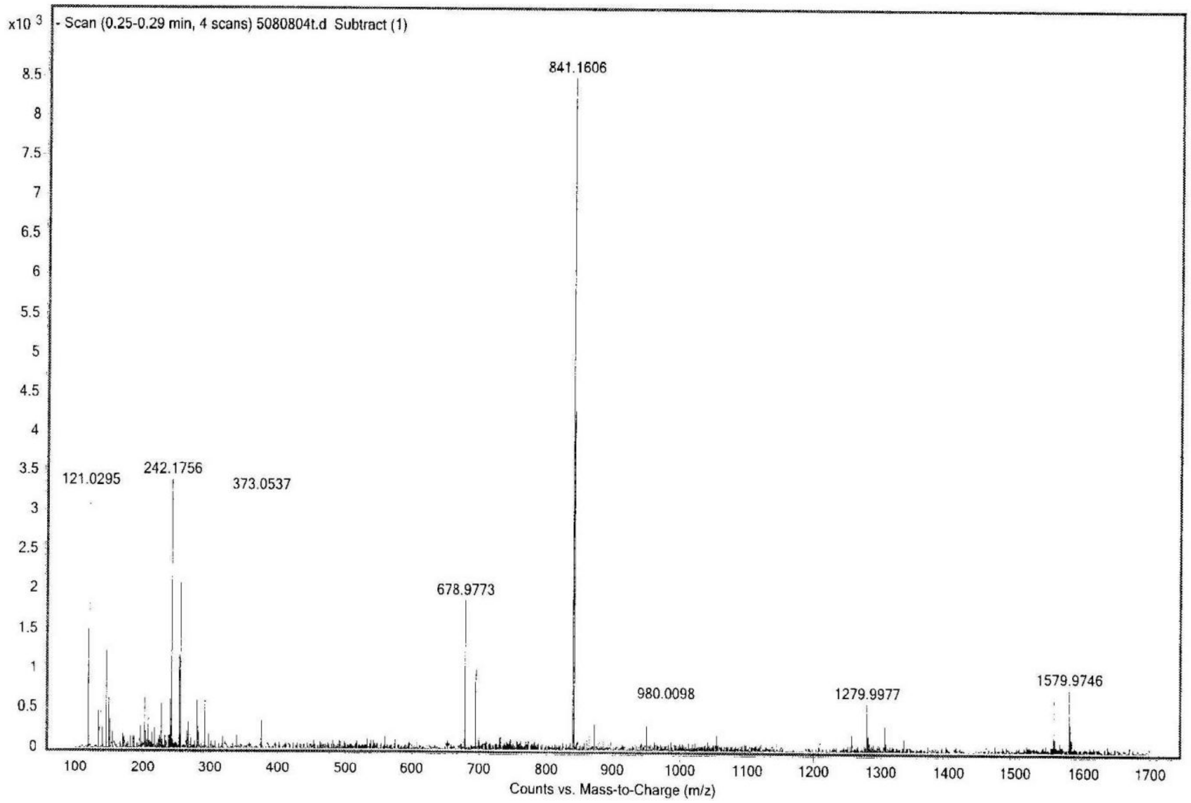

Measured Mass 841.1606

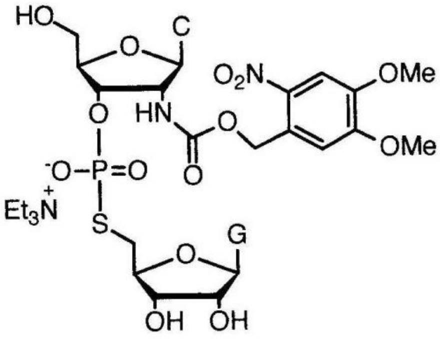

11
(5'-$C_{2'\text{-NHX}}$-ps-G-3')

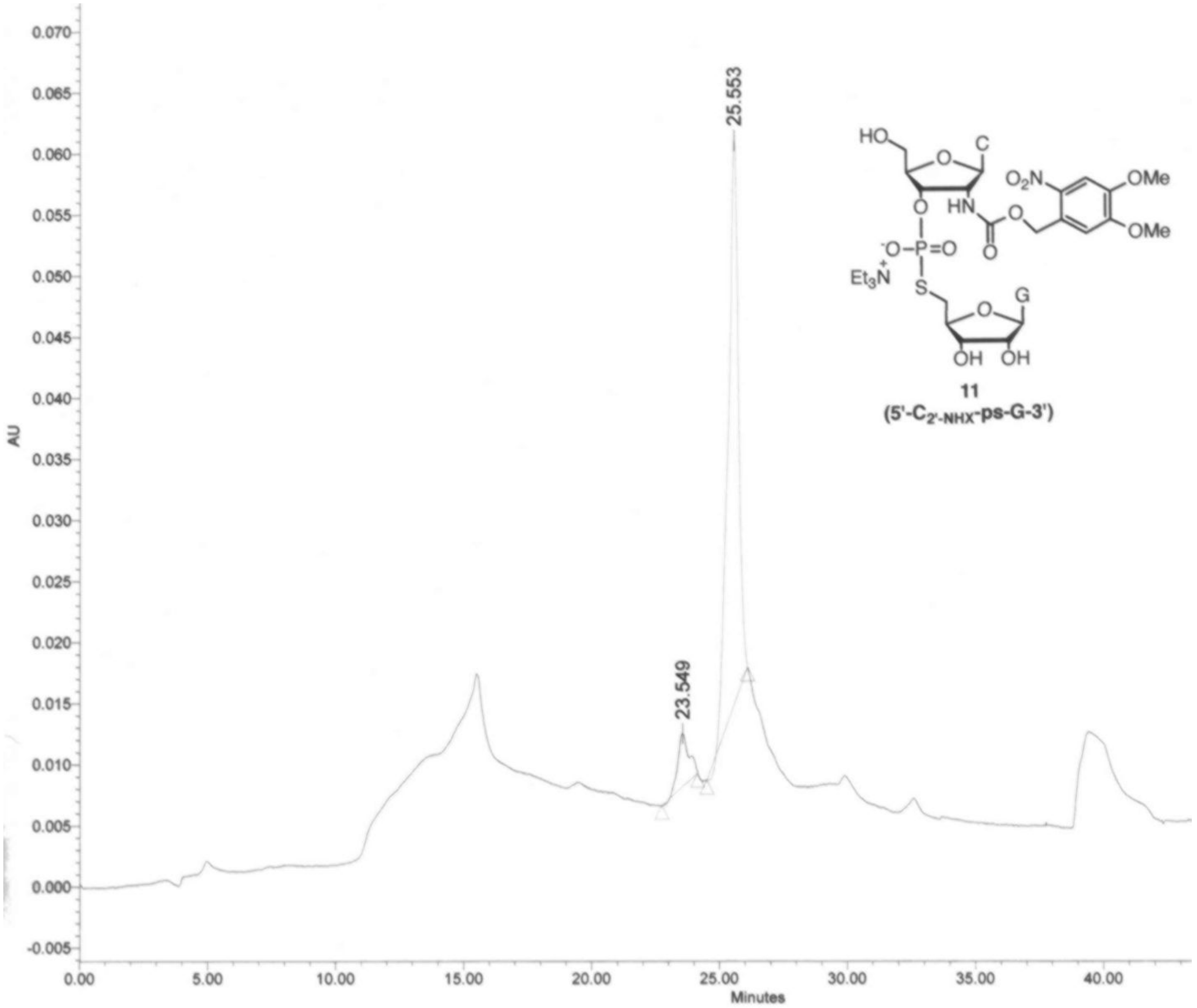

Reverse phase HPLC profile of **11:** The purity of **11** is estimated to be ~90% by reverse phase C18 column (HPLC conditions: Thermo Scientific Acclaim C18, 5 µm 120 Å 4.6 x 250 mm column; flow rate: 1.0 mL/min; buffer A, 0.1 M TEAA, pH 7; B, acetonitrile; 0-5 min, 100% A, 0% B; 5-35 min, 70% A, 30% B; 35-37 min, 0% A, 100% B; 37-41 min 0% A, 100% B; 41-43 min, 100% A, 0% B) with retention time 25.6 min.

Applied Biosystems Voyager System 6187

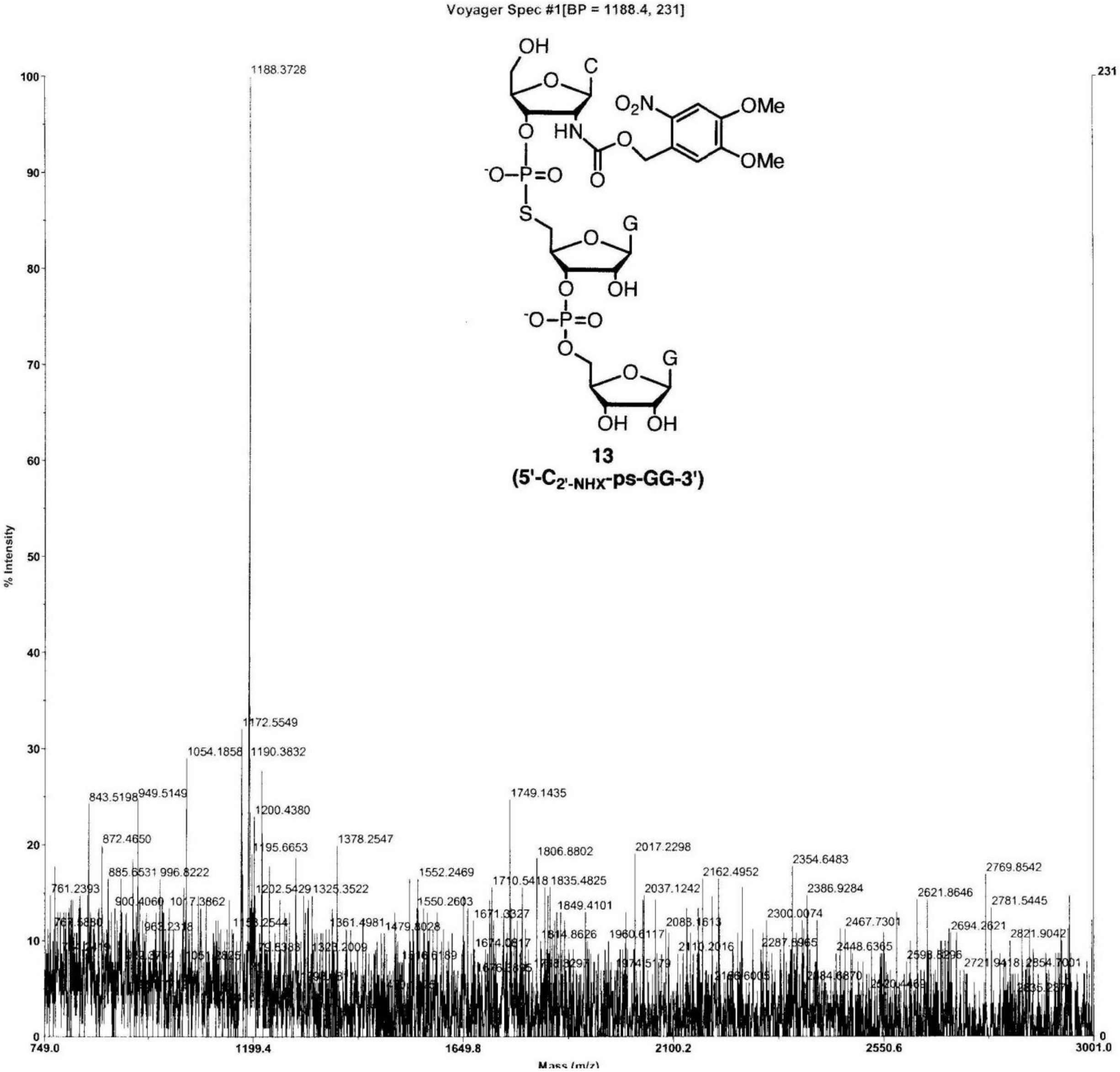

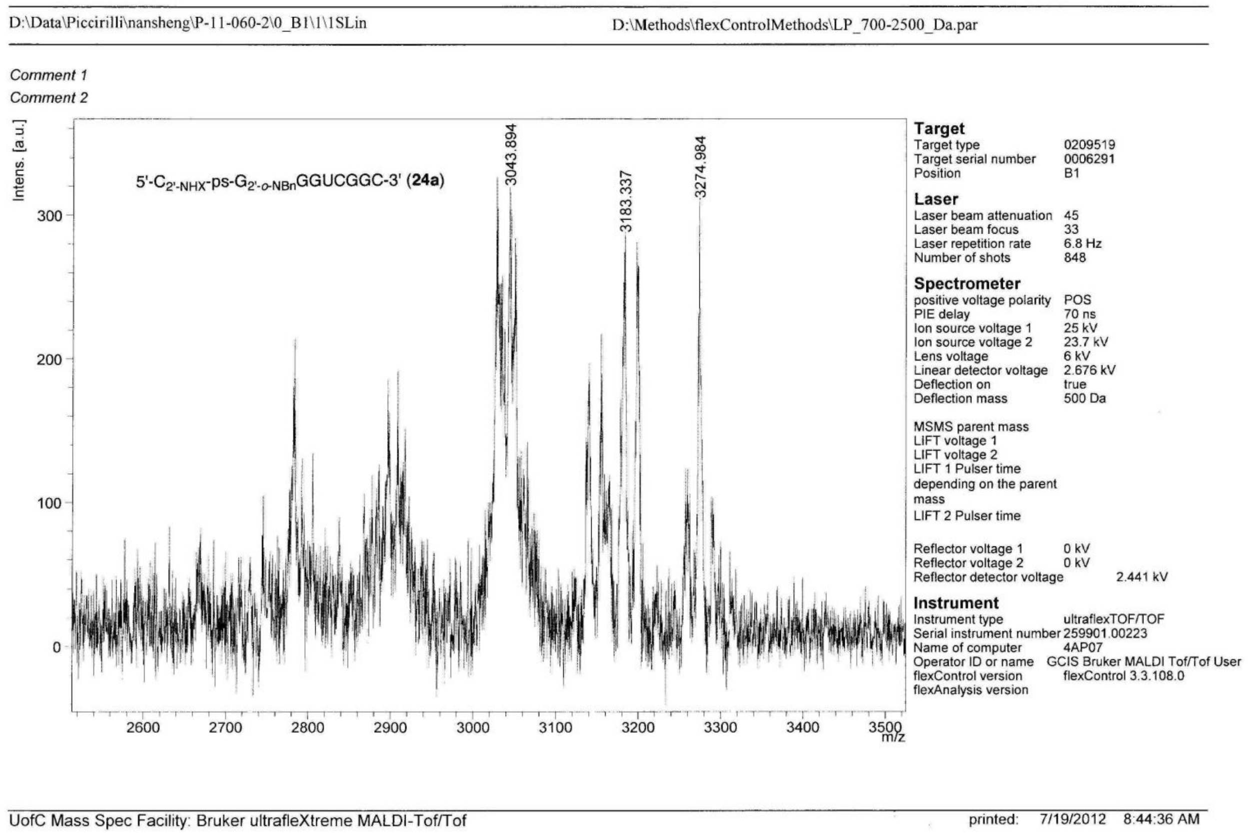

Note: calcd for [MH^+]: 3274.5, found: 3275.0; calcd for [$M+2Na^+-(C_7H_6NO_2)$]: 3183.4, found: 3183.3; calcd for [$MNa^+-(NC_{10}H_{10}NO_6)$]: ~3042.3, found 3043.9.

D:\Data\Piccirilli\nansheng\P-11-064-2-Ra\0_A3\1\1SRef D:\Methods\flexControlMethods\RP_700-3500_Da.par

Comment 1
Comment 2

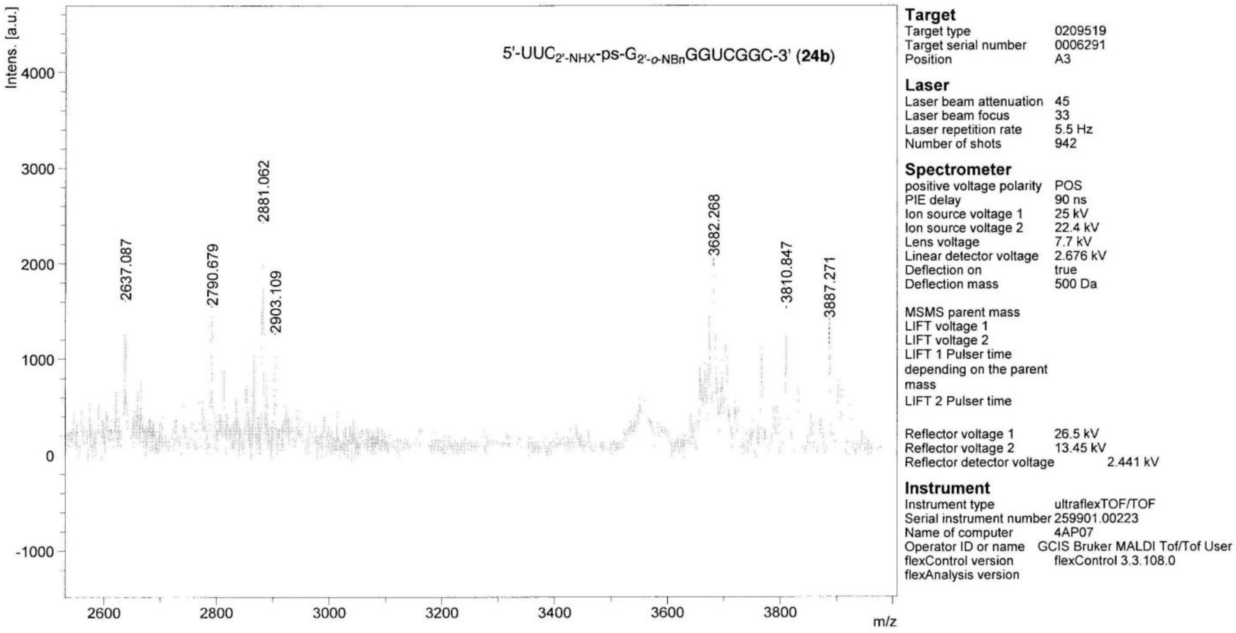

Target
Target type 0209519
Target serial number 0006291
Position A3

Laser
Laser beam attenuation 45
Laser beam focus 33
Laser repetition rate 5.5 Hz
Number of shots 942

Spectrometer
positive voltage polarity POS
PIE delay 90 ns
Ion source voltage 1 25 kV
Ion source voltage 2 22.4 kV
Lens voltage 7.7 kV
Linear detector voltage 2.676 kV
Deflection on true
Deflection mass 500 Da

MSMS parent mass
LIFT voltage 1
LIFT voltage 2
LIFT 1 Pulser time depending on the parent mass
LIFT 2 Pulser time

Reflector voltage 1 26.5 kV
Reflector voltage 2 13.45 kV
Reflector detector voltage 2.441 kV

Instrument
Instrument type ultraflexTOF/TOF
Serial instrument number 259901.00223
Name of computer 4AP07
Operator ID or name GCIS Bruker MALDI Tof/Tof User
flexControl version flexControl 3.3.108.0
flexAnalysis version

UofC Mass Spec Facility: Bruker ultrafleXtreme MALDI-Tof/Tof printed: 7/19/2012 9:07:23 AM

Note: Calcd for [MH^+]: 3886.6, found 3887.3; Calcd for [$M+2Na^+-(C_6H_4NO_2)$]: 3809.5, found: 3810.8; calcd for [$M+K^+-(C_{10}H_{10}NO_6)$] ~3684.4, found: 3682.3.

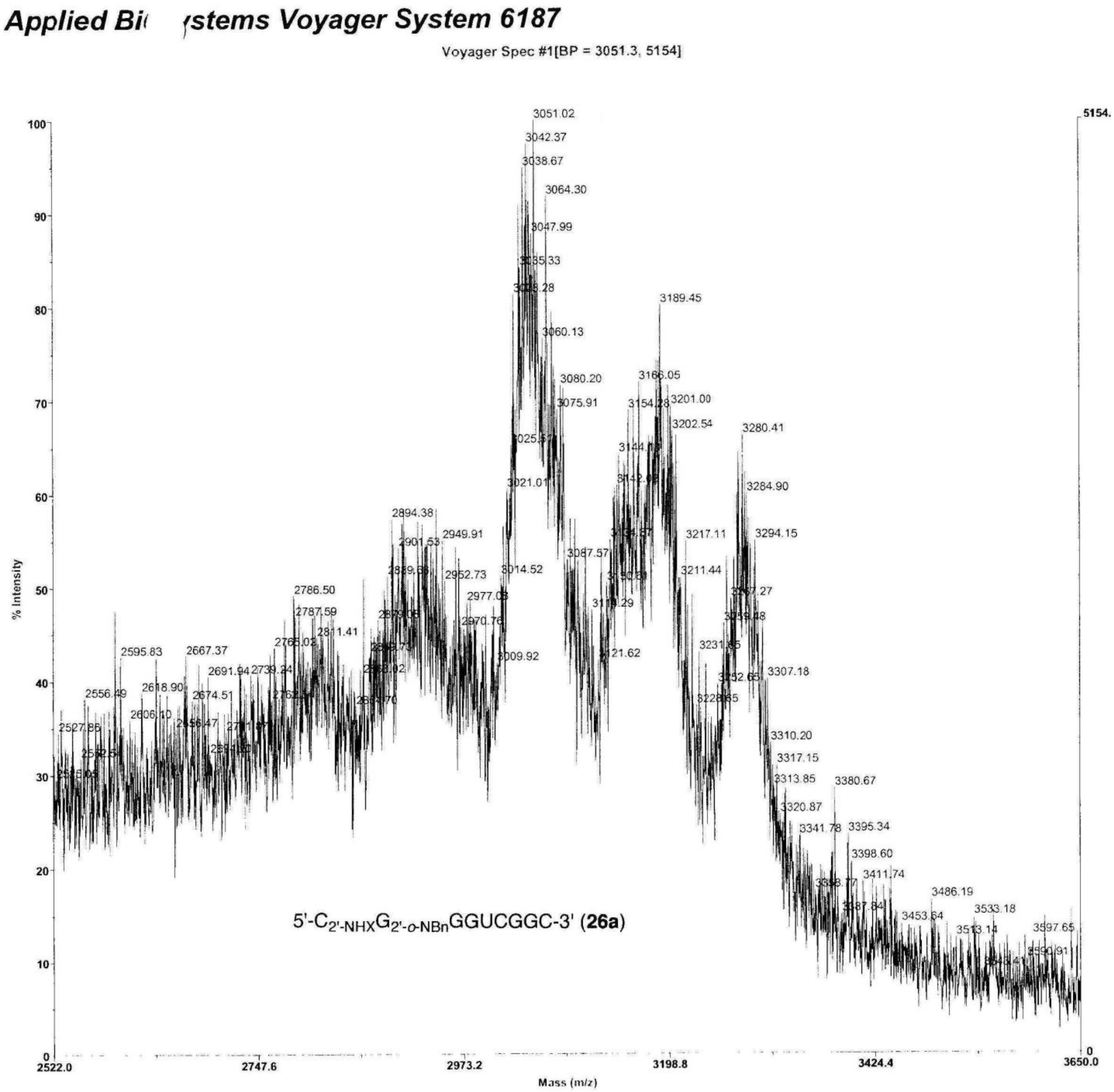

Note: Calcd for [MNa$^+$]: 3280.5; calcd for [MNa$^+$-($C_7H_6NO_2$)]: 3144.4 and calcd for [MNa$^+$-($C_{10}H_{10}NO_6$)]: 3040.3.

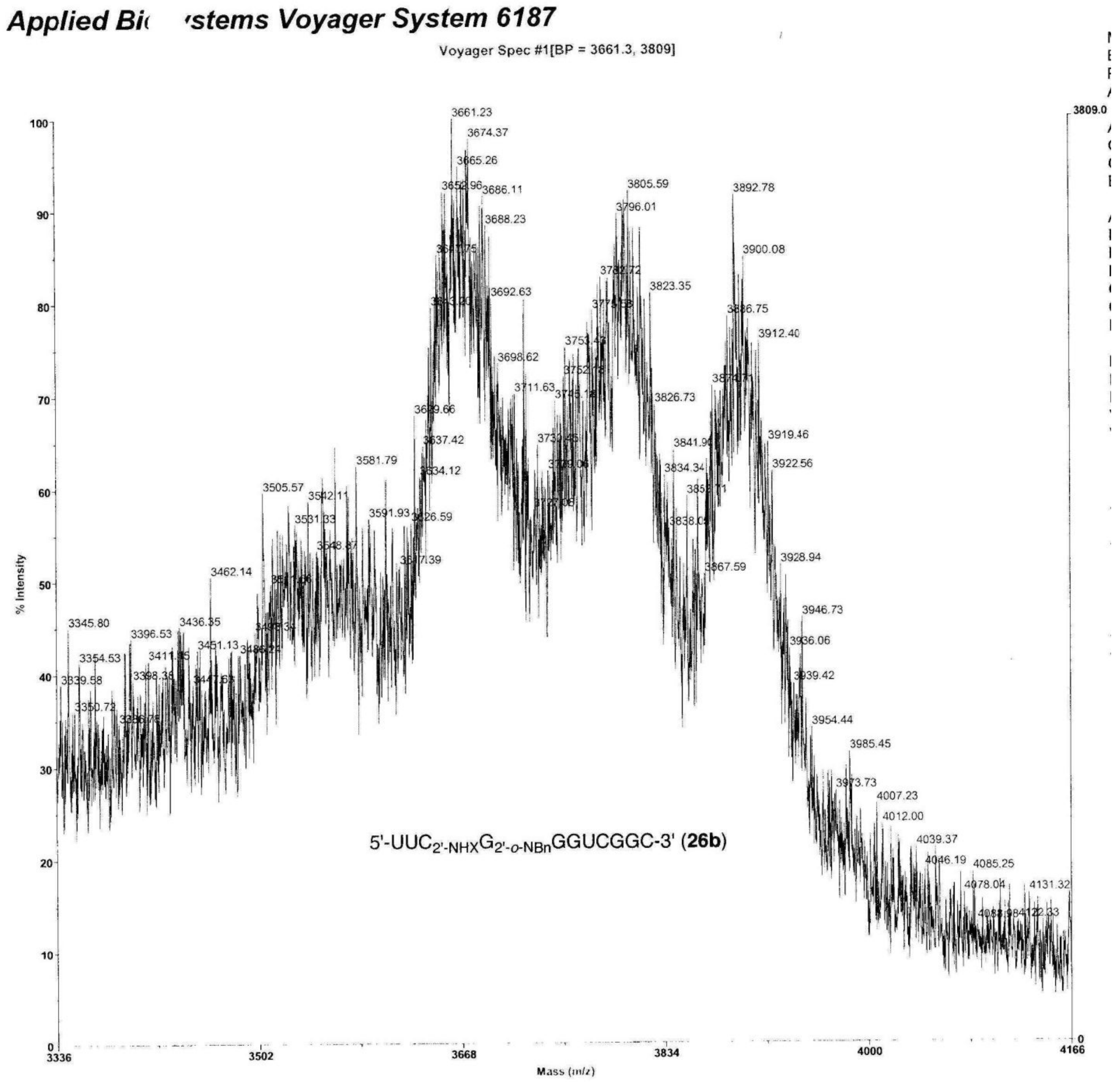

Note: Calcd for [MNa^+]: 3892.6; calcd for [MNa^+-($C_7H_6NO_2$)]: 3756.5 and calcd for [MNa^+-($C_{10}H_{10}NO_6$)]: 3652.4.

Scheme S1. Anti-genomic HDV ribozyme bounding to the modified RNA substrate.

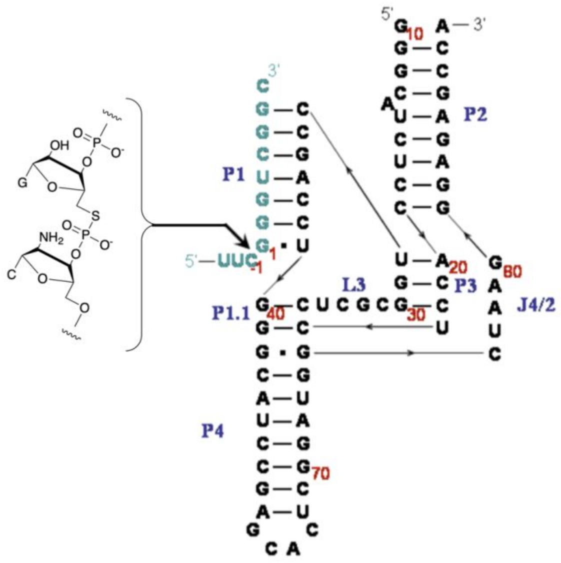

C_{-1} is 2′-aminocytidine and G_1 is 5′-S-G.

Figure S1. The representative gel image of the kinetic cleavage of **24b** in the absence and presence of HDV ribozyme at a buffer of pH 6.54.
$k_{hydrolyis}$ 2.17 x 10^{-2} min^{-} 1, k_{HDV} 4.20 x10^{-3} min^{-1}

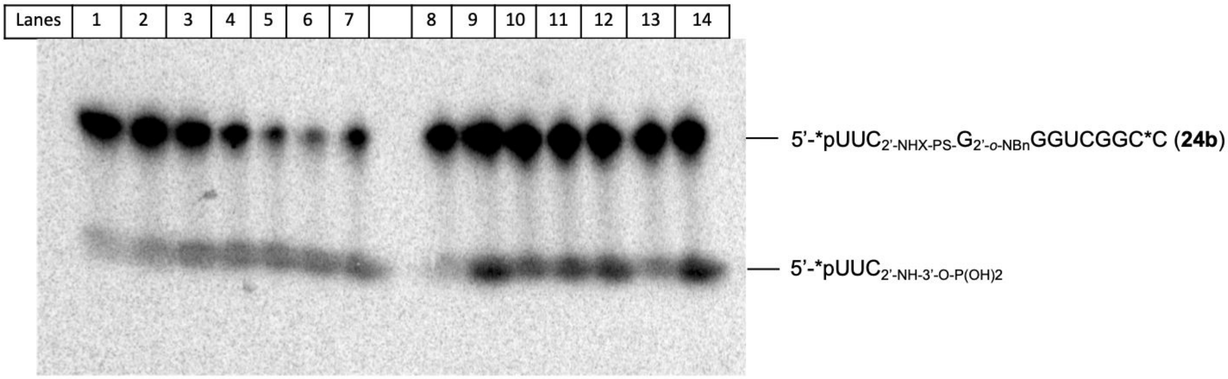

Lanes 1-7: **24b** cleavage without ribozyme: 3.5 min, 8 min, 20 min, 45 min, 90 min, 150 min, 240 min.
Lanes 8-14: **24b** cleavage in the presence of ribozyme: 3.5 min, 150 min, 20 min, 45 min, 90 min, 8min, 240 min.
